# Polypharmacy Definitions for Multimorbid Older Adults Need Stronger Foundations to Guide Research, Clinical Practice and Public Health

**DOI:** 10.3390/pharmacy7030126

**Published:** 2019-08-29

**Authors:** Caroline Sirois, Nelia Sofia Domingues, Marie-Laure Laroche, Arsène Zongo, Carlotta Lunghi, Line Guénette, Edeltraut Kröger, Valérie Émond

**Affiliations:** 1 Department of Social and Preventive Medicine, Faculty of Medicine, Laval University, Québec, QC G1V 0A6, Canada; 2 Centre of Excellence on Aging of Quebec, CIUSSS-CN, Québec, QC G1S 4L8, Canada; 3 Centre de Recherche sur les Soins et les Services de Première Ligne de L’Université Laval, Québec, QC G1J 0A4, Canada; 4 Institut National de Santé Publique du Québec, Québec, QC G1V 5B3, Canada; 5 Service de Pharmacologie, Toxicologie et Pharmacovigilance, Centre Régional de Pharmacovigilance, de Pharmacoépidémiologie et D’information sur les Médicaments, Centre Hospitalier Universitaire de Limoges, 87042 Limoges, France; 6 INSEM 1248, Faculté de Médecine, Université de Limoges, 87032 Limoges, France; 7 Faculty of Pharmacy, Université Laval, Québec, QC G1V 0A6, Canada; 8 Santé des Populations et Pratiques Optimales en Santé, Centre de Recherche du CHU de Québec, Québec, QC G1S 4L8, Canada; 9 Department of Nursing, University of Quebec at Rimouski, Lévis, QC G6V 0A6, Canada; 10 Centre de recherche du CISSS de Chaudière-Appalaches, Lévis, QC G6V 3Z1, Canada

**Keywords:** polypharmacy, older adults, multimorbidity, population health, research, clinical practice, medications, scoping review

## Abstract

There are numerous definitions of polypharmacy to describe the use of many medications among older adults, but there is a need to clarify if they are purposive and meaningful. By means of a systematic review, we identified definitions of polypharmacy used in multimorbid older adults (≥65 years). We evaluated if the definitions align among the domains of research, clinical practice, and public health and appraised whether concepts of polypharmacy are based on strong foundations. More than 46 definitions of polypharmacy were retrieved from 348 publications (research: *n* = 243; clinical practice: *n* = 88; public health: *n* = 17). Several thresholds based on the number of medications were mentioned. The majority of the publications (*n* = 202, 58%) used a minimal threshold of five medications. Heterogeneous qualitative definitions were identified, mostly stating that polypharmacy is “more drugs than needed”. There was no significant divergence between domains as to the type of definitions used, although qualitative definitions were more common in clinical practice. Nearly half (*n* = 156, 47%) of the publications provided no justification for the polypharmacy definition used. The wide variety of definitions for polypharmacy precludes comparisons, appropriate identification and management of polypharmacy in multimorbid older adults. Standardized definitions would allow more coherent judgments regarding the individual and collective stakes of polypharmacy.

## 1. Introduction

As chronic diseases tend to accumulate over a lifetime, the ageing of the population has caused multimorbidity to become a significant condition [1]. Caring for those chronic diseases often relies on pharmacologic treatment, leading to a substantial number of older adults using many medications concomitantly [2,3]. Treatment of acute conditions can further complicate therapy, resulting in a large number of medications. For example, Canadians 65 years and older were prescribed on average 6.9 different medication classes in 2016, and 26.5% were prescribed at least 10 [4]. The use of polypharmacy in older adults and interventions to improve this condition have thus been a long-standing concern [5,6].

Nonetheless, portraying the frequency and consequences of polypharmacy in multimorbid older adults remains challenging since there is no consensus definition for this term. Such a definition would be useful to understand the association between using many medications and health outcomes and to develop interventions designed to tackle inappropriate medication use. It would also facilitate the comparison of studies on polypharmacy in multimorbid older adults and hence help determine, for instance, how significant polypharmacy is as a public health issue. However, a universal and unique definition of polypharmacy may not be possible as it may depend on the aim it is intended to achieve [7].

Indeed, the domains of research, clinical practice and public health, all of which must address the issues of polypharmacy in multimorbid older adults, have different needs and visions that could lead to different definitions of polypharmacy. Research activities focus notably on establishing etiological links between multiple medication use and health events. Therefore, the definitions of polypharmacy must be precise and allow for reproducibility. Clinical practice is based on research findings to establish standards of practice for the care of older adults. However, in this case, polypharmacy should probably be defined less comprehensively than for research, to allow a simple application in the clinical reality. Finally, for public health to be able to conduct its activities such as describing population health status in multimorbid older adults and assisting in resource planning, the definitions of polypharmacy should provide a clear picture of the associations between polypharmacy and health outcomes and use of health services. Thus, a certain variety of definitions of polypharmacy is expected. Although some publications have inventoried the definitions of polypharmacy in older adults [8,9,10,11], there is still a need to clarify whether or not these definitions are purposive and meaningful, that is if they align with the overall goals they intend to achieve, and if they are based on conceptual frameworks or stem from sound methodologies well suited to the aims.

Therefore, the objectives of this review were: (1) To identify the definitions of polypharmacy that are used in the context of multimorbidity among older adults (≥65 years) in the domains of research, clinical practice and public health; (2) to establish on what basis (theoretical/conceptual, methodological, previous research) these definitions were constructed or chosen. The final results of this work allowed us to highlight the knowledge gaps, the problems and the possible strengths of the identified definitions and to suggest which avenues should be privileged to advance towards consensus definitions for polypharmacy in multimorbid older adults.

## 2. Materials and Methods

The complete protocol for our review has been registered in Prospero (CRD42014014989) and previously published [12]. The systematic review had three main objectives and this article refers to the first of three objectives, namely the review of the definitions used in multimorbid older adults. We herein briefly summarize the method used.

### 2.1. Participants/Population

We considered all publications that targeted people aged 65 years and older with at least two concurrent chronic diseases (multimorbidity). We included publications if (1) at least 80% of participants were aged 65 years and older, (2) the data from people aged 65 years and older could be extracted, or (3) the focus of the publication was older adults (for reviews or other type of publications that did not recruit patients). We included all settings (community, hospital, nursing homes) and types of health care (public, private, others).

### 2.2. Searches

A systematic search strategy was developed by the authors (CS, VE) in collaboration with an experienced librarian (V. Tessier). A second librarian specialized in health sciences revised the proposed strategy (S. Vachon). A first search was performed in December 2014 and was updated in May 2016 using the ovidSP (Bouquet total access collection, EBM Reviews, Embase, Global Health, Medline) and EbscoHost (AgeLine, CINAHL, Health Policy Reference Center, Medline, Public Affairs Index, SocINDEX) platforms. We also searched Google Scholar and Google to identify grey literature such as governmental reports.

Four concepts were considered in our search strategy: Polypharmacy, older adults, multimorbidity and positive/negative outcomes (the latter referring to the other objectives of the systematic review, whose results will be reported elsewhere). The search strategy was adapted to the syntax requirements of each database (use of different thesaurus terms, truncation and wildcard characters) [12]. We included documents published between 2004–2016 in all languages. We also hand searched the bibliographies of all included publications to retrieve documents that had not been identified through the databases searches.

### 2.3. Types of Publications Included

No restrictions were placed on the types of publications. Original studies, reviews, commentaries, editorials, practice guidelines, reports, theses and governmental publications were used to identify polypharmacy definitions and were searched for the primary references they cited. In cases of missing information, we contacted the authors to complete the required information if the publication corresponded to the inclusion criteria.

### 2.4. Selection of Publications

We used Endnote^®^ to group the results and exclude duplicated publications. Two independent reviewers examined the titles and screened the abstracts (M. Dugas, CS). Full-text publications were retrieved for all publications not excluded during the previous two steps. Full texts were reviewed by two independent reviewers (CS and NSD/AZ). All exclusion criteria were recorded at each step to generate flow charts according to Preferred Reporting Items for Systematic Reviews and Meta-Analyses (PRISMA) requirements.

Publications were excluded based on the following criteria:

(1) The publication did not provide any operational definition of polypharmacy. For example, defining polypharmacy only as “large number of medications” did not qualify. To be considered operational, the definition could involve a specific number of medications (e.g., 5 or more) or indicate a specific condition (e.g., complex medication regimen with at least one inappropriate medication).
(2) The targeted population did not include people 65 years and over.
(3) The publication referred to polypharmacy used for the treatment of a single medical condition in the absence of multimorbidity.

Publications were also excluded if there was missing information (e.g., abstracts were excluded if required information was not included in the abstract and no further information was available after contacting the authors); the document was published before 2004; the document was published in a language in which our team was not fluent (English, French, Spanish, German, Portuguese, Arabic, Italian) and we did not find adequate translation resources; or it was a duplicate publication (publication summarizing another published article, duplicates of included studies, response letters to published articles).

### 2.5. Data Extraction

Two independent reviewers (NSD/AZ, CS) conducted a full-paper evaluation and data extraction. In the event of disagreement that could not be resolved by discussion, a third reviewer was invited to make a final decision. Established data extraction forms adapted for the objectives of the review were created using FileMaker^MD^ (https://www.filemaker.com). This allowed all researchers to access the data in real time.

For each publication, we recorded bibliographic details, type of publication, context, participants and the definition of polypharmacy. We then extracted information about the definition of polypharmacy, the types of medications included (including prescribed or not/regular or as needed/oral or other route of administration), the counting method and qualitative criteria (e.g., inappropriate medication) used in order to label a medication regimen as polypharmacy. We compared the definitions used in the publications according to whether the publication pertained to domains of clinical practice (such as expert opinions, clinical guidelines, narrative reviews, editorials), research (cohort/case-control studies with clinical or epidemiologic perspectives/or methodological papers) or public health and surveillance (such as development of quality indicators, national initiatives for population-based interventions, or population-based research with public health decision-making perspective). We also evaluated whether definitions (1) relied on a theoretical framework (such as a conceptual model, which could address elements of pharmacology including interactions, duplications or quality of treatments according to different criteria) or resulted from a methodological assessment (such as receiver operating [ROC] curves to assess the ability of medication thresholds to accurately predict health events); (2) referred to other published material; or (3) were reported without specific foundation provided for their choice. We grouped definitions according to their nature [quantitative (numerical definitions), qualitative (including a notion of quality of prescribing)].

## 3. Results

**Figure 1:**
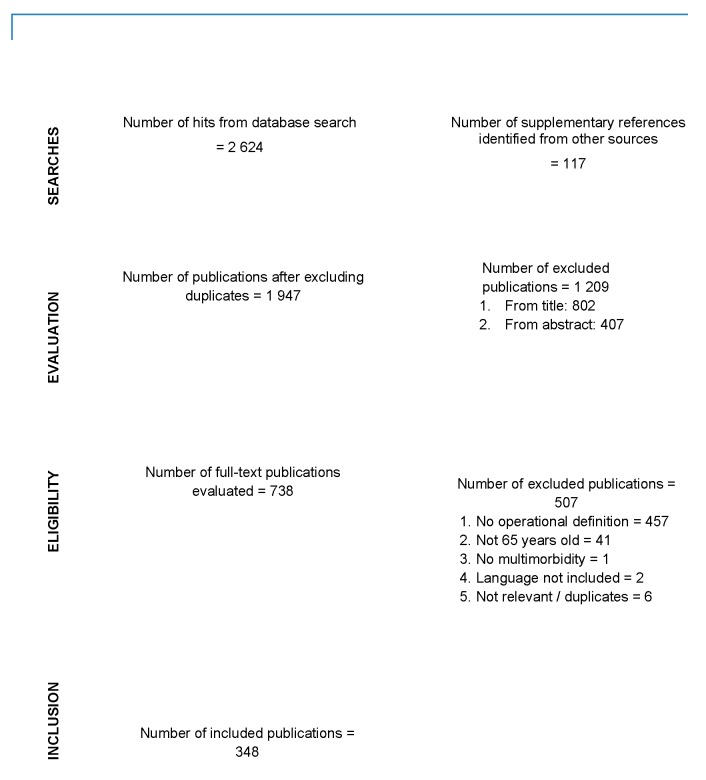
Flow chart of bibliographic search.

Our bibliographic search resulted in 2624 publications, of which 231 were included. Reference searching and evaluation of grey literature provided a further 117 publications (Figure 1). In total, 243 publications pertained to research, 88 to clinical practice and 17 to public health. Figure S1 in the supplementary presents the characteristics of the included publications and the respective references.

### 3.1. Definitions Retrieved

All definitions reported in the publications were collected. The assessment of publications did not reveal particular trends in definitions reported in publications over the years, although the limited period of a decade may not have been sufficient to identify them. Quantitative definitions of polypharmacy (without notions of prescribing quality) were the most frequently documented (Table 1). They appeared in 97.1% (236/243) of publications pertaining to research, 93.2% (82/88) of those related to clinical practice and in all public health publications (17/17). The thresholds or categories for defining polypharmacy varied widely, resulting in 37 different definitions. When grouping definitions with similar minimal thresholds, cut-offs of five or more medications (*n* = 202; 58%) and 10 or more medications (*n* = 22; 6%) were the most popular. The definition cut-offs differed according to setting. While the minimal threshold of five medications was clearly the most frequent for research done in community (70%) and hospital (68%), research conducted in nursing homes used varied minimal cut-offs such as five (38%), six (33%) and nine medications (21%). Qualitative definitions were mentioned in 58 publications (16.7%), either independently or in addition to a quantitative element. A total of 13 (5.3%) research, 42 (47.7%) clinical practice (mostly reviews) and three (17.6%) public health publications contained such definitions (Table 2). We identified heterogeneous definitions. The most frequent qualitative-only definitions stated that polypharmacy was present if more medications than needed were used. Clinical judgement or individual assessment is required for most of the retrieved qualitative definitions (e.g., applying Beers criteria, deciding if the medication is duplicated or inappropriate); this may explain why they were reported more frequently in clinical publications rather than in research or public health publications.

Several pieces of information relevant to the conceptualization of polypharmacy were missing from the quantitative definitions found (Table 3). In the research domain, 41% of the publications did not state the type of medications included (e.g., prescribed, over the counter (OTC), etc.), 72% did not elaborate on the inclusion of medications for chronic or as needed use, and 89% did not specify whether they included non-oral medications. The medication counting method (simultaneous use, continuous use or cumulative exposure over a period) was not reported in 30% of cases. The proportion of clinical practice publications not reporting relevant information was higher, ranging from 57% (medication counting method) to 99% (route of administration). Public health publications contained more consistent methodological descriptions (omissions between 18% [type of drugs] to 71% [route of administration]), with few publications that did not detail the procedures used to calculate the number of medications. Publications describing indicators of polypharmacy generally provided more comprehensive detail. For instance the indicator developed by the Aged Care Branch in Australia (nine or more different medications) specified that medications included prescribed, OTC and as needed medications; medications administered by oral, intramuscular, intravenous, subcutaneous, rectal and vaginal routes; with the exclusion of topical medications, lotions/cream/ointments used in wound care and eye drops [13].

### 3.2. Methodological Foundation or Conceptual Basis Underpinning Definitions

From the 329 publications that stated a specific definition, 192 (58.4%) either stated a foundation or underpinning basis to their definition (Table 4). Examples of definitions based on theoretical/ methodological grounds included those who used statistical methods (e.g., ROC curves) to identify the optimal threshold to predict health outcomes (outcome-oriented thresholds) and those who used the medication distribution in their sample to determine percentiles (data-oriented thresholds). Interestingly, the 157 publications that referred to other material did so by quoting a large corpus: 125 different sources were cited; the nine publications most frequently quoted [9,14,15,16,17,18,19,20,21] were cited between 6 to 11 times each. There were no statistical differences between the domains of research, clinical practice and public health as to the proportion of publications not justifying their choice of definitions (chi-squared test *p* = 0.168). In order to restrict results more closely to publications where polypharmacy was the main subject, we analyzed the 199 publications where the term polypharmacy appeared in the title: 73 of them (36.7%) did not provide information to justify their choice of a definition.

## 4. Discussion

Our systematic review demonstrates the manifold definitions of polypharmacy used in populations of older adults with multimorbidity, frequently without a strong basis or conceptual foundation. Given such arbitrary formulations, there is very little convergence between publications.

Others have identified a wide variety of definitions of polypharmacy among older adults [8,9,10,11]. Following a protocol similar to the one we published on Prospero in 2014, Masnoon et al. listed 138 definitions of polypharmacy and related terms from 110 documents published in English between 2000 and 2016 [8]. Although we have included more publications (348) in our review because of a wider array of document types and non-English publications, their work contains more definitions than ours as we have not detailed all the particularities of these definitions. For example, when it referred to a similar medication threshold (e.g., the use of 10 medications) we considered the definition to be the same, although some authors characterized it specifically as severe polypharmacy or as excessive polypharmacy. However, our review provides a unique view of polypharmacy definitions through a critical appraisal based on the needs and purposes that the definitions must fulfill in the domains of research, clinical practice and public health. This distinction allows to observe similarities of definitions across domains, and the necessity for research to provide definitions based on stronger methodological rationale to better meet the needs of clinical practice and public health. The most widely used definition of polypharmacy, namely the use of five medications or more (found in 58% of the publications that we evaluated and in 46% of those of Masnnon et al. [8]), could indeed not be the most appropriate in many circumstances. In fact, although the thresholds used to define polypharmacy increased with time, and although many authors now suggest using 10 or more medications to define polypharmacy for older multimorbid adults, we observed that most authors continue to use at least one definition of five medications or more. Even when using the terms hyperpolypharmacy or excessive polypharmacy, which are often classified as 10 medications and more, the term polypharmacy most often refers to using five medications or more in multimorbid older adults. It thus appears the variety of polypharmacy definitions may cause confusion and standard definitions would help better understand the issues related to polypharmacy among multimorbid older adults.

### 4.1. Examination of the Various Definitions Retrieved (Objective 1)

Several factors could explain the wide variety of definitions presented in the literature. First, as previously stated, the threshold for polypharmacy has evolved over time along with the concomitant increase in the number of medications used [22,23]. Currently, very few studies describe polypharmacy as the use of two, three or four drugs in older adults with multimorbidity. Of note, the increasing push towards deprescribing of inappropriate medications to reduce polypharmacy in older people and in those with limited life expectancy is likely to change the portrait in the next years. Second, the associations between cut-off values for numbers of medications and outcomes may differ according to the outcome in question (e.g., hospitalizations, quality of life, frailty), and across populations (e.g., older patients with cancer, men vs. women [24]). With this in mind, polypharmacy could—and perhaps should—take on different faces. Finally, the fact that definitions of polypharmacy are so varied and generally based on scant evidence likely contributes to a general notion that any definition may be equally appropriate. In fact, many of the publications we consulted through the first stage of the review contained polypharmacy in the abstract or as a keyword but did not address or mention the term polypharmacy in the text. This suggests a broad assumption that polypharmacy means “taking many medications”, without a need for further definition.

Many essential elements for a proper, clear and operational definition of polypharmacy were missing in the majority of the publications retrieved, which is particularly concerning for research. The number of medications involved, the type of medications (prescribed, OTC, etc.), the exposure length, or the distinction between acute and chronic polypharmacy, for example, were not often specified in the publications consulted. While it may be reasonable to omit such information in certain types of material (such as reviews or publications intended for clinical practice whose major focus is not polypharmacy), the degree of definitional rigor should be higher in research. However, the definition of polypharmacy remained vague in more than half of the research publications. One striking example is a study recruiting older people with polypharmacy without explicitly defining what polypharmacy entails [25]. Other authors have found a lack of rigor in the definition of polypharmacy: From the sixteen studies included in their systematic review of the impact of polypharmacy on health outcomes, Frazier et al. revealed that only seven defined polypharmacy [10]. This raises questions about the ability of research to adequately inform clinical practice and public health.

### 4.2. Issues Related to the Foundations that Support the Choice of Definitions (Objective 2)

The lack of a theoretical basis or conceptual model is a major quality gap for most definitions of polypharmacy in multimorbid older adults. Only 28 (11.5%) research publications provided such information. Even after restricting analysis to publications mentioning polypharmacy in their titles, the percentage providing such information remained low (17.3%). Moreover, the diversity and wide array of references that were used to refer to specific definitions of polypharmacy is revealing. This corroborates the notion that there is neither consensus nor a clear path to a standard definition. Interestingly, the references which Hoffmann et al. [26] quote to support the conclusion that there is no consensus definition of polypharmacy are the same references used by other authors to justify their choice of a cut-off of five or 10 medications. In fact, it is relatively easy for researchers to find a reference for the one definition they intend to use, thus justifying its use by prior use, without providing a specific rationale for their choice. Nonetheless, some differing references used to justify a given definition of polypharmacy may relate to differing contexts; for example, some authors cite research conducted in nursing homes, while others cite those pertaining to oncology. Very few provided specific reasons for choosing a specific definition. Hopcroft et al. [27], for example, stated that adverse effect occurrence would increase at the threshold they chose to define polypharmacy. It is also bears mentioning that some definitions relied on the distribution of medications in a given population [26,28,29]. This may result in different cut-offs in time for a population. Population distributions of medication use are also dependent on context (different prescribing habits and other health related characteristics); still, such definitions allow identifying proportions of individuals using the highest numbers of medications in a particular population. Other authors have assessed the relationships between number of medications and different health outcomes to define polypharmacy [15,30,31].

### 4.3. Contentious Aspects Related to the Definitions of Polypharmacy

The quantitative definitions (based on numerical thresholds) and qualitative definitions (incorporating a notion of quality) of polypharmacy are regularly opposed. We observed that publications from the clinical practice domain usually mention both positions. However, qualitative definitions remain rare in research publications. Considering the very strong association between the number of medications and the presence of inappropriate medications, concepts of polypharmacy and potentially inappropriate medications are entangled. Nonetheless, our review does not support the views of Hudhra et al. [32] who state that “most researchers agree that polypharmacy occurs when a medical treatment comprises at least one unnecessary medication”, as most researchers rely on definitions that do not involve such clinical judgment to evaluate the presence of polypharmacy.

Similarly, many authors state that it is problematic to define polypharmacy with a threshold because some older adults will need a large number of medications to be adequately treated [11,21,33]. Indeed, the presence of certain types of multimorbidity nearly invariably leads to polypharmacy [34]. However, as per the etymology, polypharmacy means many medications, even if they are all appropriate. Although polypharmacy has a negative connotation, it is not inevitably inappropriate. There is thus a need to distinguish between appropriate and inappropriate polypharmacy. Nevertheless, very high numbers are usually questionable; it would be quite rare that 15 medications, for example, would all be appropriate. As Bronskill et al. suggested, a threshold is not “an arbitrary quota, but rather [a] starting point for reviewing the concomitant use of unnecessary medications” [35], an idea well integrated in some publications [23]. Several authors have also suggested other terminologies, such hyperpharmacotherapy or multiple medication use [9], but the distinction with polypharmacy remains to be established appropriately.

### 4.4. Recommendations to Advance towards Consensus Definitions of Polypharmacy in Multimorbid Older Adults

Our review reveals that there are many knowledge gaps to fill, starting with the need for definitions allowing researchers to discern which combinations of treatments will result in adverse or beneficial effects in older adults with multiple chronic diseases. In fact, it remains a matter of concern that the definitions used in research suffer from several shortcomings. If clinical practice publications cannot be expected to provide important insights into such concepts as the type of drugs included or the route of administration, it would be crucial to document those elements in research, to ensure adequate integration of results in clinical practice and public health.

We suggest that future definitions of polypharmacy integrate the following basic elements, especially when the definition is used for research and public health interventions: The number and type of medications involved (prescribed, OTC, etc.), the exposure length, and the distinction between acute and chronic use of medications. Definitions should be based on a solid foundation and should serve the intended purpose. For example, a definition could be chosen on the basis that the medication threshold has been associated with an increased risk of adverse events in previous research, or that the use of health services increases significantly from this threshold. Definitions used in clinical practice should also be evidence-based to ensure credibility of the term “polypharmacy”. In fact, it is important for research to determine which health outcomes are associated with which type of polypharmacy. By ensuring reproducibility of results in different countries, settings and populations, it will be possible to develop standard definitions of polypharmacy that will ensure a better understanding of the issues at stake with polypharmacy. Such standard definitions could also provide other opportunities. For example, some authors have suggested that polypharmacy should be considered a disease in itself [36]. If polypharmacy were to be identified as a genuine condition, there could even be a proper International Classification of Diseases (ICD) code for the “disease”. This could lead to more appropriate identification of the problem, facilitate greater awareness for responsive patient assessment and care planning. However, standard definitions with solid evidence-based data are needed to make polypharmacy such a health condition.

### 4.5. Limitations of the Present Review

This study has some limitations. Our definitions of clinical practice, research and public health may be arguable, and some publications could fall into more than one category. Although we strived to include all publications mentioning polypharmacy, our search strategy may have omitted some relevant ones. Nonetheless, we did retrieve a very large number of publications, including documents published in different languages, and we do not expect that results might have changed if further publications were included. We did not update our literature review to include recently published documents, as the number of publications was already significant; but from our recent work on the topic of polypharmacy [37], there are no recent studies that would have been likely to change our conclusions. Formal quality assessment was not performed because the critical analysis of the definitions did not require an evaluation of the quality of the studies. In addition, we did not investigate outcomes as part of this specific review of definitions, but this will be evaluated in the context of the other objectives of our systematic review.

## 5. Conclusions

Polypharmacy in multimorbid older adults must be better defined according to its field of application. The panoply of definitions and means by which polypharmacy is conceptualized in research leads to much confusion surrounding the term, which hampers research from informing clinical practice and public health stakeholders adequately. There is an urgent need to better understand the basic pharmacological implications of using many medications in multimorbid older adults in order to evaluate the potential pitfalls (and benefits) of polypharmacy. At present, considering the variety of definitions used, it remains difficult to clarify what should be the best definitions of polypharmacy for multimorbid older adults. Most likely, several definitions will need to be employed to respond to the various imperatives of different domains, populations and settings.

## Figures and Tables

**Table 1:** Numbers and proportions of publications citing quantitative-only definitions of polypharmacy in multimorbid older adults according to domain †.

Definition (Cut-Off)	Research (*N* = 243) *n* (%)	Clinical Practice (*N* = 88) *n* (%)	Public Health (*N* = 17) *n* (%)	TOTAL (*N* = 348) *n* (%)
Number of medications (continuous variable)	4 (1.7%) [3; 1.3%]	2 (2.3%) [2; 2.3%]	1 (5.9%) [1; 5.9%]	7 (2.0%) [6; 1.7%]
Single cut-off				
≥2 medications	1 (0.4%) [0]	10 (11.4%) [3; 3.4%]	1 (5.9%) [0]	12 (3.4%) [3; 0.8%]
≥3 medications	3 (1.3%) [1; 0.4%]	8 (9.1%) [5; 5.7%]	0	11 (3.2%) [6; 1.7%]
≥4 medications	19 (7.8%) [18; 7.4%]	23 (26.1%) [14; 15.9%]	1 (5.9%) [1; 5.9%]	43 (12.4%) [33; 13.6%]
≥5 medications	115 (47.3%) [110; 45.3%]	46 (52.3%) [34; 38.6%]	7 (41.2%) [5; 29.4%]	168 (48.3%) [149; 42.8%]
≥6 medications	25 (10.3%) [24; 9.9%]	13 (14.8%) [5; 5.7%]	2 (11.8%) [1; 5.9%]	41 (11.8%) [31; 8.9%]
≥7 medications	8 (3.3%) [8; 3.3%]	2 (2.3%) [1; 1.1%]	1 (5.9%) [1; 5.9%]	11 (3.2%) [10; 2.9%]
≥8 medications	6 (2.5%) [6; 2.5%]	1 (1.1%) [1; 1.1%]	1 (5.9%) [0]	8 (2.3%) [7; 2.0%]
≥9 medications	9 (3.7%) [8; 3.3%]	8 (9,1%) [4; 4.5%]	2 (11.8%) [2; 11.8%]	19 (5,5%) [14; 4.0%]
≥10 medications	9 (3.7%) [9; 3.7%]	8 (9.1%) [4; 4.5%]	5 (29.4%) [5; 29.4%]	22 (6.3%) [18; 5.2%]
>10 medications	0	0	3 (17.6%) [1; 5.9%]	3 (0.9%) [1; 0.3%]
≥20 medications (extreme polypharmacy)	0	1 (1.1%) [0]	2 (11.8%) [0]	3 (0.9%) [0]
Other threshold (≥13 and ≥17)	2 (0.8%) [2; 0.8%]	0	0	2 (0.5%) [2; 0.5%]
Specific threshold according to measured outcome (e.g., 3.5, 4.5, 5.5, 6.5 medications)	11 (4.5%) [11; 4.5%]	0	0	11 (3.2%) [11; 3.2%]
Range of medication (e.g., 2–9 or 4–9 medications)	1 (0.4%) [1; 0.4%]	1 (1.1%) [1; 1.1%]	0 [0]	2 (0.6%) [2; 0.6%]
Categorization				
Number of medications categorized into: 1. 0–4 medications, 5–9 medications, 10–14 medications, ≥15 medications	1 (0.4%) [1; 0.4%]	0	0	1 (0.3%) [1; 0.3%]
2. 0–4 medications, 5–8 medications, ≥9 medications	1 (0.4%) [1; 0.4%]	1 (1.1%) [0]	0	2 (0.6%) [1; 0.3%]
3. 0–5 medications, 6–8 medications, 9–11 medications, ≥12 medications	0	1 (1.1%) [1; 1.1%]	0	1 (0.3%) [1; 0.3%]
4. 5–7 medications, 8–10 medications, ≥11 medications	0	0	1 (5.9%) [1; 5.9%]	1 (0.3%) [1; 0.3%]
5. Tertiles: <4 medications, 4–6 medications, ≥7 medications	1 (0.4%) [1; 0.4%]	0	0	1 (0.3%) [1; 0.3%]
6. Quartiles: 0–6 medications, 7–9 medications, 10–13 medications, ≥14 medications	1 (0.4%) [1; 0.4%]	0	0	1 (0.3%) [1; 0.3%]
PP: ≥5 medications, EPP: ≥10 medications	18 (7.4%) [18; 7.4%]	1 (1.1%) [0]	2 (11.8%) [1; 5.9%]	21 (6.0%) [19; 5.5%]
PP: ≥6 medications, Hyper-PP: ≥10 medications	2 (0.8%) [2; 0.8%]	0		2 (0.6%) [2; 0.6%]
PP: 2–9 medications, EPP: ≥10 medications	0	1 (1.1%) [0]	0	1 (0.3%) [0]
PP: 5–9 medications, EPP or High-level PP: ≥10 medications	10 (4.1%) [10; 4.1%]	0	1 (5.9%) [1; 5.9%]	11 (3.2%) [10; 2.9%]
PP: 6–9 medications, EPP: ≥10 medications	7 (2.9%) [7; 2.9%]	3 (3.4%) [0]	0	10 (2.9%) [7; 1.0%]
PP: 5–10 medications, EPP or Extreme PP: ≥11 medications	2 (0.8%) [2; 0.8%]	0	0	2 (0.6%) [2; 0.6%]
PP: 6–10 medications, EPP: ≥11 medications	1 (0.4%) [1; 0.4%]	1 (1.1%) [1; 1.1%]	0	2 (0.6%) [2; 0.6%]
Minor PP: 2–4 medications, Major PP: ≥5 medications	3 (1.2%) [2; 0.8%]	6 (6.8%) [1; 1.1%]	1 (5.9%) [0]	10 (2.9%) [3; 0.9%]
Mild PP: 1–4 medications, Moderate PP: 5–9 medications, Severe PP: ≥10 medications	1 (0.4%) [1; 0.4%]	0	0	1 (0.3%) [1; 0.3%]
Mild PP: 2–3 medications, Moderate PP: 4–5 medications, Severe PP: ≥6 medications	1 (0.4%) [1; 0.4%]	3 (3.4%) [1; 1.1%]	0	4 (1.1%) [2; 0.6%]
Mild PP: 2–3 medications, Moderate PP: 4–6 medications, Severe PP: ≥7 medications	1 (0.4%) [1; 0.4%]	0	0	1 (0.3%) [1; 0.3%]
Mild PP: 7–9 medications, Moderate PP: 10–13 medications, Severe PP: ≥14 medications	1 (0.4%) [1; 0.4%]	0	0	1 (0.3%) [1; 0.3%]

† All the definitions mentioned in every publication were reported in the table. Since some publications provided more than one definition (for example, indicating that polypharmacy has been previously defined as five medications and more or as the use of more medications than needed), we have also collected the definition that was specifically chosen by the authors to meet the need of their study or manuscript. The information provided in square brackets thus refers to the definitions that were chosen by these authors. For example, the data should be interpreted this way: The definition “two or more medications” was cited in 10 publications in the field of clinical practice (10/88, 11%), but this definition was specifically chosen by the authors in only three publications (3/88, 3.4%). PP: Polypharmacy; EPP: Extreme polypharmacy.

**Table 2:** Numbers and proportions of publications citing qualitative and quantitative-qualitative definitions of polypharmacy in multimorbid older adults according to domain †.

Definition	Research (N = 243) *n* (%)	Clinical Practice (N = 88) *n* (%)	Public Health (N = 17) *n* (%)	TOTAL (N = 348) *n* (%)
Inappropriate medications	4 (1.6%) [4; 1.6%]	24 (27.3%) [14; 15.9%]	0	28 (8.0%) [18; 5.2%]
More medications than clinically indicated or Unnecessary medications (overuse) or presence of medications with no clinical indications or for which a safer alternative drug exists	8 (3.3%) [6; 2.5%]	34 (38.6%) [15; 17.0%]	0	42 (12.1%) [21; 6.0%]
Excessive medications	1 (0.4%) [0]	3 (3.4%) [3; 3.4%]	0	4 (1.1%) [3; 0.9%]
Drug-drug interactions or unsuitable combination of drugs	3 (1.2%) [3; 1.2%]	3 (3.4%) [1; 1.1%]	0	6 (1.7%) [4; 1.1%]
Medication prescribed to treat the side effect of another medication	0	2 (2.3%) [2; 2.3%]	0	2 (0.6%) [2; 0.6%]
Drugs that do not match the diagnosis	0	2 (2.3%) [2; 2.3%]	0	2 (0.6%) [2; 0.6%]
Duplication of medications	1 (0.4%) [1; 0.4%]	9 (10.2%) [5; 5.7%]	1 (5.9%) [1; 5.9%]	11 (3.2%) [7; 2.0%]
Drugs with lack of effectiveness	2 (0.8%) [2; 0.8%]	3 (3.4%) [1; 1.1%]	0	5 (1.4%) [3; 0.9%]
Availability of an equally effective, lower-cost alternative	1 (0.4%) [1; 0.4%]	1 (1.1%) [1; 1.1%]	0	2 (0.6%) [2; 0.6%]
Quantitative + Qualitative elements				
≥3 psychotic medications	0	0	2 (11.8%) [2; 11.8%]	2 (0.6%) [2; 0.6%]
≥5 medications and ≥1 psychotropic medication	1 (0.4%) [1; 0.4%]	0	0	1 (0.3%) [1; 0.3%]
≥5 medications, ≥2 narcotics, ≥2 benzodiazepines, ≥3 oral antidiabetic or the use of sildenafil with a nitrate	0	1 (1.1%) [1; 1.1%]	0	1 (0.3%) [1; 0.3%]
Other definitions ‡	3 (1.2%) [3; 1.2%]	16 (18.2%) [14; 15.9%]	4 (23.5%) [4; 23.5%]	23 (6.6%) [21; 6.0%]

† All the definitions mentioned in every publication were reported in the table. Since some publications provided more than one definition (for example, indicating that polypharmacy has been previously defined as 5 medications and more or as the use of more medications than needed), we have also collected the definition that was specifically chosen by the authors to meet the need of their study or manuscript. The information provided in square brackets refers to the definitions that were specifically chosen by the authors. For example, the data should be interpreted this way: the definition “Inappropriate medications” was cited in 24 publications in the field of clinical practice (24/88, 27.3%), but this definition was specifically chosen by the authors in only 14 publications (14/88, 15.9%). ‡ (e.g., Drugs with side effect, Drugs-disease interaction, Pill burden (too many to take), Complicated drug regimen affecting compliance, Taking an over the counter (OTC) medication, an herbal product or another person’s medication, Misunderstanding of the use of the medication, Dosage does not reflect age/renal/liver status, Improvement after discontinuation, Diagnosis no longer present, Contraindicated in the elderly, High-risk drugs and questionable dosing, Problematic medications, Medicines with antagonist effect, Drugs for more than one indication at the same time, Fit For The Aged (FORTA), benzodiazepine or zopiclone + opioid after hospitalization, triple whammy (Angiotensin converting enzyme inhibitor/Angiotensin receptor blocker+ diuretic + Nonsteroidal anti-inflammatory drug).

**Table 3:** Numbers and proportions of publications using or describing a quantitative definition (*n* = 335) that provided information when defining polypharmacy in multimorbid older adults according to domain.

	Research (*N* = 236) *n* (%)	Clinical Practice (*N* = 82) *n* (%)	Public Health (*N* = 17) *n* (%)	Total (*N* = 335) *n* (%)
Type of medication included				
Prescription ‡	141 (59.7)	30 (36.6)	14 (82.4)	185 (55.2)
OTC/complementary/Alternative medications §	44 (18.6)	14 (17.1)	3 (17.6)	61 (18.2)
No information reported on the type of medications included	97 (41.1)	52 (63.4)	3 (17.6)	152 (45.4)
Type of use included				
Chronic, regular or long-term drugs	53 (22.5)	12 (14.6)	9 (52.9)	74 (22.1)
As needed or short-term drugs¶	27 (11.4)	1 (1.2)	3 (17.6)	31 (9.3)
No information reported on type of use	169 (71.6)	70 (85.4)	7 (41.2)	246 (73.4)
Administration route included				
Topical, dermatologic /eye or nose drops/mouthwash††	12 (5.1)	0	2 (11.8)	14 (4.2)
Injectable agents ‡‡	4 (1.7)	0	1 (5.9)	5 (1.5)
No information reported on type of administration route	209 (88.6)	81 (98.8)	12 (70.6)	302 (90.1)
Medications-related issues				
Combination drugs counted as more than one medication	11 (4.7)	0	2 (11.8)	13 (3.9)
Method to define exposure				
Simultaneous use at a given time (simultaneous)	117 (49.6)	30 (36.6)	6 (35.3)	153 (45.7)
Simultaneous use over a period of time (continuous)	24 (10.2)	3 (3.7)	2 (11.8)	29 (8.7)
Total number of medications over a period (cumulative)	15 (6.4)	4 (4.9)	9 (52.9)	28 (8.4)
Other definitions	19 (8.1)	1 (1.2)	0	20 (6.0)
Not reported specifically	71 (30.1)	47 (57.3)	4 (23.5)	122 (36.4)

† When more than one definition is given in a publication, all the information has been included in the table, but the publication counts only once in each category. ‡ When the author stated OTC or complementary medications were excluded or included, it was considered that prescription drugs were included even if not explicitly stated. § Explicit mention that one or many types of non-prescription medicines are excluded: Research, *n* = 30; clinical practice, *n* = one; public health, *n* = four. ¶ As needed or short-term drugs were excluded in nine research and two public health publications. †† Those were excluded in 13 research and 3 public health publications. ‡‡ Those were excluded in 1 research and 1 clinical practice publications. OTC: Over the counter.

**Table 4:** Foundations provided by the authors for the choice of polypharmacy definitions among older multimorbid adults according to the field the publications pertained to.

	Research (*N* = 240) † *n* (%)	Clinical Practice (*N* = 72) † *n* (%)	Public Health (*N* = 17) *n* (%)	Total (*N* = 329) † *n* (%)
Theoretical/Methodological	28 (11.7%)	1 (1.4%)	6 (35.3%)	35 (10.6%)
Reference to previous work	108 (45%)	42 (58.3%)	7 (41.2%)	157 (47.7%)
None provided	119 (49.5%)	30 (41.7%)	5 (29.4%)	154 (46.8%)

† The evaluation was not relevant for three publications in research and 16 publications in clinical practice, as definitions were reviewed, and no choice was made. Note: Some publications used both methodological developments and references to previous work.

## Supplementary Materials

The following are available online at https://www.mdpi.com/2226-4787/7/3/126/s1, Figure S1: Characteristics of the selected studies.
