# Supplementary material for: Polypharmacy Definitions for Multimorbid Older Adults Need Stronger Foundations to Guide Research, Clinical Practice and Public Health
Source: Pharmacy (Basel). 2019 Aug 29;7(3):126. doi: 10.3390/pharmacy7030126 (PMC6789889; doi:10.3390/pharmacy7030126)
Supplement: Supplementary file 1 [file pharmacy-07-00126-s001.pdf]

**Figure S1.** Characteristics of the selected studies

| Study | Country | Design & Study period | Subjects | Setting | Sample size | Mean age (SD) [range] | Morbidities or comorbidities[1] |
|---|---|---|---|---|---|---|---|
| Abraham, Varughese, Mathew, John, and Sam (2015) | India | Retrospective cross-sectional study | ≥65 yrs | Tertiary care teaching hospital | *n* = 205<br>65.4% ♂; 34.6% ♀<br>65-74: 151<br>75-84: 39<br>85-94: 14<br>95-105: 1 | N.R. | *Nb. of comorbidities*<br>1: 27.8%<br>2: 22.9%<br>3: 14.6%<br>4: 3.4%<br>5: 1.5% |
| Advinha, de Oliveira-Martins, Mateus, Pajote, and Lopes (2014) | Portugal | Cross-sectional study<br>03/2009 to 06/2009 | ≥65 yrs taking at least one drug a day | Nursing homes | *n* = 415<br>39.8% ♂; 60.2% ♀<br>65-74: 207.1<br>75-84: 168.1<br>≥85: 39. | 83.9 (6.6) | N.R. |
| Aged Care Branch of the Department of Human Services (Victoria) (2004) | Australia | Report - Quality of Care Performance Indicator Project | Elderly | Nursing homes | N.A. | N.A. | N.A. |
| Ahmed, Nanji, Mujeeb, and Patel (2014) | Pakistan | Longitudinal cohort study<br>Follow-up: 6 weeks<br>04/2012 to 03/2013 | ≥65 yrs | Ambulatory care clinics of Tertiary Care Hospital | *n* = 1 000<br>48.5% ♂; 51.5% ♀<br>65-70: 507<br>71-80: 401<br>≥81: 92 | N. R. | N.R. |
| All Wales Medicines Strategy Group | United Kingdom | Guidance documents | Elderly | N.A. | N.A. | N.A. | N.A. |

| Study | Country | Design & Study period | Subjects | Setting | Sample size | Mean age (SD) [range] | Morbidities or comorbidities[1] |
|---|---|---|---|---|---|---|---|
| Anpalahan and Gibson (2008) | Australia | Longitudinal cohort study<br>Follow-up: 3 months | ≥75 yrs | Teaching hospital | *Non-geriatric syndrome*<br>*n* = 56<br>37.5% ♂; 62.5% ♀<br><br>*Geriatric syndrome*<br>*n* = 54<br>25.9% ♂; 74.1% ♀ | *Non-geriatric syndrome*<br>82.5 (5.7)<br><br>*Geriatric syndrome*<br>84.1 (4.1) | *Non-geriatric syndrome*<br>Heart failure 42.9%<br>COPD/pneumonia 19.6%<br>Arrhythmias 8.9%<br>Urosepsis 5.4%<br>Anaemia 3.6%<br>CIRS mean (SD): 8.4 (2.9)<br><br>*Geriatric syndrome*<br>Fall 22.2%<br>Urosepsis 18.5%<br>COPD/Pneumonia 16.7%<br>Heart failure 16.7%<br>Delirium 5.6%<br>CIRS mean (SD): 8.4 (2.5) |
| Anthierens, Tansens, Petrovic, and Christiaens (2010) | Belgium | Qualitative study | GP | Community | N.A. | N.A. | N.A. |
| Antonio, Saldana, Fernandez, and Navarro (2013) | Spain | Prospective study (poster) | ≥75 yrs with colorectal or lung cancer | N.R. | *n* = 393<br>70.5% ♂; 29.5% | 79 [75-97] | *Nb. of comorbidities*<br>≤3: 52.8%<br>>3: 47.2% |
| Akazawa, Imai, Igarashi, and Tsutani (2010) | Japan | Longitudinal cohort study<br>Follow-up: 1 yr<br>04/2006 to 09/2007 | ≥65 yrs with at least 2 pharmacy claims | Claims from hospital & Community settings | *n* = 6 628<br>28.8% ♂; 71.2% ♀<br>65-69: 2 342<br>70-74: 1 825<br>75-79: 922<br>80-84:798<br>85-89: 487<br>≥90: 254 | N.R. | *Nb. of comorbidities*<br>1: 27.8%<br>2: 22.9%<br>3: 14.6%<br>≥4: 3.4% |

| Study | Country | Design & Study period | Subjects | Setting | Sample size | Mean age (SD) [range] | Morbidities or comorbidities[1] |
|---|---|---|---|---|---|---|---|
| Azana Fernandez and Davila Barboza (2015) | Spain | Retrospective study (poster)<br>10/2014 to 11/2014 | Elderly | Hospital: Geriatric department | *n* = 100<br>28% ♂; 72% ♀ | 85.3 (5.4) | CCI mean: 2.0 |
| Azana Fernandez and Davila Barboza (2013) | Spain | Retrospective study (poster)<br>09/2012 to 10/2012 | Elderly | Hospital: Geriatric department | *n* = 112<br>36.7% ♂; 63.3% ♀ | 86.3 (6.3) | CCI mean: 2.2<br>CIRS mean (SD): 9.8 (7.1) |
| Badgwell et al. (2013) | United States | Longitudinal cohort study<br>Follow-up: 90 days<br>10/2010 to 04/2012 | ≥65 yrs, with abdominal cancer | Hospital | *n* = 111<br>55% ♂; 45% ♀ | [65-89]<br>Median: 72 | *CCI score*<br><3: 36%<br>≥3: 64% |
| Bahat et al. (2014) | Turkey | Cross-sectional study | >60 yrs, female outpatient only | Geriatrics outpatient clinics (community dwelling) | *n* = 515<br>0% ♂; 100% ♀<br>≥75: 222<br>≥80: 111 | 73.4 (6.9) [60-95] | Hypertension 75.3%<br>Depression 45.5%<br>Dementia 39.4%<br>Diabetes mellitus 27.8%<br>Hyperlipidemia 18.6%<br><br>*Nb. of diseases*<br>Mean (SD) [range]: 2.8 (1.3) [0-7] |
| Bahat et al. (2013) | Turkey | Cross-sectional study | >60 yrs, male outpatient only | Geriatrics outpatient clinics (community dwelling) | *n* = 274<br>100% ♂; 0% ♀<br>≥65: 249<br>≥75: 130<br>≥80: 66<br>≥85: 24 | 74.4 (7.1) [60-95] | Hypertension 65%<br>Depression 41.1%<br>Dementia 32.8%<br>Diabetes mellitus 24.2%<br>CVD 14.6% |

| Study | Country | Design & Study period | Subjects | Setting | Sample size | Mean age (SD) [range] | Morbidities or comorbidities[1] |
|---|---|---|---|---|---|---|---|
| Bain et al. (2008) | United States | Conceptual model | Geriatric patients | All | N.A. | N.A. | N.A. |
| Baker et al. (2014) | United Kingdom | Retrospective cohort study 01/2000 to 12/2012 | Hip fracture population | Hospital | *2000*<br>*n* = 740<br>76% ♂; 24% ♀<br><br>*2012*<br>*n* = 810<br>69% ♂; 31% ♀ | *2000*<br>Median: 81<br>IQR:75-87<br><br>*2012*<br>Median: 82<br>IQR: 75-87 | Cardiovascular disease 48%<br>Chronic obstructive airways disease 17%<br>CVD 14%<br>Diabetes mellitus 12%<br>Malignancy 12%<br><br>*Note: information about the entire study cohort (n= 10 044)* |
| Balducci, Goetz-Parten, and Steinman (2013) | United States | Narrative review | Older cancer patient | N.A. | N.A. | N.A. | N.A. |
| Ballentine (2008) | United States | Narrative review | Elderly | N.A. | N.A. | N.A. | N.A. |
| Banerjee, Mbamalu, Ebrahimi, Khan, and Chan (2011) | United Kingdom | Cross-sectional study 06/2008 to 07/2008 | ≥75 yrs | Hospital: emergency department | *n* = 467<br>43.3% ♂; 56.7% ♀ | 88 [75-101] | Fall 22%<br>Shortness of breath 12%<br>Being unwell 7.7%<br>Chest pain 5%<br>Abdominal pain 4% |

| Study | Country | Design & Study period | Subjects | Setting | Sample size | Mean age (SD) [range] | Morbidities or comorbidities[1] |
|---|---|---|---|---|---|---|---|
| Baranzini et al. (2009) | Italy | Retrospective observational study<br>07/2004 to 12/2007 | ≥65 yrs with falls | Nursing homes | *n* = 293<br>24.6% ♂; 75.4% ♀<br>65-75: 37<br>75-85: 107<br>85-95: 132<br>≥95: 17 | *Injured fallers*<br>84.6 (8.2)<br><br>*Not Injured fallers*<br>84.8 (7.7) | Cognitive impairment 72.4%<br>Depression 25.6%<br>Anxiety 19.7%<br>Bipolar mood 17.7%<br>Schizophrenic 5.4%<br><br>Mean of somatic comorbidities: 9.9 |
| Barnett and Oboh (2008) | N.R. | Expert opinion | Elderly | N.A. | N.A. | N.A. | N.A. |
| Beauplet and Martin (2015) | France | Retrospective study (abstract)<br>2013 | ≥70 yrs with multiple myeloma | Hospital | *n* = 96<br>50% ♂; 50% ♀<br>≥80: 34 | 77.7<br>Median: 76.5 | Diabetes 23.5%<br>Cardiac disease 18%<br>Vascular disease 14%<br>Respiratory disease 12.5%<br><br>*CCI score*<br>0: 41%<br>1:21%<br>2: 25%<br>≥3: 13%<br><br>CCI mean: 1.1 |
| Beer et al. (2011) | Australia | Prospective, observational cohort study<br>2001-2004<br>Follow-up: 4.5 yrs | 65-88 yrs, men only | Community | *n* = 4 260<br>100% ♂; 0% ♀ | 77 (3.6) | *No marker*<br>*CCI score*<br>1-2: 21%<br>3-4: 2.5%<br>≥5: 1.1% |

| **Study** | **Country** | **Design & Study period** | **Subjects** | **Setting** | **Sample size** | **Mean age (SD) [range]** | **Morbidities or comorbidities**[1] |
|---|---|---|---|---|---|---|---|
| Belfrage, Koldestam, Sjoberg, and Wallerstedt (2015) | Sweden | Experimental study - RCT 2009 | ≥65 yrs with hip fracture | Hospital | *n* = 200<br>33% ♂; 67% ♀ | 84.5 [65-98] | Impaired cognition 45% |
| Bell et al. (2015) | United States | Case study, expert opinion | Older cardiovascular patient | Geriatric cardiology | *Case study*<br>*n* = 1 man | *Case study*<br>81 yrs | *Nb. of chronic conditions*<br>≥2: >70%<br>≥5: 50% |
| Bell and Saraf (2014) | United States | Narrative review | >80 yrs | N.A. | N.A. | N.A. | N.A. |
| Beloosesky, Nenaydenko, Gross Nevo, Adunsky, and Weiss (2013) | Israel | Cross-sectional study 11/2011 to 02/2012 | ≥65 yrs, institutionalized for at least 1 month | Nursing homes | *n* = 993<br>28.7% ♂; 71.3% ♀<br>65-74: 103<br>75-84: 324<br>≥85: 566 | 85.4 (7.6) [65-108] | CCI mean (SD): 2.45 (1.59) |
| Bennett et al. (2014) | Australia | Prospective cohort study 05/2012 to 03/2013 | ≥60 yrs with fall | Hospital: emergency, aged care or general medicine departments | *n* = 204<br>35% ♂; 65% ♀ | 80.5 (8.3) | CCI mean (SD): 3.3 (1.8) |

| Study | Country | Design & Study period | Subjects | Setting | Sample size | Mean age (SD) [range] | Morbidities or comorbidities[1] |
|---|---|---|---|---|---|---|---|
| Best, Gnjidic, Hilmer, Naganathan, and McLachlan (2013) | Australia | Cohort study 01/2011 to 06/2011 | ≥65 yrs | Hospital: geriatric medicine or rehabilitation teams | *n* = 329<br>37.7% ♂; 62.3% ♀ | 84.6 (7) | CCI median [range]:<br>2 [0-12]<br><br>Dementia 33.4% |
| Best Practice Advocacy Centre | New Zealand | Clinical guidance review | Elderly | N.A. | N.A. | N.A. | N.A. |
| Blanco-Reina, riza-Zafra, Ocana-Riola, Leon-Ortiz, and Bellido-Estevez (2015) | Spain | Cross-sectional study | ≥65 yrs | Community-dwelling | *n* = 407<br>42.8% ♂; 57.2% ♀ | 79.3 (8) [65-100] | Hypertension 57.2%<br>Osteoarticular disease 53.3%<br>Heart disease 40%<br>Peripheral vascular 31.7%<br>Gastrointestinal 31.7%<br><br>CCI mean (SD): 1.95 (1.7) |
| Blanco-Reina, Ariza-Zafra, Gonzalez-Correa, and Leon-Ortiz (2011) | Spain | Cross-sectional study (poster) | ≥65 yrs | Public primary care centers | *n* = 407<br>42.8% ♂; 57.2% ♀ | 79.3 | N.R. |

| Study | Country | Design & Study period | Subjects | Setting | Sample size | Mean age (SD) [range] | Morbidities or comorbidities[1] |
|---|---|---|---|---|---|---|---|
| Boffin, Moreels, Vanthomme, and Van Casteren (2014) | Belgium | Nationwide cross-sectional study 01/2009 to 12/2010 | ≥65 yrs with new fall-related injuries | Community-dwelling | *n* = 1 503<br>27% ♂; 73% ♀<br>65-69: 116<br>70-74: 210<br>75-79: 317<br>80-84: 390<br>85-89: 348<br>≥90: 99 | Median: 81<br>IQR: 75-86 | N.R. |
| Bokhof and Junius-Walker (2016) | Switzerlan d | Systematic review | Elderly (≥65 yrs) and GP | Community dwelling, primary care | *n* = 14 studies | N.A. | N.A. |
| Boparai, Korc-Grodzicki, Mahmoudza deh Pournaki, and Shahrokni (2015) | United States | (abstract) 10/2009 to 12/2012 | ≥65 yrs with intra-abdominal cancer | Hospital | *n* = 592 | N.R. | N.R. |
| Boparai and Korc-Grodzicki (2011) | United States | Narrative review | Elderly | N.A. | N.A. | N.A. | N.A. |
| Bowling et al. (2014) | United States | Longitudinal study Follow-up: 2.3 yrs 01/2003 to 10/2007 | ≥75 yrs with chronic kidney disease | Community | *n* = 3557<br>44.5% ♂; 55.5% ♀ | Mean varying from79.0 to 80.6 | *Nb. of nondisease-specific problems*<br>1: 30.5% to 34.6%<br>2: 13% to 21.5%<br>3-6: 8.2% to 14% |

| Study | Country | Design & Study period | Subjects | Setting | Sample size | Mean age (SD) [range] | Morbidities or comorbidities[1] |
|---|---|---|---|---|---|---|---|
| Bradley et al. (2012) | Ireland | Cross-sectional study 07/2009 to 06/2010 | ≥70 yrs registered with GP | Primary care | *n* = 166 108<br>40.8% ♂; 59.2% ♀<br>≥75: 105 147 | N.R. | N.R. |
| Brandt (2006) | United States | Narrative review | Elderly | N.A. | N.A. | N.A. | N.A. |
| Bregnhøj, Thirstrup, Kristensen, Bjerrum, and Sonne (2009) | Denmark | Experimental study - RCT | GP & elderly (>65 yrs) on PP | Primary health care | *Combined Tx*<br>Elderly, *n* = 79<br>31.6% ♂; 68.4% ♀<br>*Single Tx*<br>Elderly, *n* = 61<br>37.7% ♂; 62.3% ♀<br>*Control group*<br>Elderly, *n* = 72<br>33.3% ♂; 66.7% ♀ | *Combined Tx*<br>Elderly: 77.1 (6.9) [65-90]<br>Median: 77<br>*Single Tx*<br>Elderly: 75.4 (7) [65-97]<br>Median: 74<br>*Control group*<br>Elderly: 77 (7.8) [65-92]<br>Median: 76 | N.R. |
| Brito, Menezes, Mesquita, and Lyra Jr (2009) | Brazil | Experimental study 01/2007 to 08/2008 | 60-75 yrs with hypertension, ≥2 drugs, ≥3 comorbidities | Primary health care unit | *n* = 30<br>10% ♂; 90% ♀<br>60-65: 10<br>65-70: 7<br>70-75: 13 | 69 (4) | Cardiovascular system 100%<br>Musculoskeletal system 90%<br>Endocrine system 83.3%<br>Nervous system 73.3%<br>Sensory organs 63.3% |

| Study | Country | Design & Study period | Subjects | Setting | Sample size | Mean age (SD) [range] | Morbidities or comorbidities[1] |
|---|---|---|---|---|---|---|---|
| Bronskill et al. (2012) | Canada | Cross-sectional study<br>09/2015 to 12/2016 | ≥66 yrs | Long-term care homes | *n* = 64 394<br>27.5% ♂; 72.5% ♀<br>66-74: 6 621<br>75-84: 24 460<br>≥85: 33 313 | ≥9 drugs: 82.6 (6.9)<br><9 drugs: 84.8 (7.5) | *Group with ≥9drugs*<br>Circulatory disease 82%<br>Endocrine & metabolic 55%<br>Musculoskeletal 54.4%<br>Mental disorders 53.6%<br>Neurological motor 37.5%<br><br>*CCI score*<br>0: 16%<br>1: 23.1%<br>≥2: 57% |
| Bushardt, Massey, Simpson, Ariail, and Simpson (2008) | United States | Literature review, conceptual model<br>01/1997 to 05/2007 | ≥65 yrs | N.A. | N.A. | N.A. | N.A. |
| Burkhardt and Wehling (2010) | Germany | Continuing education | Elderly | N.R. | N.R. | N.R. | N.R. |
| Cadogan et al. (2015) | Ireland | Qualitative study<br>05/2014 to 102/2014 | GP & community pharmacists for older people | Primary care (Health and Social Care Trust areas) | N.A. | N.A. | N.A. |
| Cahir et al. (2010) | Ireland | Longitudinal cohort study<br>Follow-up: 1yr<br>01/2007 to 12/2007 | ≥70 yrs | Community | *n* = 338 801<br>43% ♂; 57% ♀<br>70-74: 128 286<br>≥75: 210 515 | N.R. | N.R. |

| **Study** | **Country** | **Design & Study period** | **Subjects** | **Setting** | **Sample size** | **Mean age (SD) [range]** | **Morbidities or comorbidities**[1] |
|---|---|---|---|---|---|---|---|
| Cakmur (2015) | Turkey | Cross-sectional study<br>04/2014 to 09/2014 | ≥65 yrs | Data from Health Services Executive Primary Care Reimbursement Services: Community | *n* = 168<br>46.4% ♂; 53.6% ♀<br>65-79: 140<br>≥80: 28 | 72.7 (7.7) | Generalized pain 58.3%<br>Urinary incontinence 51.2%<br>Arthritis 48.3%<br>Hypertension 45.2%<br>Heart disease 17.3% |
| Canadian Institute for Health Information (2011) | Canada | Study using drug claims data from National Prescription Drugs Utilization Information System (2009) | ≥65 yrs | Community & Long-term facilities | *N.R.* | N.R. | N.R. |
| Carneiro, Azevedo-e-Silva, and Ramos-e-Silva (2011) | Brazil | Narrative review | Elderly with cutaneous adverse reactions to drugs | N.A. | N.A. | N.A. | N.A. |
| Carrasco-Garrido, Lopez De Andres, Barrera, Jimenez-Trujillo, and Jimenez-Garcia (2013) | Spain | Cross-sectional pharmacoepidemiology study<br>01/2003 to 03/2010 | ≥65 yrs | Data from National Health Surveys: Community-dwelling | *n* = 12 228 interviews<br>42.7% ♂; 57.3% ♀ | 2003: 74.3 (12.6)<br>2006: 74.7 (10.6)<br>2009: 75.2 (11.3) | Depression 54.6% to 61.3%<br><br>*Nb. of chronic conditions*<br>1-4: 14.6% to 22%<br>>4: 29.2% to 47% |

| Study | Country | Design & Study period | Subjects | Setting | Sample size | Mean age (SD) [range] | Morbidities or comorbidities[1] |
|---|---|---|---|---|---|---|---|
| Castro-Alvirena, Verdejo-Bravo, Gil Gregorio, and Lebreault Polanco (2014) | Spain | Cross-sectional study (poster) 06/2012 to 07/2012 | N.R. | Outpatient geriatric service | *n* = 108<br>24.1% ♂; 72.9% ♀ | 83.8 | CCI percentile 50$^{th}$: 2 |
| Caughey, Vitry, Gilbert, and Roughead (2009) | Australia | Retrospective cohort study (abstract) | ≥65 yrs, veterans | N.R. | *n* = 198 681 | N.R. | N.R. |
| Chan, Hao, and Wu (2009) | Taiwan | Longitudinal cohort study Follow-up: 1 yr 07/2001 to 06/2002 | ≥65 yrs | Community | *n* = 11 788<br>41.9% ♂; 58.1% ♀<br>65-74: 4 161<br>75-85: 5 104<br>≥85: 2 534 | N.R. | *Chronic conditions*<br>0-2: 45.7%<br>3-4: 35.8%<br>≥5: 13.5% |
| Chang, Chan, Kuo, Hsiung, and Chen (2011) | Taiwan | Cross-sectional study / telephone survey + evaluations | 65-79 yrs | Community-dwelling | *n* = 275<br>46.2% ♂; 53.8% ♀<br>65-68: 87<br>69-73: 112<br>74-79: 76 | 71.1 (3.8) | Memory impairment 57.5%<br>Pain 29.8%<br>Depression 16.7%<br>Urinary incontinence 14.5%<br><br>*Nb. of comorbidities*<br>Mean (SD): 3.4 (2.0) |
| C. B. Chang et al. (2011) | Taiwan | Interventional study | ≥65 yrs on PP or visited ≥3 different physicians | Database from the MSRC-Taiwan study: Hospital | *n* = 193 | 76.2 (6.2) | *Nb. of chronic conditions*<br>Mean (SD): 9 (2.6) |

| Study | Country | Design & Study period | Subjects | Setting | Sample size | Mean age (SD) [range] | Morbidities or comorbidities[1] |
|---|---|---|---|---|---|---|---|
| Chen, Kenefick, Tang, and McCorkle (2004) | United states | Narrative review<br>1980 to 2003 | Elderly with cancer | N.A. | N.A. | N.A. | N.A. |
| Cherubini et al. (2012) | Italy | Longitudinal cohort study<br>Follow-up: 1 yr<br>02/2004 | ≥65 yrs | Nursing home | *n* = 1 466<br>28.8% ♂; 71.2% ♀ | Median: 83.3<br>IQR: 77.7 | Cognitive impairment 70%<br>Severe dementia 42%<br>IHD 25.9%<br>COPD 22.9%<br>Arrhythmias 12%<br><br>CCI median: 9 |
| Chung (2014) | Korea | Narrative review | Elderly | N.A. | N.A. | N.A. | N.A. |
| Classen et al. (2007) | United States | Cross-sectional study<br>1993 to 2010 | ≥60 yrs with stroke | Home-based | *n* = 191<br>60-75: 91<br>76-85: 71<br>≥86: 29 | N.R. | Heart disease 88.0%<br>Musculoskeletal disorder 77.6%<br>Eye disease 47.9%<br>Urinary disorder 39.1%<br>Endocrine disorder 37.0%<br><br>*Nb. of comorbidities*<br>Mean (SD): 8.7 (4.7) |
| Clyne, Bradley, Hughes, Fahey, and Lapane (2012) | Ireland, United Kingdom, United States, | Systematic review | ≥65 yrs | Ambulatory care, hospital, nursing home | *Ambulatory care*<br>*n* = 6 studies<br><br>*Hospital*<br>*n* = 4 studies<br><br>*Nursing home*<br>*n* = 4 studies | N.R. | N.A. |

| Study | Country | Design & Study period | Subjects | Setting | Sample size | Mean age (SD) [range] | Morbidities or comorbidities[1] |
|---|---|---|---|---|---|---|---|
| Collerton et al. (2016) | United Kingdom | Cohort study 2006-2007 | ≥85 yrs with physical functioning difficulty | Care homes | *n* = 710<br>40.1% ♂; 59.9% ♀ | 85.5 (0.4) | Hypertension 57.8%<br>Osteoarthritis 57.0%<br>IHD 36.1%<br>Renal impairment 23.8%<br>CVD 21.1% |
| Crentsil, Ricks, Xue, and Fried (2010) | United States | Cross-sectional study<br>1992 to 1995 | ≥65 yrs with cognitive impairment | Community-Dwelling | *n* = 975<br>0% ♂; 100% ♀<br>65-74: 376.3<br>75-84:306.2<br>≥85: 292.5 | 78.3 (8.1) [65-100] | Osteoarthritis 57.5%<br>CHF 25.8%<br>Angina 25.2%<br>Diabetes mellitus 16.3%<br>COPD 16.3%<br><br>*Nb. of chronic diseases*<br>1: 22%<br>2: 29.3%<br>3: 21.4%<br>≥4: 21.7% |
| Cronin et al. (2011) | United States | Survey, experimental study<br>09/2009<br>Follow up: 2, 30 days | ≥65 yrs, undergoing major inpatient general or vascular surgery operations. | Hospital | *n* = 64 394<br>27.5% ♂; 72.5% ♀<br>66-74: 6 621<br>75-84: 24 460<br>≥85: 33 313 | 76.8 [65-92] | *≥3 comorbidities*<br>Pre-intervention: 51.4%<br>Post-intervention: 52.8% |
| Dalleur et al. (2012) | Belgium | Cohort study<br>12/2007 to 11/2008 | ≥75 yrs, positive frailty profile | Hospital | *n* = 302<br>37.4% ♂; 62.6% ♀ | Median: 84<br>IQR: 81-88 | Hypertension 55%<br>Ischemic disease 40.7%<br>Renal failure 37.4%<br>Osteoporosis with fracture 25.8%<br>Atrial fibrillation 25.5% |

| Study | Country | Design & Study period | Subjects | Setting | Sample size | Mean age (SD) [range] | Morbidities or comorbidities[1] |
|---|---|---|---|---|---|---|---|
| Davila Barboza and Azana Fernandez (2013a) | Spain | Retrospective study (congress of the EUGMS) | ≥70 yrs with hip fracture | Hospital: acute ortho-geriatric unit | *n* = 108<br>13.4% ♂; 86.6% ♀ | 87 [70-100] | CCI mean: 2.16 |
| Davila Barboza and Azana Fernandez (2013b) | Spain | Cross-sectional study (abstract) 09/2012 to 10/2012 | Elderly | Hospital: geriatric department | *n* = 112<br>36.7% ♂; 63.3% ♀ | 86.3 (6.3) | CCI mean: 2.16 |
| De Groot et al. (2016) | Netherlands | Cross-sectional study 10/2010 to 04/2013 | ≥65 yrs, outpatient, could walk safely for at least 3 minutes | Geriatric outpatient clinic | *n* = 80<br>37% ♂; 63% ♀<br>≥80 yrs: 41 | 79 (5.6) | Dementia 28%<br>Myocardial infarct 26%<br>PVD 10%<br>COPD 10%<br>CVD 6%<br><br>≥2 comorbidities: 36% |
| De Saint-Hubert, Schoevaerdts, Poulain, Cornette, and Swine (2009) | Belgium | Narrative review 1970 to 2007 | ≥65 yrs | Hospital | *n* = 21 studies | [71-85] | N.A. |
| Denneboom, Dautzenberg, Grol, and De Smet (2007) | Netherlands | Experiential study – RCT Follow-up: 6,9 months 01/2004 to 05/2004 | GP, pharmacists, elderly (≥ 75 yrs) on PP | Community pharmacy, primary care | *Elderly*<br>*n* = 738<br>37.8% ♂; 62.2% ♀ | *Elderly*<br>81 | N.R. |

| Study | Country | Design & Study period | Subjects | Setting | Sample size | Mean age (SD) [range] | Morbidities or comorbidities[1] |
|---|---|---|---|---|---|---|---|
| Denneboom, Dautzenberg, Grol, and De Smet (2006) | Netherlands | Expert opinion<br>11/2001 to 12/2002 | GP, geriatric specialists, pharmacists | 102 community-dwelling older patients on PP (≥75 yrs, ≥4 medicines) | *Elderly*<br>*n* = 102<br>38% ♂; 62% ♀ | *Elderly*<br>81 | N.A. |
| Divakaran and Murugan (2008) | United States | Letter to the editor | Elderly | N.A. | N.A. | N.A. | N.A. |
| Diez-Manglano et al. (2015) | Spain | Longitudinal cohort study<br>Follow up: 1yr<br>03/2011 to 06/2011 | Poly-pathological patient | Hospital: internal medicine departments or acute geriatric units | *n* = 457<br>45.5% ♂; 54.5% ♀ | 81 (8.8) | Heart diseases 59.1%<br>Nervous system 47.3%<br>Lung disease 34.6%<br>Autoimmune / Kidney disease: 31.5%<br>Anemia and neoplasias 27.6%<br><br>CCI mean (SD): 3.8 (2.1) |
| Diez-Manglano et al. (2014) | Spain | Cross-sectional study (letter to the Editor)<br>01/2007 to 12/2008 | Patient with chronic obstructive pulmonary disease | Hospital | *n* = 398<br>88.7% ♂; 11.3% ♀ | 73.7 (8.9) | *CCI score*<br>With PP: 2.8<br>Without PP: 2.3 |

| Study | Country | Design & Study period | Subjects | Setting | Sample size | Mean age (SD) [range] | Morbidities or comorbidities[1] |
|---|---|---|---|---|---|---|---|
| Doan, Zakrzewski Jakubiak, Roy, Turgeon, and Tannenbaum (2013) | Canada | Prospective cohort study 09/2010 to 03/2011 | ≥65 yrs on PP, newly admitted to the nonsurgical wards | Community-based hospital | *n* = 275<br>44% ♂; 56% ♀ | 83 (8.3) [65-103] | *Nb. of comorbidities*<br>Mean (SD): 6.7 (3.4) |
| Doran, Hirdes, White, Baker, Blais, Pickard, Jantzi (2009) | Canada | Secondary analysis of data collected through the Canadian Home Care reporting system, 2003-2007 | Home care clients who received an assessment | Community | 238,958 | 83% aged 65+ | Decline in physical function: 55.9%; Decline in cognition: 16.8% |
| Doupe, Brownell et al (2006) | Canada | Analysis of administrative data, 1999-2004 | Residents of personal care homes with dependency levels 1-4 | Nursing homes | 23,048 | 85+: 55.2%<br>75-84: 31.9%<br>65-74: 8.7% | 2 or more chronic diseases: 70.0%<br>Dementia: 65.3% |
| Doupe, Finlayson, Khan et al (2016) | Canada | Cohort study, April 1, 2006-Marc 31, 2011 | Long stay residents of personal care homes | Nursing homes | 5,267 | <75: 10.5%<br>75-84: 33.5%<br>85+: 56.0% | N.R. |

| Study | Country | Design & Study period | Subjects | Setting | Sample size | Mean age (SD) [range] | Morbidities or comorbidities[1] |
|---|---|---|---|---|---|---|---|
| Drenth-van Maanen, van Marum, Knol, van der Linden, and Jansen (2009) | Netherlands | Evaluation of a tool to enhance prescription quality based on the study of cases | GPs of geriatric patients | Hospital geriatric outpatient clinic (case histories) | N.A. | N.A. | *Nb. of problems treated* Mean (SD): 7.9(1.2) |
| Duerden, Avery & Payne (2013) | United Kingdom | Review document on polypharmacy | N.A. | N.A. | N.A. | N.A. | N.A. |
| Dwyer, Han, Woodwell, and Rechtsteiner (2010) | United States | Cross-sectional study 01/2004 to 12/2004 | Nursing home resident | Data from the 2004 National Nursing Home Survey | *n* = 13 403<br>28.8% ♂; 71.2% ♀<br>≤64: 1 568<br>65-74: 1 555<br>75-84: 4 208<br>≥85: 6 072 | N.R. | *Nb. of comorbidities*<br>≤3: 17.5%<br>4-6: 40.6%<br>7-9: 26.7%<br>≥10: 15.2% |
| Eckardt, Steinhagen-Thiessen, Kampfe, and Buchmann (2014) | Germany | Literature review | Elderly | N.A. | N.A. | N.A. | N.A. |
| Egan, O'Connor, Conde, Fitzgerald, and O'Toole (2012) | Ireland | Cross-sectional study (Abstract) | Elderly | Long-term care home | *n* = 115 | [66-102] | N.R. |

| Study | Country | Design & Study period | Subjects | Setting | Sample size | Mean age (SD) [range] | Morbidities or comorbidities[1] |
|---|---|---|---|---|---|---|---|
| Eijsink, Zeeman, Van Wijngaarden, Badings, and Van'T Riet (2015) | Netherlands | Longitudinal retrospective cohort study (abstract) | ≥65 yrs with heart failure | Hospital | *n* = 114 | N.R. | N.R. |
| Elliot et al. (2014) | United States | Longitudinal cohort study Follow-up: 60 days 01/2004 to 12/2009 | ≥60 yrs treated for acute myelogenous leukemia | Hospital | *n* = 150<br>61% ♂; 39% ♀ | [61-87]<br>Median: 69 | CAD 17%<br>Diabetes 19%<br><br>CCI score ≥1: 45%<br><br>*Nb. of comorbidities*<br>Median [range]: 3 [0-10] |
| Ervin (2014) | Australia | Expert opinion (letter to the editor) | Elderly | N.A. | N.A. | N.A. | N.A. |
| Espino et al. (2006) | United States | Longitudinal cohort study Follow-up: 8 yrs 0/1993 to N.R. | 65-99 yrs, Mexican-American | Community-based population | *n* = 3 050<br>42.4% ♂; 57.6% ♀ | 73 | Hypertension 41.9%<br>Diabete 23.7%<br>CVD 9.3%<br>Stroke 6.3%<br>Neoplasms 5.3% |
| Farrell, Shamji, Monahan, and French Merkley (2013) | Canada | Expert opinion | Elderly | Geriatric Day Hospital | N.A. | N.A. | N.A. |

| Study | Country | Design & Study period | Subjects | Setting | Sample size | Mean age (SD) [range] | Morbidities or comorbidities[1] |
|---|---|---|---|---|---|---|---|
| Fastbom & Johnell (2015) | Sweden | Description of National indicators of quality of drug therapy | People aged 75 years and over | All settings | N.A. | N.A. | N.A. |
| Fialova and Onder (2009) | Italy, Czech Republic | Narrative review | Elderly | N.A. | N.A. | N.A. | N.A. |
| Finkelstein, Friedman, Hripcsak, and Cabrera (2016) | United States | Case-control study 01/2011 to 12/2015 | ≥65 yrs with PP and CVD | Hospital: acute and outpatient care | *n* = 12<br>67% ♂; 33% ♀ | 77 (5) [69-83] | *Chronic diseases*<br>mean (SD): 7.8 (1.2)<br><br>*Geriatric syndromes*<br>mean (SD): 4.0 (1.7) |
| Flood, Carroll, Le, and Brown (2009) | United states | Prospective study 12/2005 to 02/2006 | ≥65 yrs or <65 yrs if referred by nursing staff, with cancer | Hospital: oncology-acute care for elders' unit | *n* = 47<br>40% ♂; 60% ♀ | 73.5 (7.5) [60-94] | N.R. |
| Flood, Rohlfing, Le, Carr, and Rich (2007) | United States | Prospective cohort study 01/2003 to 12/2003 | ≥70 yrs with cardiovascular disease | Hospital | *n* = 100<br>39% ♂; 61% ♀ | 79.2 (5.5) | Hypertension 83%<br>CAD 67%<br>Hyperlipidemia 53%<br>Atrial fibrillation 50%<br>Heart failure 46% |
| Franchi et al. (2016) | Italy | Longitudinal cohort study Follow-up: 2 yrs 01/2001 to 12/2010 | 65-95 yrs | Community-dwelling | *2001*<br>*n* = 1 567 575<br>39.7% ♂; 60.3% ♀<br>65-74: 917 693<br>75-84: 495 722<br>≥85: 154 110<br><br>*2009* | N.R. | N.R. |

| Study | Country | Design & Study period | Subjects | Setting | Sample size | Mean age (SD) [range] | Morbidities or comorbidities[1] |
|---|---|---|---|---|---|---|---|
| | | | | | *n* = 1 800 257<br>41.7% ♂; 58.3% ♀<br>65-74: 998 898<br>75-84: 623 121<br>≥85: 178 238 | | |
| Franchi et al. (2013) | Italy | Cohort study 01/2000 to 12/2010 | 65-95 yrs | Community | *2000*<br>*n* = 1 554 004<br><br>*2005*<br>*n* = 1 767 428<br><br>*2010*<br>*n* = 1 917 646 | N.R. | N.R. |
| Franchini, Pieroni, Fortunato, Molinaro, and Liebman (2014) | Italy | Social Network Analysis 01/2010 to 12/2010 | ≥75 yrs with type 2 diabetes | Health care structures | *n* = 1 976<br>42.8% ♂; 57.2% ♀<br>75-79: 783<br>80-84: 614<br>≥85: 579 | N.R. | Diabetes 100%<br>CVD 95.1%<br>Hypertension 73.2%<br>DSD 47.1%<br>Endrocrine, nutritional & metabolic disease 38.8%<br><br>*Nb. of comorbidities*<br>1: 17.8%<br>2: 32.6%<br>3: 26.6%<br>4: 15.7%<br>≥5: 5.0% |
| Frank (2014) | Canada | Editorial | Elderly | N.A. | N.A. | N.A. | N.A. |

| Study | Country | Design & Study period | Subjects | Setting | Sample size | Mean age (SD) [range] | Morbidities or comorbidities[1] |
|---|---|---|---|---|---|---|---|
| Frazier (2005) | United States | Systematic review<br>1995 to 2003 | Elderly | Community, hospital, home care | *n* = 16 studies | N.R. | N.A. |
| Fried et al. (2014) | United States | Systematic review<br>1946 to 05/2014 | ≥65 yrs | Community | *n* = 58 studies | N.R. | N.A. |
| Frohnhofen, Michalek, and Wehling (2011) | Germany | Pilot study, before-after study | Geriatric | Hospital | *n* = 46<br>26% ♂; 74% ♀ | 84 (6) | Dementia 48%<br>Musculoskeletal disease 22%<br>CVD 15%<br>Pulmonary disease 9% |
| Frutos Bernal, Martín Corral, and Galindo Villardón (2011) | Spain | Survey<br>2006 | ≥65 yrs, non-institutionalised | Community | *n* = 458<br>35.8% ♂; 64.2% | 75.3 [65-101] | Chronic illness 93.9%<br>Arthrosis, arthritis or rheumatism 53.5%<br>Hypertension 48.3% |
| Fulton and Allen (2005) | United States | Systematic review<br>01/1991 to 10/2003 | ≥60 yrs | Primary care | *n* = 16 studies | N.R. | N.A. |
| Fustinoni, Renard, Santos-Eggimann, and Seematter-Bagnoud (2015) | Switzerland | Survey nested into a population-based cohort (71% participation) | ≥69 yrs | Community | *n* = 3 133<br>73% ♂; 27% ♀ | 77 | N.R. |

| Study | Country | Design & Study period | Subjects | Setting | Sample size | Mean age (SD) [range] | Morbidities or comorbidities[1] |
|---|---|---|---|---|---|---|---|
| Galazzi et al. (2016) | Australia, Italy | Survey 2015 | ≥65 yrs on PP | Hospital: geriatric and internal medicine wards | *n* = 100<br>53% ♂; 47% ♀<br>65-74: 36<br>75-84: 46<br>≥85: 18 | [65-93]<br>Median: 79<br>IQR: 73-83 | Cardiovascular 87%<br>Metabolic 40%<br>Osteoarticular 40%<br>Respiratory 33%<br>Renal 30%<br><br>*Nb. of comorbidities*<br>Median (IQR): 4 (4-5) |
| Gallagher et al. (2011) | Switzerland, Spain, Belgium, Italy, Czech Republic, Ireland | Cross-sectional prospective study<br>08/2008 to 12/2008 | ≥65 yrs with acute illness | Hospital: acute geriatric medicine units | *n* = 900<br>39% ♂; 61% ♀<br>65-74: 174<br>75-84: 420<br>≥85: 333 | Median: 82<br>IQR: 77-87 | Dementia 29%<br>CVD 29%<br>IHD 22%<br>Cardiac failure 18%<br>Diabetes mellitus 18%<br><br>*CCI score*<br>0: 12%<br>1:21%<br>≥2: 66% |
| Gamble et al. (2014) | Canada | Longitudinal cohort study and methodological study<br>Follow-up: 90 days, 1 yr<br>2000 to 2002 | ≥65 yrs with community-acquired pneumonia, survived at least 1 yr | Hospital and emergency departments | *n* = 2 105<br>50% ♂; 50% ♀<br>65-74: 784<br>75-84: 886<br>≥85: 435 | 78 (8) | Heart disease 51%<br>COPD 30%<br>CVD 13%<br>Mental illness 13%<br>Neoplasm 12% |

| Study | Country | Design & Study period | Subjects | Setting | Sample size | Mean age (SD) [range] | Morbidities or comorbidities[1] |
|---|---|---|---|---|---|---|---|
| Garcia-Baztan et al. (2014) | Spain | Retrospective study 01/2009 to 11/2010 | Elderly | Acute general hospital, convalescence geriatric unit, community | *n* = 334<br>39.5% ♂; 60.5% ♀ | 77.9 (11.2) | Osteoarticular fracture 28.3% to 55.1%<br>Neurological 13.2% to 29.3%<br>Circularoty 5.8% to 8.5%<br>Cardiology 3.5% to 3.8%<br>Respiratory 3.2% to 7.4%<br><br>CCI mean (SD): 1.8 to 2.3 (1.6 to 1.7)<br>* According to the group with/without BZD |
| Garfinkel, Ilhan, and Bahat (2015) | Israel, Turkey | Narrative review, Expert opinion | Elderly | N.A. | N.A. | N.A. | N.A. |
| Garfinkel, Zur-Gil, and Ben-Israel (2007) | Israel | Experimental study 2004 to 2005 Follow up: 1 yr | Elderly | Nursing departments | *n* = 190<br>31.1% ♂; 68.9% ♀ | *Study group (GPA)*<br>81.2 (8.3)<br><br>*Control group*<br>(82 (8.7) | Double incontinence 93%<br>Hypertension 41% to 46%<br>Previous stroke 39%<br>Diabetes mellitus 24% to 30%<br>Recurrent infections 29%<br>* According to the study group (GPA) or control group |

| Study | Country | Design & Study period | Subjects | Setting | Sample size | Mean age (SD) [range] | Morbidities or comorbidities[1] |
|---|---|---|---|---|---|---|---|
| Garrido-Garrido et al. (2011) | Spain | Cross-sectional study 06/2009 to 07/2009 | ≥65 yrs with PP | Primary care center | *n* = 305<br>34.4% ♂; 65.6% ♀<br>64-74: 131<br>75-84: 134<br>≥85: 40 | N.R. | Hypertension 78%<br>Arthrosis 73%<br>Dyslipemia 45.5%<br>Osteoporosis 45.5%<br>Anxiety 38.5%<br><br>Average nb. of diseases: 5.56 |
| Gentes, Hertzog, Vogel, and Lang (2015) | France | Cross-sectional study 01/2012 to 04/2012 | ≥65 yrs, ≥ 2 comorbidities | Day-care hospital, short or rehabilitation ward, nursing home | *n* = 100<br>40% ♂; 60% ♀<br>65-74: 11<br>75-80: 19<br>≥81: 70 | *N.R.* | Atherosclerosis 69%<br>Hypertension 52%<br>Type 2 diabetes 25%<br>Falls (last 3 months) 24%<br>Depression 19% |
| Girones, Torregrosa, and Diaz-Beveridge (2010) | Spain | Cross-sectional study 01/2005 to 06/2006 | ≥70 yrs, female outpatient (breast cancer survivors) | Outpatient clinics of the Medical Oncology unit | *n* = 91<br>0% ♂; 100% ♀ | *At diagnosis*<br>76 [70-92]<br><br>*At interview*<br>80 [71-95] | Hypertension 34%<br>PVD 26%<br>Diabetes 13%<br>Heart failure 8%<br>Coronary syndrome 7%<br><br>CCI score ≥2: 61% |
| Gnjidic et al. (2015) | Australia | Longitudinal cohort study Follow-up: 1 yr | ≥70 yrs, ambulant | Residential aged care facilities | *n* = 540<br>27.6% ♂; 72.4% ♀<br>70-79: 100<br>80-89: 288<br>≥90: 152 | 85.5 (6.4) | CCI mean (SD): 2.5 (1.7) |
| Gnjidic, Hilmer, Blyth, Naganathan, Cumming, et al. (2012) | Australia | Longitudinal cohort study Follow-up: 2 yrs | ≥70 yrs, male only | Community-dwelling | *n* = 1 662<br>100% ♂; 0% ♀ | 76.9 (5.4) | *Nb. of comorbidities*<br>Mean (SD): 1.9 (1.6) |

| Study | Country | Design & Study period | Subjects | Setting | Sample size | Mean age (SD) [range] | Morbidities or comorbidities[1] |
|---|---|---|---|---|---|---|---|
| Gnjidic, Hilmer, Blyth, Naganathan, Waite, et al. (2012) | Australia | Methodology study<br><br>01/2005 to 05/2011 | ≥70 yrs, male only | Community-dwelling | *n* = 1 705<br>100% ♂; 0% ♀ | 76.9 (5.5) [70-97] | Dementia or MCI 12.5%<br><br>*Nb. of comorbidities*<br>Mean (SD): 1.8 (1.5) |
| Gomez et al. (2015) | Spain | Cohort study<br>Follow-up: 13 yrs<br>01/1994 to 05/2007 | ≥65 yrs | Community | *n* = 5 052<br>42.2% ♂; 57.8% ♀ | 74.4 (6.9) | *Comorbidity index [2], mean (SD) [median]*<br>0 drug: 0.6 (1.2) [0]<br>1-5 drugs: 1.2 (1.5) [1]<br>≥6 drugs: 2.4 (1.9) [2] |
| Gomm et al. (2016) | Germany | Cohort study<br>2004-2011 | ≥75 yrs, inpatient and outpatient without dementia at baseline | Data from Germany statutory health insurer | *n* = 73 679<br>22.1% ♂; 77.9% ♀ | 83.58 (5.4) | ≥*2* different type of dementia: 59%<br><br>*With Dementia*<br>IHD 40.7%<br>Diabetes 32.9%<br>Depression 17%<br>Stroke 4.5% |
| Greene, Steinman, McNicholl, and Valcour (2014) | United States | Cohort study (retrospective chart review)<br>2008 to 2011 | >60 yrs with or without immuno-deficiency virus infection (HIV) | Community-dwelling | *n* = 117<br>92.3% ♂; 7.7% ♀ | *Positive HIV*<br>Median: 63<br>IQR: 60-82<br><br>*Negative HIV*<br>Median: 65<br>IQR: N.R. | Hyperlipidemia 61%<br>Cognitive disorder 46%<br>Hypertension 39% to 43%<br>Depression 18% to 37%<br>Diabetes mellitus 11% |
| Haefeli (2014) | Germany | Literature review (abstract) | Elderly | N.A. | N.A. | N.A. | N.A. |

| Study | Country | Design & Study period | Subjects | Setting | Sample size | Mean age (SD) [range] | Morbidities or comorbidities[1] |
|---|---|---|---|---|---|---|---|
| Haider, Johnell, Weitoft, Thorslund, and Fastbom (2009) | Sweden | Cross-sectional study<br>1998-10/2005 | 75-89 yrs filled at least one drug prescription | Data from National registers | *n* = 626 258<br>39.7% ♂; 60.3% ♀<br>77-79: 258 836<br>80-84: 226 105<br>≥85: 141 317 | 80.9 | CVD 10%<br>Myocardial infraction 6%<br>Cancer 6%<br>CAD 4% |
| Haider, Johnell, Thorslund, and Fastbom (2008) | Sweden | Cross-sectional study<br>2002 | ≥77 yrs | Data from SWEOLD: Community, group living for the demented, long-term care facilities | *n* = 621<br>40.7% ♂; 59.3% ♀<br>77-79: 158<br>80-84: 243<br>≥85: 220 | 83.3 | ≥*2* comorbidities: 50.9% |
| Hajjar, Cafiero, and Hanlon (2007) | Unites States | Literature review<br>1986 to 06/2007 | >65 yrs | N.R. | *n* = 5 to 21 studies according to the objective | N.R. | N.A. |
| Halling, Fridh, and Ovhed (2006) | Sweden | Cohort study<br>04/2001 to 05/2003 | ≥60 yrs | Community | *n* = 1 144<br>41.7% ♂; 58.3% ♀ | [60-96] | N.R. |
| Hamaker et al. (2014) | Netherland s | Experimental study<br>Median follow-up: 32 months<br>04/2007 to 09/2011 | ≥65 yrs, female only with metastatic breast cancer | Hospital | *n* = 78<br>0% ♂; 100% ♀<br>65-69: 17<br>70-74: 16<br>75-80: 30<br>≥81: 10 | 75.5 [66-87] | Mild depression 29%<br>Severe depression 4%<br><br>*CCI score*<br>0-1: 88%<br>≥2: 12% |
| Haque (2009) | United States | Conceptual model | Elderly | N.A. | N.A. | N.A. | N.A. |

| Study | Country | Design & Study period | Subjects | Setting | Sample size | Mean age (SD) [range] | Morbidities or comorbidities[1] |
|---|---|---|---|---|---|---|---|
| Harugeri, Joseph, Parthasarathi, Ramesh, and Guido (2010a) | India | Prospective observational study 01/2008 to 06/2009 | ≥60 yrs with at least one medication at admission or during hospital stay | Hospital | *n* = 814<br>60.9% ♂; 39.4% ♀<br>60-64: 234<br>65-69: 254<br>70-74: 144<br>75-79: 111<br>≥80: 71 | [60-95]<br>Median: 66 | Hypertension 41.5%<br>Diabetes mellitus 34%<br>COPD 18.5%<br><br>*Nb. of diseases*<br>1: 19%<br>2: 32.6%<br>3: 26%<br>≥4: 22.4% |
| Harugeri, Joseph, Parthasarathi, Ramesh, and Guido (2010b) | India | Prospective study 01/2008 to 06/2009 | 60-95 yrs | Tertiary care hospital | *n* = 814<br>60.6% ♂; 39.4% ♀<br>60-69: 488<br>70-79:255<br>≥80: 71 | [60-95]<br>Median: 66 | Hypertension 42.4%<br>Diabetes mellitus 35.7%<br>COPD 18.8%<br>Angina pectoris 9.2%<br><br>*Nb. of diagnoses*<br>1-2: 51.6%<br>3-4: 41.5%<br>≥5: 6.9% |
| Health Quality & Safety Commission (2017) | New-Zealand | Methodology paper for Polypharmacy Atlas | 65 years and over | All | N.A. | N.A. | N.A. |

| Study | Country | Design & Study period | Subjects | Setting | Sample size | Mean age (SD) [range] | Morbidities or comorbidities[1] |
|---|---|---|---|---|---|---|---|
| Heppner et al. (2012) | Germany | Descriptive cohort<br>2006 to 2009 | ≥70 yrs | Hospital: Clinical geriatrics, Poison information center Nuremberg, Toxicological intensive care unit | *n* = 292<br>70-80: 173<br>≥81: 119 | N.R. | N.R. |
| Hernandez Perella et al. (2013) | Spain | Cross-sectional study<br>2010 | ≥65 yrs with active clinical history | Urban clinics | *n* = 363<br>43.3% ♂; 56.7% ♀ | 75.2 (7.0) | CVD 76.9%<br>Osteoarticular 57.6%<br>Urogenital 31.4% |
| Hernandez, Ceballos, Martin, and Salazar (2011) | Spain | Cross-sectional analysis of cohort (Abstract - congress of the EUGMS)<br>2010 | Elderly | Hospital: acute geriatric unit | *n* = 150<br>38% ♂; 62% ♀ | 87 (5.8) | Cause of hospitalisation:<br>Infectious disease 51.3%<br>Heart failure 16%<br>Stroke 4.66%<br>Electrolyte disturbances 4%<br><br>CCI score ≥4: 81.3% |
| Hilmer, Gnjidic, and Le Couteur (2012) | Australia | Narrative review | Elderly | Primary care | N.A. | N.R. | N.A. |
| Hilmer and Gnjidic (2009) | Australia | Narrative review | Elderly | N.A. | N.A. | N.A. | N.A. |
| Hofer-Duckelmann (2012) | Germany | Book chapter | Elderly | N.A. | N.A. | N.A. | N.A. |

| Study | Country | Design & Study period | Subjects | Setting | Sample size | Mean age (SD) [range] | Morbidities or comorbidities[1] |
|---|---|---|---|---|---|---|---|
| Hoffmann et al. (2011) | Germany | Cohort study<br>03/2005 to 03/2006 | Outpatient with a first diagnosis of dementia | Outpatient care | *n* = 1 848<br>52.4% ♂; 47.6% ♀<br>64-74: 566<br>75-84: 870<br>≥ 84: 412 | 78.7 (7.4) | Disturbance, hearing loss 47.7%<br>Pain 46.1%<br>High risk of complication 35.9%<br>Depression, anxiety 32.9%<br>Incontinence 24.0% |
| Hofmann and Hach (2011) | Germany | Descriptive cross-sectional study (abstract) | 1) ≥65 yrs inpatients; 2) new patients of the department of traumatology and orthopedy | Hospital | *Inpatients*<br>*n* = 677<br><br>*New patients*<br>*n* = 390<br>(≥ 65 yrs: *n* = 121) | *Inpatients:* N.R.<br><br>*New patients ≥ 65 yrs:* 72.5 | N.R. |
| Hopcroft et al. (2014) | Australia | Cohort study<br>07/2005 to 05/2010 | ≥70 yrs, minimum hospital stay of 48 h | Acute care hospital | *n* = 206<br>31.1% ♂; 68.9% ♀ | 84.8 (6.8) | *Nb. of comorbidities*<br>Mean (SD): 6 (2.2) |
| Hovstadius, Petersson, Hellström, and Ericson (2014) | Sweden | Population-based cohort study<br>2006-2013 | ≥65 yrs | Entire population | *2013*<br>*n* = 1 828 283<br>65-69: 595 931<br>70-74: 422 593<br>75-79: 311 611<br>80-84: 245 180<br>85-89: 161 736<br>90-94: 73 675<br>95-99: 15 721<br>≥100: 1 836 | N.R. | N.R. |

| Study | Country | Design & Study period | Subjects | Setting | Sample size | Mean age (SD) [range] | Morbidities or comorbidities[1] |
|---|---|---|---|---|---|---|---|
| Hovstadius and Petersson (2012) | Sweden | Narrative review | Elderly | N.A. | N.A. | N.A. | N.A. |
| Hovstadius, Astrand, and Petersson (2010) | Sweden | Cohort study 07/2006 to 09/2006 | Entire Swedish population<br><br>Information collected for ≥70 yrs | Community Data from: Swedish Prescribed Drugs Register | *n* = 2 287 428<br>41.8% ♂; 58.2% ♀ | N.R. | N.R. |
| Hovstadius, Hovstadius, Astrand, and Petersson (2010) | Sweden | Cohort study 07/2005 to 09/2008 | Entire Swedish population<br><br>Information collected for ≥60 yrs | Community, Data from: Swedish Prescribed Drugs Register | 2005: 9 029 750<br>2006: 9 080 505<br>2007: 9 146 092<br>2008: 9 219 637 | N.R. | N.R. |
| Hubbard et al. (2015) | Australia | Longitudinal cohort 06/2005 to 05/2010 | ≥70 yrs, admitted to general medical units with expected hospital stay > 48 hours | Acute care hospital | *n* = 1 216 | 81.3 (6.8) | Circulatory system: 21.4%<br>Respiratory system: 20.9%<br>Nervous systems: 10.3%<br>Kidney / urinary tract: 7.9% |

| Study | Country | Design & Study period | Subjects | Setting | Sample size | Mean age (SD) [range] | Morbidities or comorbidities[1] |
|---|---|---|---|---|---|---|---|
| Hudhra et al. (2016) | Spain | Cross-sectional cohort study 04/2011 to 06/2012 | ≥75 yrs | Hospital | *n* = 624<br>55% ♂; 45% ♀ | 77.7 | CCI mean: 3.2 |
| Huiart et al. (2013) | France | Cohort study 10/2006 to 06/2011 | ≥65 yrs, women with breast cancer | Community | *n* = 382<br>0% ♂; 100% ♀<br>65-74: 223 | 71.8 (4.2) | Comorbidities: 50.6%<br>Depression 11.2% |
| Hurria et al. (2012) | N.R. | Clinical practice guidelines | Elderly with cancer | N.A. | N.A. | N.A. | N.A. |
| Inventor et al. (2005) | United States | Expert opinion | Elderly, inpatient with both medical and psychiatric illness | Geriatric psychiatry unit | N.A. | N.A. | N.A. |
| Jensen, Andersen, Hallin, and Petersen (2014) | Denmark | Longitudinal observational study<br>Follow-up: 30 days<br>10/2011 to 12/2011 | ≥65 yrs | Hospital: acute medical unit | *n* = 71<br>54.9% ♂; 45.1% ♀ | 78.7 [65.3-100.9] | *CCI score*<br>0: 52.1%<br>1: 35.2%<br>≥2: 12.7% |

| Study | Country | Design & Study period | Subjects | Setting | Sample size | Mean age (SD) [range] | Morbidities or comorbidities[1] |
|---|---|---|---|---|---|---|---|
| Jimenez Paez, Ariza Fuentes, Cardona Pelaez, Monterde Perez, and Salas Becerra (2011) | Spain | Retrospective study (abstract) 2010-2012 | Elderly | Residential environment | *n* = 313<br>28.4% ♂; 71.6% ♀ | 82 | Dementia 66.7%<br>Hypertension 65.5%<br>Articular pathology 53.7%<br>Diabetes mellitus 28.7%<br>Cardiac disease 18.0% |
| Jorgensen, Herrstedt, Friis, and Hallas (2012) | Denmark | Case-control study 1996-2006 | Person with or without cancer | Data from Danish cancer registry and Odense pharmaco-epidemiologic: Community | *n* = 124 037<br>48.3% ♂; 51.7% ♀<br>0-49: 14 874<br>50-59: 20 550<br>60-69: 30 440<br>70-79: 34 220<br>80-89: 20 905<br>≥90: 3048 | Median: 68<br>IQR: 58-77 | N.R. |
| Jorgensen, Hallas, and Herrstedt (2010) | Denmark | Case-control study (abstract) 1996-2006 | Elderly with cancer | Community | *n* = 150 607<br>*Cancer cases*<br>≥70: 12 832<br><br>*Controls*<br>≥70: 51 298 | N.R. | N.R. |
| Jorgensen, Hallas, Land, and Herrstedt (2010) | Denmark | Narrative review | Elderly with cancer | Community | N.A. | [63-101]<br>Median:<br>61 to 75 yrs | N.A. |

| Study | Country | Design & Study period | Subjects | Setting | Sample size | Mean age (SD) [range] | Morbidities or comorbidities[1] |
|---|---|---|---|---|---|---|---|
| Junius-Walker, Theile, and Hummers-Pradier (2007) | Germany | Cohort study | ≥70 yrs | Primary care | *n* = 466<br>28.5% ♂; 71.5% ♀ | ♂: 77<br>♀: 76.3 | Incontinence 61.3%<br>Anxiety 52.6%<br>High cholesterol 49.4%<br>Hypertension 37.2%<br>Diabetes 28.9% |
| Jyrkkä, Enlund, Lavikainen, Sulkava, and Hartikainen (2011) | Finland | Longitudinal prospective cohort study<br>Follow-up: 3 yrs<br>11/2003 to 2007 | ≥75 yrs | Population based | *n* = 294<br>31% ♂; 69% ♀ | 2004: 81.4<br>2007: 84.1 | *FCI, mean*<br>2004: 2.7<br>2007: 2.7 |
| Jyrkka, Enlund, Korhonen, Sulkava, and Hartikainen (2009) | Finland | Longitudinal population-based cohort study<br>Follow-up: 4 yrs<br>1998-2007 | ≥75 yrs | Community | *n* = 332<br>25.1% ♂; 74.9% ♀<br>80-84: 171<br>≥85: 168 | 84.9 (3.5) | N.R. |
| Jyrkkä, Enlund, Korhonen, Sulkava, and Hartikainen (2009) | Finland | Cross-sectional analysis of a cohort study<br>01/1998 | ≥75 yrs | Population-based | *n* = 523<br>26% ♂; 73% ♀<br>80-84: 171<br>≥85: 168 | 81 [75-93] | Hypertension 63.7%<br>Heart disease 62.7%<br>Pain 54.5%<br>Depression 22.0%<br>Diabetes 19.7% |
| Jyrkka, Vartiainen, Hartikainen, Sulkava, and Enlund (2006) | Finland | Prospective longitudinal cohort study<br>Follow up: 5 yrs<br>01/1998 to 12/2003 | ≥75 yrs | At home, in institution | *n* = 339<br>25.1% ♂; 74.9% ♀<br>70-79: 171<br>80-84: 116<br>≥85: 52 | 80 | N.R. |

| Study | Country | Design & Study period | Subjects | Setting | Sample size | Mean age (SD) [range] | Morbidities or comorbidities[1] |
|---|---|---|---|---|---|---|---|
| Kenig, Olszewska, Zychiewicz, Barczynski, and Mitus-Kenig (2015) | Poland | Methodology study<br>Follow up: 30 days<br>06/2013 to 05/2014 | ≥65 yrs qualified for abdominal surgery due to solid cancer | Tertiary referral hospital | *n* = 75<br>56% ♂; 44% ♀<br>65-74: 48<br>75-84: 25<br>≥85: 2 | 73 (5.8) | CCI score ≥3: 71% |
| Klarin, Wimo, and Fastbom (2005) | Sweden | Population-based study longitudinal<br>Follow-up: 3 yrs<br>09/1995 to 06/1998 | ≥75 yrs | Community | *n* = 785<br>42% ♂; 58% ♀<br>75-79: 263<br>80-84: 307<br>≥85: 215 | 82.2 | *CCI score*<br>0: 28.9%<br>1: 27.1%<br>2: 22.8%<br>≥3: 21.2% |
| G. Kojima et al. (2012) | United States | Experimental study – pre-post treatment<br>10/2007 | Residents with PP | Hospital-affiliated long term care facility | *n* = 70<br>27.1% ♂; 72.9% ♀ | 82.7 [47-104] | N.R. |
| T. Kojima et al. (2012) | Japan | Longitudinal observational study, methodological study<br>Follow-up: 2 yrs<br>2006 to 2007 | ≥65 yrs, outpatient receiving treatment for chronic disease | Clinic of the research institute of aging science | *n* = 172<br>26.2% ♂; 73.3% ♀ | 77 (7) | Hypertension 62%<br>Dyslipidemia 44%<br>Osteoporosis 34%<br>Diabetes 13%<br>History of cancer 5% |
| Kristjansson et al. (2010) | Norway | Longitudinal cohort study<br>Median follow-up: 20 months<br>11/2006 to 06/2008 | ≥70 yrs with confirmed or suspected colorectal cancer | Hospital | *n* = 182<br>43% ♂; 57% ♀<br>70-74: 43<br>75-79: 47<br>80-84: 58<br>85-89: 24<br>≥90: 10 | [70-94]<br>Median: 80 | Depression 11%<br><br>*CIRS score*[3]<br>Mild comorbidity: 26%<br>Moderate comorbidity: 52%<br>Severe comorbidity: 23% |

| Study | Country | Design & Study period | Subjects | Setting | Sample size | Mean age (SD) [range] | Morbidities or comorbidities[1] |
|---|---|---|---|---|---|---|---|
| Kronfly Rubiano et al. (2015) | Spain | Cross-sectional study<br>06/2009 to 07/2010 | ≥75 yrs | Primary health care | *n* = 290<br>35.1% ♂; 64.9% ♀ | 79.4 (3.2) | *Comorbidities most common among depressed elderly*<br>Thyroid disease 58.8%<br>Cancer 57.1%<br>Valvulopathies 52.9%<br>Hypertrophic cardiomyopathy 50.0%<br>AVD 48.6% |
| Kuhn-Thiel, Weis, Wehling, and members (2014) | Germany, Austria | Expert opinion, survey | Geriatric internists / geriatricians, geriatric psychiatrists | N.R. | *n* = 20 | N.R. | N.A. |
| Kuijpers, van Marum, Egberts, and Jansen (2008) | Netherlands | Review of medical and pharmacy records<br>09/2004 to 02/2005 | Geriatric population | Department of geriatric medicine of the University Medical Centre Utrecht | *n* = 150<br>36% ♂; 64% ♀ | 79.6 (7.3) [65-100] | N.R. |
| Lai et al. (2012) | Taiwan | Case-control study<br>2000 to 2008 | ≥65 yrs with or without newly dementia | Outpatient clinics | *n* = 35 675<br>48% ♂; 52% ♀<br>65-74: 15 018<br>75-84: 16 900<br>≥85: 3 757 | *No dementia*<br>76.5 (5.7)<br><br>*Dementia*<br>78.9 (6.8) | Hypertension 69.1%<br>CVD 41.1%<br>Hyperlipidemia 29.3%<br>Diabetes mellitus 26.8%<br>CKD 8.2% |

| Study | Country | Design & Study period | Subjects | Setting | Sample size | Mean age (SD) [range] | Morbidities or comorbidities[1] |
|---|---|---|---|---|---|---|---|
| Lai et al. (2011) | Taiwan | Case-control study<br>2000 to 2008 | ≥65 yrs with or without Parkinson disease | *Parkinson*<br>Ambulatory care<br><br>*No Parkinson*<br>National health insurance dataset | *n* = 14 135<br>51.8% ♂; 48.2% ♀<br>65-74: 6 342<br>75-84: 6 485<br>≥85: 1 308 | 76.5 (6) | Hypertension 68.6%<br>Stroke 40.8%<br>Hyperlipidemia 29.3%<br>Diabetes mellitus 26.9%<br>CKD 7.4% |
| Lai et al. (2010) | China, Taiwan | Population-based case-control study<br>2005-2007 | ≥65 yrs with or without newly diagnosed hip fracture | National health insurance dataset | *n* = 11 640<br>47.8% ♂; 52.2% ♀<br>65-74: 5 772<br>75-84: 4 416<br>≥85: 1 452 | N.R. | N.R. |
| Lakhanpal et al. (2015) | Australia | Observational study (audit)<br>10/2009 to 03/2010 | ≥70 yrs with cancer | Medical oncology clinic | *n* = 304<br>61% ♂; 39% ♀ | 76 [70-95] | *Geriatric syndrome*<br>Files review: 24%<br>Multidisciplinary meeting: 10%<br><br>*Any mention of comorbidity*<br>Files review: 92%<br>Multidisciplinary meeting: 84% |
| Lalic et al. (2016) | Australia | Longitudinal cohort study<br>Follow-up: 1 yr<br>04/2014 to 08/2015 | ≥65 yrs, with or without dementia | Long-term care facilities | *n* = 383<br>22% ♂; 78% ♀ | Median: 88<br>IQR: 84-92 | CCI median (IQR): 2 (1-3) |

| Study | Country | Design & Study period | Subjects | Setting | Sample size | Mean age (SD) [range] | Morbidities or comorbidities[1] |
|---|---|---|---|---|---|---|---|
| M. P. S. Lam, Cheung, and Wong (2015) | China | Cross-sectional study<br>01/2013 to 12/2013 | ≥65 yrs | Acute care tertiary hospital | *n* = 254<br>49.2% ♂; 50.8% ♀ | 81.4 (8.6) | N.R. |
| D. P. Y. Lam et al. (2010) | China | Cross-sectional study (letter to the editor)<br>01/2007 to 12/2007 | ≥65 yrs, outpatient | Medical general or medical geriatrics specialist clinics | *n* = 400<br>43.3% ♂; 56.7% ♀ | 77.2 (7) | Hypertension 63.3%<br>Diabetes mellitus 33%<br>Stroke 30% |
| Lapi et al. (2009) | Italy | Survey<br>2 waves: 1995, 1999 | ≥65 yrs | Community-dwelling | *n* = 568<br>40.1% ♂; 59.9% ♀ | 72.7 (0.2) | CAD or stroke 5.8% to 17%<br>Heart failure 9.3% to 19.7%<br><br>*Nb. of comorbidities, mean (SD)*<br>1995: 1.97 (0.06)<br>1999: 2.59 (0.06) |
| Le Couteur, Hilmer, Glasgow, Naganathan, and Cumming (2004) | Australia | Narrative review | Elderly | N.A. | N.A. | N.A. | N.A. |
| Leavy et al. (2013) | Sweden | Population-based observational study<br>06/2009 to 06/2010 | ≥50 yrs with hip fracture | Community dwelling, residential care facility | *n* = 484<br>30.2% ♂; 69.8% ♀ | 81.7 (9.6) [51-104] | *Pre-fracture comorbidities*<br>CVD 6.9% to 17.2%<br>IHD 16.3% to 23.2%<br>Hypertension 36.4% to 50.4%<br>Dementia 3.5% to 55.6% |

| Study | Country | Design & Study period | Subjects | Setting | Sample size | Mean age (SD) [range] | Morbidities or comorbidities[1] |
|---|---|---|---|---|---|---|---|
| Le Cossec (2015) | France | Methodology study using administrative data (2013) | Individuals aged 75 years and over | Community | *n*=43 619<br>32% ♂; 68% ♀ | *82 (5.28)*<br>*(75-84y:74%; 85-115y:26%)* | *Number of diseases:*<br>*1:35.8%; 2:13.9%; 3:3.9%; >3:0.8%* |
| Leendertse, Egberts, Stoker, and van den Bemt (2008) | Netherland s | Case-control study 09/2005 to 06/2006 | *Study group*<br>Patient with unplanned hospitalization<br><br>*Control group*<br>Patients admitted for elective surgery | Hospital | *Study group*<br>*n* = 332<br>50.6% ♂; 49.4% ♀<br>≥65: 226<br><br>*Control group*<br>*n* = 714<br>50.5% ♂; 49.5% ♀<br>≥65: 221 | *Study group*<br>70 (17)<br><br>*Control group*<br>67 (16) | *Study group*<br>Nb. of diseases<br>1-3: 39.2%<br>≥4: 55.7%<br><br>*Control group*<br>Nb. of diseases<br>1-3: 43.4%<br>≥4: 39.8% |
| Lees (2013) | Australia | Expert opinion | Elderly with cancer | N.A. | N.A. | N.A. | N.A. |
| Lees and Chan (2011) | Australia, Singapore | Narrative review | Elderly with cancer | N.A. | N.A. | N.A. | N.A. |
| Lees (2009) | Australia | Expert opinion (abstract COSA's annual | Elderly with cancer | N.A. | N.A. | N.A. | N.A. |

| Study | Country | Design & Study period | Subjects | Setting | Sample size | Mean age (SD) [range] | Morbidities or comorbidities[1] |
|---|---|---|---|---|---|---|---|
| | | scientific meeting) | | | | | |
| Leiss et al. (2015) | Switzerland | Prospective longitudinal cohort study<br>Follow-up: 2 yrs<br>09/2009 to 09/2012 | ≥65 yrs with Venous Thromboembolism | Hospital, community | *n* = 830<br>53% ♂; 47% ♀ | [65-97]<br>Median: 75 | Hypertension 65.3%<br>Anemia 33.9%<br>Cardiac disease 24%<br>Chronic renal disease 18.4%<br>Diabetes mellitus 15.8% |
| Lichtman and Boparai (2008) | United States | Expert opinion | Elderly with cancer | N.A. | N.A. | N.A. | N.A. |
| Linnebur et al. (2014) | United States | Cross-sectional study<br>2011 to 2012 | 65-89 yrs with depression | Ambulatory clinics | *n* = 200<br>21.5% ♂; 78.5% ♀ | *California*<br>74.3 (7.4)<br><br>*Colorado*<br>*79.7 (6.1)* | Diabetes mellitus 18%<br>Cancer 17%<br>COPD 35%<br>Cancer 30% |
| Liu, Leung, and Chi (2011) | China, United Kingdom, Unites States | Cohort study<br>Follow-up: 1 yr<br>1999 to 2000 | ≥65 yrs | Primary care | *n* = 457<br>48.4% ♂; 51.6% ♀ | 73.6 | N.R. |
| Loffler (2014) | Germany | Protocol of an experimental study<br>Follow-up 1 yr<br>08/2014 | ≥65 yrs with chronic diseases and multi-morbidity, ≥5 drugs | Hospital | *n* = 1 626 | N.R. | N.R. |

| Study | Country | Design & Study period | Subjects | Setting | Sample size | Mean age (SD) [range] | Morbidities or comorbidities[1] |
|---|---|---|---|---|---|---|---|
| López Molina et al. (2012) | Spain | Cross-sectional study<br>01/2006 | ≥65 yrs | Primary Health Care Districts | *n* = 2914<br>39.2% ♂; 60.8% ♀<br>65-69: 792<br>70-74: 804<br>75-79: 580<br>≥80: 738 | 75.2 | Vascular risk 90.2% |
| Lowthian et al. (2016) | Australia | Longitudinal cohort study<br>Follow-up: 6 months<br>07/2012 to 11/2013 | ≥65 yrs triaged as categories 2-5 on ATS | Tertiary hospital: Emergency department | *n* = 959<br>44% ♂; 56% ♀<br>65-74: 396<br>75-84: 350<br>≥85: 213 | Median: 77<br>IQR: 70-84 | Depression 27%<br>Cognitive impairment 26%<br>Falls history 23%<br><br>*Nb. of comorbidities*<br>Median (IQR): 0 (0-1) |
| Lu, Wen, Chen, and Hsiao (2015) | Taiwan | Longitudinal cohort study<br>Follow-up: 10 yrs<br>01/2002 to 12/2011 | ≥65 yrs | Data from National Health insurance research database | *n* = 59 042<br>51.2% ♂; 48.8% ♀<br>65-74: 39 358<br>75-84: 16 903<br>≥85: 2 781 | N.R. | Hypertension 39.4%<br>Arthritis 28.2%<br>CAD 20.9%<br>Diabetes 17.9%<br>CVD 13.4%<br><br>CCI mean (SD): 1.2 (1.3) |
| Luzny, Bellova, and Donek (2010) | Czech Republic | Cross-sectional study<br>02/2009 to 06/2009 | Patients hospitalized in geronto-psychiatric wards | Psychiatric hospital | *n* = 304<br>33.2% ♂; 66.8% ♀ | 71.2 (9.2) | Hypertension 59.9%<br>Visual or hearing disturbances 40.8%<br>Hyperlipidemia 39.8%<br>Cervicalgia, thoracalgia, lumbalgia or sacralgia 32.2%<br>Diabetes mellitus 28.9% |

| Study | Country | Design & Study period | Subjects | Setting | Sample size | Mean age (SD) [range] | Morbidities or comorbidities[1] |
|---|---|---|---|---|---|---|---|
| Maggiore et al. (2014) | United Stated | Prospective longitudinal cohort study | ≥65 yrs with any type of solid-tumor malignancy | Secondary analysis of collected data of adults ≥65 yrs with cancer undergoing chemotherapy | *n* = 500<br>44% ♂; 56% ♀<br>65-69: 175<br>70-74: 127<br>75-79: 105<br>80-84: 73<br>85-91: 20 | 73 (6.2) [65-91] | Hypertension 52%<br>Arthritis 46%<br>Heart disease 20%<br>Stomach disorder 19% |
| Maggiore, Gross, and Hurria (2010) | United States | Literature review | Elderly with cancer | N.A. | N.A. | N.A. | N.A. |
| Maher, Hanlon, and Hajjar (2014) | United States | Literature review, expert opinion 01/1986 to 06/2013 | >65 yrs | Ambulatory care, Hospital, nursing home | *n* = 6 observational studies & 10 RCT | N.A. | N.A. |
| Mamun, Lien, Goh-Tan, and Ang (2004) | Singapore | Cross-sectional study | ≥65 yrs | Nursing homes | *n* = 454<br>33.3% ♂; 66.7% ♀ | 80<br>Median: 80 | N.R. |
| Mannucci and Nobili (2014) | Italy | Review REPOSI registry 2008, 2010, 2012 | Elderly | Medical of geriatric hospital | *n* = 4 035<br>48.2% ♂; 51.8% ♀<br>65-74: 1 242<br>75-84:1 840<br>≥85: 953 | 79 | *CCI mean (SD)*<br>Admission<br>REPOSI 2010: 2.9 (1.7)<br>REPOSI 2012: 3.1 (1.9)<br><br>Discharge<br>REPOSI 2010: 3.0 (1.8)<br>REPOSI 2012: 3.2 (2.0) |

| Study | Country | Design & Study period | Subjects | Setting | Sample size | Mean age (SD) [range] | Morbidities or comorbidities[1] |
|---|---|---|---|---|---|---|---|
| Mansur, Weiss, and Beloosesky (2008) | Israel | Longitudinal cohort study<br>Follow-up: 129 days<br>07/2004 to 06/2005 | ≥65 yrs, received chronic medications | Hospital: Acute geriatric ward | *n* = 212<br>38.2% ♂; 61.8% ♀<br>65-74: 41<br>75-84: 106<br>≥85: 65 | 81.1 (7.3) [66-103] | *Chronic diseases*<br>Mean (SD): 6.3 (2.6)<br>1-4: 23.6%<br>5: 18.9%<br>6: 16.5%<br>≥7: 41.0% |
| Maraldi et al. (2009) | Italy | Narrative review | Elderly | N.A. | N.A. | N.A. | N.A. |
| Marcum et al. (2012) | United States | Retrospective cohort<br>10/2003 to 12/2006 | ≥65 yrs, veterans | Departments of Veterans Affairs Medical Centers: Ambulatory care | *n* = 678<br>98.5% ♂; 1.5% ♀<br>65-74: 273<br>75-84: 334<br>≥85: 71 | 76.4 | Chronic comorbid conditions mean: 4<br><br>≥1 psychiatric condition: 25% |
| Marcum and Gellad (2012) | United States | Systematic review and conceptual model<br>1998 to 07/2011 | Elderly | N.R. | *n* = 9 studies | N.R. | N.A. |
| Marshall (2012) | United States | Narrative review | Elderly with gait disturbance | N.A. | N.A. | N.A. | N.A. |
| Martinez-Selles, Gomez Huelgas, bu-Assi, Calderon, and Vidan (2016) | Spain | Consensus statement<br>6/2015 to 10/2015 | Elderly with chronic IHD | N.A. | N.A. | N.A. | N.A. |

| Study | Country | Design & Study period | Subjects | Setting | Sample size | Mean age (SD) [range] | Morbidities or comorbidities[1] |
|---|---|---|---|---|---|---|---|
| Martens et al (2008) | Canada | Study with administrative data (1996-2004) | ≥65 yrs | Community-dwelling | N.R. | N.R. | N.R. |
| Mastromarino et al. (2014) | Italy | Narrative review | Patient with heart failure | N.A. | N.A. | N.A. | N.A. |
| Mazzone et al. (2016) | Italy | Retrospective cohort study 09/2012 to 01/2014 | ≥65 yrs with atrial fibrillation | Hospital | *n* = 399<br>40.6% ♂; 59.4% ♀ | Median: 85 | CHF 29.3% to 42.4%<br>COPD 12.2% to 33.3%<br>CVD 22.2% to 32.8%<br>Myocardial infarction 17.1 to 30.6%<br>Hemiplegia 2.8% to 26.8%<br><br>CCI median: 2 to 3<br>* According to the medicines consumed |
| McGory, Shekelle, Rubenstein, Fink, and Ko (2005) | United States | Systematic review, expert opinion | Elderly with undergoing abdominal operations | N.R. | N.R. | N.R. | N.A. |
| McMahon, Cahir, Kenny, and Bennett (2014) | Ireland | Before and after design<br>Follow up: 1 yr | ≥70 yrs with fall | Hospital: emergency department | *n* = 1 016<br>30.3% ♂; 69.7% ♀ | 82.7 (6.1)<br>Median: 82.7<br>IQR 77.9-86.8 | *Nb. of comorbidities*<br>Median: 3 to 6 |
| Meloni, Vetrano, Bernabei, | Italy | Narrative review | Elderly with dementia | N.A. | N.A. | N.A. | N.A. |

| Study | Country | Design & Study period | Subjects | Setting | Sample size | Mean age (SD) [range] | Morbidities or comorbidities[1] |
|---|---|---|---|---|---|---|---|
| and Onder (2015) | | | | | | | |
| Min et al. (2013) | United States | Development and validation phase<br>Follow-up: 1 yr<br>2002 | ≥75 yrs with at least one of 3 geriatric conditions (urinary incontinence, impaired memory or falls) | Ambulatory care | *n* = 644<br>33.6% ♂; 66.4% ♀ | 81.2 (4.8) [75-100] | New falls 66.0%<br>Smoking past 44.7%<br>CKD stage 2 43.8%<br>Mild stable anemia 40.8%<br>Chronic pain 40.5%<br><br>CCI mean (SD): 3.7 (2.6) |
| Mira et al. (2013) | Spain | Cross-sectional study<br>11/2011 to 12/2011 | ≥65 yrs with at least one condition (CVD, respiratory, endocrine or digestive disorders), ≥5 drugs | Health centres | *n* = 382<br>57.3% ♂; 42.7% ♀ | 74.6 (6.4) [65-93] | *Nb. of comorbidities*<br>2: 42.1%<br>3: 4.7%<br>4: 4.7% |
| Mirk, Kemp, Echt, and Perkins (2013) | United States | Experimental study (poster)<br>2012 | Elderly veterans taking ≥10 drugs | Geriatrics primary care clinic | *n* = 27<br>11% ♂; 89% ♀ | 89 [85-94] | Dementia 55.6%<br><br>CCI mean: 8.96 |
| Mizokami et al. (2016) | Japan | Cross-sectional study<br>10/2010 to 12/2013 | Elderly with cancer | Hospital | *n* = 3 351<br>35.5% ♂; 64.5% ♀ | 78.9 (6.3) [65-103] | CCI mean (SD): 1.2 (1.5) |

| Study | Country | Design & Study period | Subjects | Setting | Sample size | Mean age (SD) [range] | Morbidities or comorbidities[1] |
|---|---|---|---|---|---|---|---|
| Mizokami, Koide, Noro, and Furuta (2012) | Japan | Cohort study 01/2009 to 12/2009 | >65 yrs with ≥1 prescribed drugs | Hospital: National Center for Geriatrics and Gerontology | *n* = 1 768<br>51.5% ♂; 48.5% ♀ | 78.2 (7.7) [65-100]<br>65-69: 308<br>7074: 346<br>75-79: 425<br>80-84: 330<br>≥85: 359 | Hypertension 41%<br>Hyperlipidemia 28%<br>Gastric ulcer 26%<br>Previous stroke 26%<br>Reflux esophagitis 23%<br><br>CCI mean (SD) [range]: 2.6 (2.6) [0-12] |
| Modig, Kristensson, Ekwall, Hallberg, and Midlov (2009) | Sweden | Questionnaires 09/2006 to 10/2007 | ≥65 yrs, need help for at least 2 ADL | Hospital, primary care, outpatient | *n* = 34<br>41.2% ♂; 58.8% ♀ | ♂: 86 [76-95]<br>♀: 81.5 [72-94] | N.R. |
| Monégat, Sermet, Perronnin & Rococo (2014) | France | Methodology paper (2012-2013) | ≥75 years with at least one medication | Community | *n*=69,324 | N.R. | N.R. |
| Montero-Balosa et al. (2015) | Spain | Experimental study (poster) | ≥65 yrs, ≥5 drugs with potential safety problems[4] | Primary care centres | *n* = 420<br>31% ♂; 69% ♀ | 74 | N.R. |
| Morandi et al. (2013) | Italy | Retrospective longitudinal cohort study Follow-up: 30 days 01/2004 to 06/2011 | ≥65 yrs with an acute hospitalization | Rehabilitation hospital | *n* = 2 735<br>29% ♂; 71% ♀ | Median: 80<br>IQR: 74-85 | CIRS median (IQR): 1.62 (1.46-1.84) |

| Study | Country | Design & Study period | Subjects | Setting | Sample size | Mean age (SD) [range] | Morbidities or comorbidities[1] |
|---|---|---|---|---|---|---|---|
| Moriarty et al (2015) | Ireland | Repeated cross-sectional study using administrative data (1997, 2002, 2007 and 2012) | All population covered by the General Medical Services scheme | Community | *n 338,025 (1997) to 539,752 (2012)*<br>*n 65y+: 78,489 (1997) to 133 884 (2012)* | N.R. | *N.R.* |
| Moulis et al. (2015) | France | Cross-sectional study and methodology study (letter to the editor) 01/2013 to 10/2013 | ≥65 yrs consulting for the first time at the Geriatric frailty clinic | Mostly community dwelling | *n* = 437<br>37.3% ♂; 62.7% ♀ | 83 (6.1) | *CCI score*<br>1-2: 46.2%<br>3-4: 20.1%<br>≥5: 12.6% |
| Munger (2010) | United States | Narrative review | Elderly with hypertension and comorbid diabetes | N.A. | N.A. | N.A. | N.A. |
| Naveiro-Rilo et al. (2014) | Spain | Cross-sectional study | ≥67 yrs on PP | Community | *n* = 369<br>44.6% ♂; 55.6% ♀<br><80: 187<br>≥80: 182 | 79.9 | Hypertension 79.3%<br>Hyper cholesterol 63.2%<br>Diabetes 34.4%<br>Ischemic disease 21.5%<br>CVD 10.8%<br>≥4 comorbidities: 74% |
| Neuner-Jehle (2013) | Switzerland | Literature review | Elderly | N.A. | N.A. | N.A. | N.A. |
| Nguyen, Fouts, Kotabe, and Lo (2006) | United States | Cohort study 10/1998 to 09/1999 | ≥65 yrs | Nursing home | *n* = 335<br>37.6% ♂; 62.4% ♀ | 72 [65-100] | N.R. |

| Study | Country | Design & Study period | Subjects | Setting | Sample size | Mean age (SD) [range] | Morbidities or comorbidities[1] |
|---|---|---|---|---|---|---|---|
| NHS Highland (2015) | United Kingdom | Practice guideline | Frail adults | N.A. | N.A. | N.A. | N.A. |
| Nightingale, Hajjar, Guo, et al. (2015) | United States | Cross-sectional cohort study 01/2011 to 06/2013 | Patients who received an initial comprehensive geriatric Oncology assessment | Data from electronic medical record | *n* = 234<br>36% ♂; 64% ♀ | 79.9 | Oncology patients: 87% with solid tumor<br>CCI, mean: 7.69 |
| Nightingale, Hajjar, Swartz, ndrel-Sendecki, and Chapman (2015) | United States | Retrospective study 01/2011 to 06/2013 | Elderly, outpatient with cancer | Oncology ambulatory center | *n* = 248<br>36% ♂; 64% ♀<br>60-69: 16<br>70-79: 91<br>80-89: 122<br>90-99: 19 | 79.9 (6.8) [61-98] | *Prevalence of comorbidities by disease*<br>Hypertension 75.4%<br>Dyslipidemia 59.3%<br>Osteoarthritis 51.2%<br>Anemia 33.1%<br>Diabetes 31.1% |
| Noale et al. (2016) | Italy | Survey 09/2010 to 10/2011 | ≥65 yrs, type 2 diabetes mellitus, receiving oral antidiabetic treatment | Community | *n* = 1 342<br>52.5% ♂; 47.5% ♀ | 73.3 (5.5) | 1 oral antidiabetic drug: 49.6%<br>≥3 oral antidiabetic drugs: 12.5% |
| Nobili, Franchi, et al. (2011) | Italy | Longitudinal cohort study Follow-up: 1 yr 01/2005 to 12/2005 | ≥65 yrs | Community<br><br>Data from Administrative database of Lombardy Region | *n* = 1 767 239<br>45.8% ♂; 54.2% ♀<br>65-69: 545 595<br>70-74: 540 170<br>75-79: 361 859<br>82-84: 251 832<br>85-89: 99 046<br>90-94: 55 737 | 74.7 (6.9) | N.R. |

| Study | Country | Design & Study period | Subjects | Setting | Sample size | Mean age (SD) [range] | Morbidities or comorbidities[1] |
|---|---|---|---|---|---|---|---|
| Nobili, Licata, et al. (2011) | Italy | Prospective longitudinal cohort study 01/2008 to 12/2008 | ≥65 yrs, participating in the REPOSI 2008 | Hospital | *n* = 1 332<br>41.4% ♂; 58.6% ♀ | 79.4 (7.5) | Hypertension 57.8%<br>Diabetes mellitus 24.0%<br>CHD 23.0%<br>Atrial fibrillation 20.6%<br>COPD 20.0% |
| Nobili, Marengoni, et al. (2011) | Italy | Cross-sectional study 01/2008 to 12/2008 | ≥65 yrs, participating in the REPOSI 2008 | Hospital | *n* = 1 155<br>46.1% ♂; 53.6% ♀ | 79.2 | Hypertension 58.8%<br>Diabetes mellitus 24.3%<br>CHD 22.9%<br>CVD 20.1<br>Atrial fibrillation 20% |
| Nomura and Akazawa (2012) | Japan | Cross-sectional study (abstract) 04/2009 to 03/2010 | ≥60 yrs | Community | *n* = 34 608<br>45% ♂; 55% ♀ | N.R. | N.R. |
| Nomura (2011) | Japan | Cross-sectional study 10/2009 to 12/2009 | ≥65 yrs | Community | *n* = 453<br>34% ♂; 66% ♀ | 76 [65-97] | Hypertension 60.8%<br>Hyperlipidemia 34.7%<br>Gastric abnormal 24.1%<br>Eye disorder 20.3%<br>Heart failure 19.5% |
| Oh et al. (2015) | United States | Systematic review N.R. to 04/2013 | ≥18 yrs with postoperative delirium following hip fracture surgery | Hospital | *n* = 10 studies | Average age ranging from 77 to 86 | N.A. |

| Study | Country | Design & Study period | Subjects | Setting | Sample size | Mean age (SD) [range] | Morbidities or comorbidities[1] |
|---|---|---|---|---|---|---|---|
| Olsson, Runnamo, and Engfeldt (2011) | Sweden | Longitudinal cohort study<br>Follow-up: 1 yr<br>09/2006 to 05/2008 | ≥75 yrs, ≥5 drugs | Community | *n* = 140<br>37.9% ♂; 62.1% ♀ | 83.4 (5) | N.R. |
| Onder et al. (2013) | Czech Republic, England, Finland, France, Germany, Italy, Netherlands | Longitudinal cohort study<br>Follow-up: 1 yr | Residents with advanced cognitive impairment | Nursing home | *n* = 822<br>23.6% ♂; 76.6% ♀ | 84.6 (8] | IHD 19.8%<br>Stroke 16.3%<br>Diabetes 15.7%<br>Heart failure 11.1%<br>Cancer 8.3%<br><br>*Nb. of diseases*<br>Mean (SD): 2.5 to 3.5 (1.3 to 1.9) |
| Onder et al. (2012) | Czech Republic, England, Finland, France, Germany, Italy, Netherlands, Israel | Cohort study<br>01/2009 to 12/2011 | Older residents | Nursing home | *n* = 4 023<br>26.8% ♂; 73.2% ♀ | 83.5 (9.4) | IHD 26.3%<br>Stroke 22.1%<br>Diabetes 21.7%<br>Heart failure 17.7%<br>Cancer 10.7%<br><br>*Nb. of disease*<br>Mean (SD): 2.3 (1.5) |
| Orfila, Garrofe, Tajada, Pavon, and Cegri (2014) | Spain | Cohort study<br>Ongoing follow-up: 7 yrs<br>2005-2012 | ≥70 yrs | Primary health care | *n* = 691<br>42.5% ♂; 57.5% ♀<br>70-74: 281<br>75-79: 229<br>80-84:147<br>≥85: 40 | 76.9 (4.5) [70-91] | Hypertension 65.2%<br>Arthritis 36.1%<br>Diabetes 23.5% |

| Study | Country | Design & Study period | Subjects | Setting | Sample size | Mean age (SD) [range] | Morbidities or comorbidities[1] |
|---|---|---|---|---|---|---|---|
| O'Sullivan et al. (2013) | United Kingdom | Cross-sectional study<br>12/2009 to 09/2010 | ≥65 yrs | Long-term care including nursing homes and long-stay community hospitals | *n* = 732<br>29.8% ♂; 70.2% ♀<br>65-74: 98<br>75-84: 266<br>≥85: 368 | 83.9 (7.7) [65-102]<br><br>Median: 85<br>IQR: 79-89 | Hypertension 46.5%<br>Dementia 43%<br>Incontinence 33.5%<br>CVD 32%<br>Depression 31.6% |
| Pan, Li, Chen, Su, and Wang (2014) | Taiwan | Nested case-control study<br>Follow-up: 1 yr<br>01/2007 to 12/2008 | ≥65 yrs hospitalised, with or without a discharge diagnosis code of fracture along with accidental fall | Data from National Health Insure Research Database | *Fracture & fall*<br>*n* = 5 933<br>30.9% ♂; 69.1% ♀<br>65-74: 1 956<br>75-84: 2 671<br>≥85: 1 306<br><br>*No fracture & fall*<br>*n* = 29 655<br>49.5% ♂; 50.5% ♀<br>65-74: 17 082<br>75-84: 10 270<br>≥85: 2 313 | *Fracture & fall*<br>78.3 (7.5)<br><br>*No fracture & fall*<br>*73.9 (6.7)* | *CCI score*<br>0: 31.5% to 41.8%<br>1: 40.4% to 43.7%<br>2: 12.8% to 17.4%<br>≥3: 5.0% to 7.4% |
| Parian and Ha (2015) | United States | Longitudinal cohort study<br>01/2006-12/2012 | ≥65 yrs with inflammatory bowel disease (Ulcerative colitis or Crohn's disease) | Inflammatory bowel disease referral clinics | *n* = 190<br>49.5% ♂; 50.5% ♀ | *Age at last clinic follow-up*<br>Crohn's disease<br>70.4 (5.4)<br><br>Ulcerative disease<br>70.0 (14.4) | *CCI score*<br>Crohn's disease<br>0-2: 26.3%<br>3-4: 34.7%<br>≥5: 38.9%<br><br>Ulcerative disease<br>0-2: 25.3%<br>3-4: 43.2%<br>≥5: 31.6% |
| Park et al. (2016) | Korea | Longitudinal cohort study<br>01/2008 to 12/2013 | ≥65 yrs with neck and neck squamous cell carcinoma | N.R. | *n* = 229<br>83.8% ♂; 16.2% ♀ | [65-87]<br>Median: 73 | N.R. |

| Study | Country | Design & Study period | Subjects | Setting | Sample size | Mean age (SD) [range] | Morbidities or comorbidities[1] |
|---|---|---|---|---|---|---|---|
| Patterson et al. (2014) | New Zealand, United Kingdom | Systematic review<br>≥ 11/2013 | ≥65 yrs with >1 long-term medical condition and PP | Hospital, nursing homes, home care, community, primary care | *n* = 12 studies<br>22 438 participants<br>34.4% ♂; 65.6% ♀ | 76.4 | N.A. |
| Payne and Avery (2011) | United Kingdom | Expert opinion | Inaccurate | N.A. | N.A. | N.A. | N.A. |
| Pedone et al. (2005) | Italy | Survey<br>1991-1997 | ≥65 yrs | Hospital | *n* = 9 061<br>47.7% ♂; 52.3% ♀ | 77.4 (7) | Cognitive impairment 21% |
| Pervin (2008) | N.R. | Expert opinion | Elderly | N.A. | N.A. | N.A. | N.A. |
| Pimenta and Oparil (2012) | Australia, United States | Narrative review<br>1934 to N.R. | Elderly | N.A. | N.A. | N.A. | N.A. |
| Pire, Fournier, Schoevaerdts, Spinewine, and Swine (2009) | Belgium | Narrative review | Elderly | N.A. | N.A. | N.A. | N.A. |
| Poudel et al. (2015) | Australia | Cohort study<br>01/2013 to 08/2014 | Elderly with frailty | Residential aged care facilities | *n* = 153<br>35.9% ♂; 64.1% ♀ | 83 (8.1)<br><br>Median: 83 | Dementia 67.3%<br>Depression 46.4%<br>Hypertension 35.9%<br>Under nutrition 32%<br>Diabetes 20.9% |

| Study | Country | Design & Study period | Subjects | Setting | Sample size | Mean age (SD) [range] | Morbidities or comorbidities[1] |
|---|---|---|---|---|---|---|---|
| Pozzi et al. (2010) | Italy | Longitudinal cohort study<br>Follow-up: 8 yrs<br>01/1995 to 12/2003 | ≥65 yrs | Community-dwelling | *n* = 1 022<br>42.9% ♂; 57.1% ♀ | 73 (6.8) | *Self-reported disease*<br>Mean (SD): 2.3 (1.6) |
| Prithviraj et al. (2012) | United States | Cross-sectional study nested within a longitudinal study<br>02/2008 to 09/2009 | ≥65 yrs with new cancer diagnosis | Ambulatory oncology clinics | *n* = 117<br>17% ♂; 83% ♀<br>65-74: 65<br>≥ 75: 52 | 74.6 (6.9) | *Nb. of comorbidities*<br>0-4: 55%<br>≥5: 45% |
| Prithviraj, Bagai, Koroukian, Berger, and Owusu (2010) | United States | Cohort study (abstract)<br>04/2008 to 09/2009 | ≥65 yrs with breast, colon and lung cancers | N.R. | *n* = 91 | Median: 74 | N.R. |
| J. A. Pugh et al. (2014) | United States | Longitudinal retrospective cohort study<br>Follow-up: 30 days<br>10/2005 to 09/2006 | ≥65 yrs who received veterans affairs care | Hospital | *n* = 129 400<br>97.9% ♂; 2.1% ♀<br>65-74: 59 145<br>75-84: 57 167<br>≥85: 13 088 | N.R. | *CCI mean (SD)*<br>No readmission: 4.4 (3)<br>Readmission: 5.9 (3.3) |

| Study | Country | Design & Study period | Subjects | Setting | Sample size | Mean age (SD) [range] | Morbidities or comorbidities[1] |
|---|---|---|---|---|---|---|---|
| M. J. V. Pugh et al. (2007) | United States | Longitudinal cohort study 1993 to 1994 with 3 waves every 2 yrs | 65-99 yrs | Community | *n* = 3 050<br>42% ♂; 58% ♀ | 73.1 (6.8) | Hypertension 42%<br>Arthritis 36%<br>Diabetes 24%<br>CVD 9%<br>Stroke 6% |
| Puts et al. (2009) | Canada | Prospective pilot study 03/2007 to 05/2008 | ≥65 yrs with new cancer diagnosis | Hospital | *n* = 112<br>30.4% ♂; 69.6% ♀ | 74.2 (6.0) | Nutrition problems 40.2%<br>Cognitive impairment 25.0%<br>Vision impairment 23.2%<br>Anxiety 21.4%<br>Hearing impairment 13.4%<br><br>*Nb. of comorbidities*<br>1-2: 48.2%<br>≥3. :31.2% |
| Ramage-Morin (2009) | Canada | National Population Health Survey (1998-1999) | ≥65 yrs | All settings | N.R. | N.R. | N.R. |
| Ramirez-Duque et al. (2014) | Spain | Longitudinal cross-sectional study 02/2009 to 19/2010 | ≥18 yrs with non-reversible chronic organ failure | Hospital, home hospitalization or palliative care | *n* = 1 847<br>51% ♂; 49% ♀<br>≥65: 1 709 | 78.7 (10) | ≥2 chronic diseases: 70% |
| Reason, Terner, Moses McKeag, Tipper, and Webster (2012) | Canada | Survey 04/2008 to 06/2008 | ≥65 yrs | Data from Canadian Survey of Experiences, community | *n* = 3 132<br>65-74: 1 750<br>75-84: 1 809<br>≥85: 293 | N.R. | *Nb. of chronic conditions*<br>1: 13%<br>2: 33%<br>≥3: 62% |

| Study | Country | Design & Study period | Subjects | Setting | Sample size | Mean age (SD) [range] | Morbidities or comorbidities[1] |
|---|---|---|---|---|---|---|---|
| Reboredo-Garcia, Mateo, and Casal-Llorente (2014) | Spain | Experimental study | Chronic polymedicated patients | Health centers | *n* = 20 319<br>40% ♂; 60% ♀<br>0-18: 17<br>19-30: 61<br>31-40: 202<br>41-50: 493<br>51-60: 1 429<br>61-70: 3 457<br>71-80: 6 931<br>81-90: 6 734<br>91-103: 995 | N.R. | N.R. |
| Reddy, Gosavi, and Varma (2012) | India | Narrative review | Elderly | N.A. | N.A. | N.A. | N.A. |
| Regroupement des Unités de courte durée régiatriques et des services hospitaliers de gériatrie du Québec (2015) | Canada | Clinical guidelines | Elderly | Nursing homes | N.A. | N.A. | N.A. |
| Riccio, Solinas, Astara, and Mantovani (2007) | Ireland | Cross-sectional cohort study 09/2005 to 11/2009 | ≥65 yrs, women with Alzheimer disease or other types of dementia | Hospital | *n* = 47<br>0% ♂; 100% ♀<br>≥65: 47<br>65-79: 9<br>≥80: 38 | N.R. | N.R. |

| Study | Country | Design & Study period | Subjects | Setting | Sample size | Mean age (SD) [range] | Morbidities or comorbidities[1] |
|---|---|---|---|---|---|---|---|
| Rich (2014) | United States | Clinical recommendations (narrative review) | Elderly with cardiovascular disease | N.A. | N.A. | N.A. | N.A. |
| Richardson, Ananou, Lafortune, Brayne, and Matthews (2011) | United Kingdom | Longitudinal prospective cohort study<br>Follow-up: 18 yrs<br>1991-12/2008 | ≥65 yrs | Community | *n* = 12 423<br>40.3% ♂; 59.7% ♀<br>65-69: 3 132<br>70-74: 3 088<br>75-79: 2 819<br>80-84: 2 097<br>≥85: 1 287 | N.R. | Heart attack 34%<br>Angina pectoris 34%<br>Parkinson's disease 33%<br>Asthma 31%<br>Epilepsy 29% |
| Riker and Setter (2012) | United States | Continuing Edudcation | Elderly | Community | N.A. | N.A. | N.A. |
| Rohrer, Garrison, Oberhelman, and Meunier (2013) | United states | Cross-sectional study | ≥65 yrs, rehospitalized or not within 30 days | Hospital | *n* = 142 | N.R. | N.R. |
| Romana, Kamath, Sarda, Muraraiah, and Jayanthi (2012) | India | Prospective cross-sectional study<br>06/2010 to 07/2010 | ≥60 yrs on PP | Tertiary care hospital | *n* = 100<br>62% ♂; 38% ♀<br>60-69: 66<br>70-79: 24<br>80-89: 10 | N.R. | N.R. |

| Study | Country | Design & Study period | Subjects | Setting | Sample size | Mean age (SD) [range] | Morbidities or comorbidities[1] |
|---|---|---|---|---|---|---|---|
| Rozenfeld, Fonseca, and Acurcio (2008) | Brazil | Cross-sectional study<br>03/2003 to 08/2003 | ≥60 yrs | Institute of social security | *n* = 577<br>39% ♂; 61% ♀<br>60-69: 237<br>70-79: 231<br>≥80: 109 | N.R. | Hypertension 59.9%<br>Bone/articular disease 42.6%<br>Diabetes 17.0%<br><br>*Nb. of diseases*<br>1-2: 29.7%<br>3-4: 42.2%<br>≥5: 22.9% |
| Ruberu and Fitzgerald (2012) | United stated | Expert opinion | N.R. | N.A. | N.A. | N.A. | N.A. |
| Ruggiero et al. (2010) | Italy | Longitudinal cohort study<br>Follow-up: 1yr<br>02/2004 to 12/2005 | ≥65 yrs | Nursing home | *n* = 1 716<br>28.3% ♂; 71.7% ♀ | 83.6 (8.1) | Dementia 46.2%<br>Hypertension 44.3%<br>Chronic constipation 37.9%<br>Insomnia 36.8%<br>COPD 23.0%<br><br>*Nb of diseases*<br>Mean (SD): 4.0 (2.3)<br><br>CIRS mean (SD): 10.1 (6.2) |
| Runganga, Peel, and Hubbard (2014) | Australia | Prospective cohort study<br>11/2009 to 09/2010 | ≥70 yrs or ≥50 yrs, indigenous population entering the *Transition Care Program* | Transition from hospital to home | *n* = 347<br>34.3% ♂; 65.7% ♀ | 78.9 (8.8) | CHD 34.1%<br>Diabetes 26.3%<br>Depression 21.4%<br>COPD 13.1% |

| Study | Country | Design & Study period | Subjects | Setting | Sample size | Mean age (SD) [range] | Morbidities or comorbidities[1] |
|---|---|---|---|---|---|---|---|
| Salahudeen (2015) | New Zealand | Cohort study 01/2011 to 12/2011 | ≥65 yrs | Community | *n* = 537 387<br>45.1% ♂; 54.9% ♀<br>65-74: 296 100<br>75-84: 172 501<br>≥85: 68 786 | 74.7 (7.6) | N.R. |
| Saarelainen et al. (2014) | United Kingdom | Cohort study 01-2009 to 07/2010 | ≥70 yrs | Hospital | *n* = 385<br>59% ♂; 41% ♀<br>≥70: 385<br>70-74: 147<br>75-79: 132<br>≥80: 106 | 76.7 | Hypertension 58.4%<br>Hypercholesterolemia 32.5%<br>Arthritis 29.4%<br>Diabetes 25.5%<br>Airways disease 24.6%<br><br>CCI 0-2: 66.5%<br>CCI ≥3: 33.5% |
| Salazar, Poon, and Nair (2007) | United States | Expert opinion | Elderly | N.A. | N.A. | N.A. | N.A. |
| Salwe, Kalyansundaram, and Bahurupi (2016) | India | Cross-sectional study (prescription audit) 04/2013 to 06/2013 | ≥70 yrs | Tertiary care hospital | *n* = 100<br>62% ♂; 38% ♀<br>65-74: 70<br>75-84: 21<br>≥85: 9 | 71.6 (6.5) | N.R. |

| Study | Country | Design & Study period | Subjects | Setting | Sample size | Mean age (SD) [range] | Morbidities or comorbidities[1] |
|---|---|---|---|---|---|---|---|
| Sanglier, Saragoussi, Milea, and Tournier (2015) | United States | Cohort study 01/2003 to 12/2006 | *Elderly:* ≥65 yrs with a new episode of depression and without antidepressant claims during the previous 3 months<br><br>*Non-elderly:* 25-64 yrs with the same characteristic as the elders | Community | *n* = 6 316<br>29.7% ♂; 70.3% ♀<br>≥65: 4 440<br>25-64: 25 264 | 77 | *Elderly*<br>Treated depression 16.7%<br>Untreated depression 12.7%<br>Anxiety 12.0% |
| Santibáñez Beltrán et al. (2013) | Mexico | Study evaluating costs | ≥70 yrs with polypharmacy, chronic degenerative disease and ≥1 medical visit per year | Primary health care | *n* = 131<br>50.4% ♂; 49.6% ♀ | 69.21 | Hypertension 89.3%<br>Diabetes 59.5%<br>Degenerative joint disease 23.7%<br>Chronic obstructive pulmonary disease 3.8%<br>Hypothyroidism 3.1% |
| Sato and Akazawa (2013) | Japan | Retrospective cohort study 1981-1999 | ≥65 yrs with hypertension | Community | *n* = 61 661<br>41.8% ♂; 48.2% ♀<br>65-74: 21 643<br>≥75: 40 018 | N.R. | N.R. |
| Scheidt-Nave et al. (2013) | Germany | Speaker presentation, (Survey results 1998 to 11/2008) | 65-79 yrs | Community Data from national health interview surveys | N.R. | N.R. | N.R. |
| Schonenberger et al. (2011) | Switzerland | Longitudinal cohort | Elderly in nursing homes | Nursing home | *n* = 1249 | N.R. | N.R. |

| Study | Country | Design & Study period | Subjects | Setting | Sample size | Mean age (SD) [range] | Morbidities or comorbidities[1] |
|---|---|---|---|---|---|---|---|
| Schrijver, Toppinga, de Vries, Kramer, and Nanayakkara (2013) | Netherlands | Cohort study 11/2011 to 01/2012 | ≥70 yrs | Hospital: emergency department | *n* = 100<br>35% ♂; 65% ♀ | [70-97]<br>Median: 81 | *Nb. of comorbidities,* mean (SD): 3.04 (1.9)<br>≥3 comorbidities: 47%<br>Cardiovascular 85<br>Malignancy 30<br>CVD 29<br>Endocrine disorder 20<br>Neuropsychiatric 16 |
| Schuler et al. (2008) | Austria | Prospective cohort study 02/2007 to 05/2007 | ≥75 yrs | Hospital: departments of gastroenterology and cardiology | *n* = 543<br>39.8% ♂; 60.2% ♀ | 82.6 (5) | CCI mean (SD): 3.2 (2)<br><br>*Nb. of diagnoses*<br>1: 61.5%<br>2: 33%<br>≥3: 5.5% |
| Scott et al. (2012) | Australia | Conceptual framework for minimising inappropriate medications | Elderly | N.A. | N.A. | N.A. | N.A. |
| Scott et al. (2015) | Australia | Literature review | Elderly | N.A. | N.A. | N.A. | N.A. |
| Scottish Govern. Polypharmacy Guidance (2015) | Scotland | Guidance paper | | N.A. | N.A. | N.A. | N.A. |
| Segal, Staum, and Munshi (2009) | United States | Cohort study (abstract) | Elderly with diabetes | Geriatric diabetes clinic | *n* = 139 | 78.8 (6.5) | Hypoglycemia 44% to 69%<br>Under nutrition 7% to 63%<br>Depression 31% to 53%<br>Cognitive dysfunction 25% to 53% |

| Study | Country | Design & Study period | Subjects | Setting | Sample size | Mean age (SD) [range] | Morbidities or comorbidities[1] |
|---|---|---|---|---|---|---|---|
| Sehgal et al. (2013) | United States | Retrospective nested case-control study 01/2008 to 12/2009 | Patients readmitted to the hospital within 30 days of discharge | Hospital | *n* = 414<br>41.1% ♂; 58.9% ♀ | 79.25 | N.R. |
| Sengstock and Zimmerman (2014) | United States | Book chapter | Elderly | N.A. | N.A. | N.A. | N.A. |
| Sergi, De Rui, Sarti, and Manzato (2011) | Italy | Expert opinion, narrative review | Elderly | N.A. | N.A. | N.A. | N.A. |
| Sganga et al. (2015) | Italy | Longitudinal cohort study Follow-up: 1 yr 06/2010 to 05/2011 | ≥65 yrs | Hospital: geriatric and internal medicine acute care | *n* = 480<br>47.3% ♂; 52.7% ♀ | 78.6 (6.8) | Diabetes 32.5%<br>IHD 31.5%<br>Heart failure 25.6%<br>Stroke 7.3%<br>Dementia 7.1%<br><br>CCI mean (SD): 2.1 (1.7) |

| Study | Country | Design & Study period | Subjects | Setting | Sample size | Mean age (SD) [range] | Morbidities or comorbidities[1] |
|---|---|---|---|---|---|---|---|
| Sganga et al. (2014) | Italy | Prospective cohort study 06/2010 to 05/2011 | ≥65 yrs | Geriatric and internal medicine acute care wards Date from CRIME project | *n* = 1 123<br>44% ♂; 56% ♀ | 81.5 (7.4) | Ischemic heart failure 31.7%<br>Diabetes 29.7%<br>Heart failure 27.3%<br>Dementia 20.5%<br>Cancer 13.8%<br><br>*Nb of diseases*<br>Mean (SD): 5.7 (3.1) |
| Shah and Hajjar (2012) | United States | Narrative review | Elderly | N.A. | N.A. | N.A. | N.A. |
| Sharifi, Hasanloei, and Mahmoudi (2014) | Germany | Review 01/2001 to 12/2011 | N.A. | N.A. | N.A. | N.A. | N.A. |
| Shastry, Ullal Sheetal, Sowjanya, Bethi, and Kumar (2015) | India | Retrospective cross-sectional study | ≥60 yrs with cardiovascular disease, outpatient | Outpatient department of a tertiary care hospital | *n* = 204<br>50% ♂; 50% ♀<br>60-65: 63<br>66-70: 57<br>71-75: 44<br>76-80: 29<br>≥80: 11 | 69.7 (6.7) | Diabetes mellitus 53.4% |
| Shimizu et al. (2014) | Japan | Cross-sectional study (abstract) | ≥65 yrs, functionally independent | Community-dwelling | *n* = 2 044<br>49.6% ♂; 50.4% ♀ | N.R. | N.R. |
| Silvestre (2015) | Portugal | Multicentered randomized controlled trial (abstract) | ≥65 yrs on PP | Nursing home | *n* = 126 | N.R. | N.R. |

| Study | Country | Design & Study period | Subjects | Setting | Sample size | Mean age (SD) [range] | Morbidities or comorbidities[1] |
|---|---|---|---|---|---|---|---|
| Singler et al. (2014) | Germany | Prospective cohort study 03/2010 to 04/2010 | ≥75 yrs | Hospital: emergency department | *n* = 133<br>39.8% ♂; 60.2% ♀ | Median: 83.2 | CCI score ≥3: 49.6%<br>Delirium 14.3% |
| Skoldunger, Fastbom, Wimo, Fratiglioni, and Johnell (2015) | Switzerland | Cohort study 01/2001 to 12/2004 | ≥60 yrs | Data from Swedish National study on aging and care | *n* = 4 108<br>37.2% ♂; 62.8% ♀<br>60-69: 1 559<br>70-79: 1 127<br>80-89: 872<br>≥90: 550 | 74.8 (11.1) [60-105] | CCI score 0: 58.8%<br>CCI score 1: 18.2%<br>CCI score 2: 15.6%<br>CCI score ≥3 : 7.5%<br><br>Dementia 7.8% |
| Slabaugh, Maio, Templin, and Abouzaid (2010) | Italy | Retrospective cohort study Follow-up: 1 yr 01/2007 to 12/2007 | ≥65 yrs with at least one prescription filled during the study year | Community<br><br>Data from Emilia-Romagna outpatient prescriptions database | *n* = 887 165<br>41.7% ♂; 58.3% ♀<br>65-74: 437 275<br>75-84: 334 327<br>≥85: 115 563 | 75.5 (7.5) | N.R. |
| Sokol, Knudsen, and Li (2007) | United States | Retrospective cohort study | ≥70 yrs, outpatient undergoing chemotherapy | Community-dwelling, primary care facility | *n* = 100<br>55% ♂; 45% ♀ | 78<br>IR: 70-90 | *Nb. of comorbidities*<br>Mean: 3 |
| Soleymani (2010) | Iran | Cross-sectional household survey (abstract) 01/2007 to 12/2007 | ≥60 yrs | Community | *n* = 1 054<br>49.4% ♂; 50.6% ♀ | N.R. | N.R. |

| Study | Country | Design & Study period | Subjects | Setting | Sample size | Mean age (SD) [range] | Morbidities or comorbidities[1] |
|---|---|---|---|---|---|---|---|
| Sonnichsen et al. (2016) | United Kingdom | Study protocol for RCT<br>Follow-up: 2yrs<br>Recruitment: 01/2015 to 09/2015 | ≥75 yrs on PP | Community | N.A. | N.A. | N.A. |
| Srinivasan et al. (2016) | United States | Cohort study<br>01/2006 to 04/2014 | Patients with ruptured abdominal aortic aneurysm | Vascular surgical center | *n* = 184<br>85% ♂; 15% ♀ | Median: 77 | N.R. |
| Stauder, Spross, and Augscholl (2015) | Australia | Cohort study (pilot) (abstract) | Elderly with myelodysplastic syndromes, acute myeloid leukemia or chronic myelomonocytic leukaemia | N.R. | *n* = 64<br>56.3% ♂; 43.7% ♀ | Median: 78.5 | CCI score >2: 40.6% |
| Stawicki and Gerlach (2009) | United States | Narrative review | Elderly | N.A. | N.A. | N.A. | N.A. |
| Steinman (2007) | United States | Editorial | Elderly | N.A. | N.A. | N.A. | N.A. |
| Steinman et al. (2006) | United States | Cross-sectional study<br>01/2001 to 12/2003 | ≥65 yrs on PP | Outpatient in Veterans Affairs Medical Center | *n* = 196<br>99% ♂; 1% ♀ | 74.6 (5.4) | Hypertension 89%<br>IHD 57%<br>Diabetes mellitus 45%<br>COPD 26%<br>Heart failure 12% |

| Study | Country | Design & Study period | Subjects | Setting | Sample size | Mean age (SD) [range] | Morbidities or comorbidities[1] |
|---|---|---|---|---|---|---|---|
| Strehblow, Smeikal, and Fasching (2014) | Austria | Cohort study 01/2010 to 12/2012 | ≥80 yrs | Hospital | *n* = 948<br>33% ♂; 67% ♀ | 87.2 (4.5) | *CCI mean (SD)*<br>80-84 yrs: 2.2 (1.7)<br>85-89 yrs: 2.2 (1.7)<br>90-94 yrs: 2.0 (1.6)<br>≥95 yrs: 1.7 (1.1) |
| Sumukadas, McMurdo, Mangoni, and Guthrie (2014) | Scotland | Cohort study 03/1995 to 03/2010 | ≥65 yrs registered with a Tayside General Practice | Entire population | *1995*<br>*n* = 67 608<br>38.9% ♂; 60.1% ♀<br><br>*2010*<br>*n* = 73 465<br>43.2% ♂; 56.8% ♀ | *1995*<br>74.6 (7.2)<br><br>*2010*<br>75.1 (7.4) | N.R. |
| Tam-McDevitt (2008) | N.R. | Narrative review | Elderly with cancer | N.A. | N.A. | N.A. | N.A. |
| Tan et al. (2015) | USA | Prospective cohort study | ≥65 yrs with no dementia and candidates for elective colon surgery | Hospital | *n* = 44<br>66% ♂; 34% ♀ | 75.5 | Hypertension 45.5%<br>Dyslipidemia 27.3%<br>Colon/rectal cancer 18.2%<br>Gastroesophageal reflux 13.6% |

| Study | Country | Design & Study period | Subjects | Setting | Sample size | Mean age (SD) [range] | Morbidities or comorbidities[1] |
|---|---|---|---|---|---|---|---|
| Thai, Hilmer, Pearson, Reeve, and Gnjidic (2015) | Australia | Cross-sectional study 07/2014 to 10/2014 | ≥65 yrs, prescribed a statin | Hospital: cardiovascular, geriatric, orthopaedic or general medicine | *n* = 180<br>52.8% ♂; 47.2% ♀ | Median :78<br>IQR: 14 | CCI, median (IQR): 2 (3) |
| Thurmann (2013) | Germany | Narrative review | Elderly | N.A. | N.A. | N.A. | N.A. |
| Torrance, Powell, and Griffiths (2015) | United Kingdom | Narrative review | Elderly | N.A. | N.A. | N.A. | N.A. |
| Tulner et al. (2010) | Netherland s | Cross-sectional analysis of cohort study | ≥1 following conditions: hypertension, angina pectoris, CVD, ischemic attack, atrial disease or fibrillation, myocardial infraction, heart failure, diabetes, osteoporosis | Community | *n* = 516<br>47% ♂; 53% ♀ | 81.5 | CCI, mean (SD): 2.3 (1.4) |

| Study | Country | Design & Study period | Subjects | Setting | Sample size | Mean age (SD) [range] | Morbidities or comorbidities[1] |
|---|---|---|---|---|---|---|---|
| Tulner et al. (2009) | Netherlands | Survey<br>05/2004 to 08/2004 | ≥60 yrs with ≥1 prescription after hospital discharge | Community | *n* = 40<br>65% ♂; 35% ♀ | 82.1 | N.R. |
| Turner et al. (2016) | Australia | Methodology study<br>01/2009 to 07/2010 | ≥70 yrs, attending the geriatric oncology outpatient multi-disciplinary clinic | Hospital: geriatric oncology multi-disciplinary clinic | *n* = 385<br>59% ♂; 41% ♀ | 76.7 (4.8) [70-92] | Hypertension 58.4%<br>Hypercholesterolemia 32.5%<br>Arthritis 29.4%<br>Diabetes 25.5%<br>Airways disease 24.6% |
| Turner et al. (2014) | Australia | Cohort study<br>01/2009 to 07/2010 | ≥70 yrs, outpatient with cancer | Medical oncology clinic | *n* = 385<br>59% ♂; 41% ♀ | 76.7 (4.8) [70-92] | CCI, mean (SD): 1.2 (1.4)<br><br>Hypertension 62.0%<br>Hypercholesterolemia 34.4%<br>Arthritis 31.1%<br>Diabetes 27.0%<br>Airways disease 26.2% |
| Ussai, Casetta, Bresigar, Barbone, and Pisa (2013) | Italy | Longitudinal cohort study (abstract)<br>11/2012 to 04/2013 | ≥65 outpatient | External care | *n* = 180<br>42.7% ♂; 57.3% ♀ | 76.6 (9.4) | CVD 33.9%<br>Diabetes 14.4%<br>Cancer 4.4%<br>Depression 3.9% |
| van der Marck et al. (2014) | United Kingdom | Expert opinion | Patient with Parkinson | N.A. | N.A. | N.A. | N.A. |

| Study | Country | Design & Study period | Subjects | Setting | Sample size | Mean age (SD) [range] | Morbidities or comorbidities[1] |
|---|---|---|---|---|---|---|---|
| Vande Walle et al. (2014) | England | Prospective cohort study 10/2009 to 07/2011 | ≥70 yrs with cancer | Hospital | *n* = 937<br>36.5% ♂; 63.5% ♀ | [70-95]<br>Median: 76 | New cancer 61%<br>Congestive heart failure 18.4%<br>Diabetes without complication 13.4%<br>CCI 1: 24.8%<br>CCI ≥2: 29.1% |
| Vass and Hendriksen (2005) | Denmark | Expert opinion | GP of older people | N.A. | N.A. | N.A. | N.A. |
| Venturini et al. (2011) | Brazil | Retrospective cohort study 01/2006 to 05/2007 | ≥60, able-bodied | Community | *n* = 438 | N.R. | N.R. |
| Vetrano et al. (2013) | Czech Republic, England, Finland, France, Germany, Italy, Netherlands | Cross-sectional study 2009 to 2010 | Elderly with severe cognitive impairment | Nursing homes | *n* = 1 449<br>25% ♂; 75% ♀ | 84.2 (9) | IHD 24.6%<br>Stroke 24.2%<br>Diabetes mellitus 19.6%<br>Heart failure 14.6%<br>Cancer 9.2%<br><br>*Nb. of diagnoses*<br>Mean (SD): 2.6 (1.5) |
| Vigouroux, Bastard, and Capeau (2014) | France | Expert opinion | HIV-infected individual | N.A. | N.A. | N.A. | N.A. |

| Study | Country | Design & Study period | Subjects | Setting | Sample size | Mean age (SD) [range] | Morbidities or comorbidities[1] |
|---|---|---|---|---|---|---|---|
| Viktil, Blix, Moger, and Reikvam (2007) | Norway | Prospective cohort study 05/2002 to 12/2002 | Patients | Hospital | *n* = 827<br>41.4% ♂; 58.6% ♀ | <5 drugs<br>75.4 (12.8) [21-98]<br><br>≥5 drugs<br>66.7 (19.4) [15-98] | Cardiac failure 18.0%<br>Diabetes mellitus 11.2%<br>Reduced renal function 18.9%<br>Reduced liver function 1.9% |
| Viktil, Blix and Reikvam (2008) | Norway | Expert review | Individuals with polypharmacy, often elderly | N.A. | N.A. | N.A. | N.A. |
| Vivas-Consuelo, Uso-Talamantes, Trillo-Mata, and Mendez-Valera (2015) | Spain | Expert review | Elderly | N.A. | N.A. | N.A. | N.A. |
| Walsh and Cussen (2010) | Ireland | Before-after study without control group Follow-up: 4 weeks | ≥65 yrs receiving repeat prescriptions for ≥2 drugs, living at home | Community | *n* = 50<br>44% ♂; 56% ♀ | 73 [65-86] | N.R. |

| Study | Country | Design & Study period | Subjects | Setting | Sample size | Mean age (SD) [range] | Morbidities or comorbidities[1] |
|---|---|---|---|---|---|---|---|
| Wang et al. (2015) | China | Longitudinal cohort study<br>Follow-up: 4yrs<br>2009-2014 | ≥80 yrs, men only | Hospital | *n* = 1 562<br>100% ♂; 0% ♀ | 85.2 [80-104] | Hypertension 52.1%<br>IHD 33.5%<br>Pain 28.7%<br>Diabetes 24.5%<br>Frail 18.9% |
| Wauters, Elseviers, Vaes, Degryse, Dalleur, Vander Stichele, Van Bortel, et al. (2016) | Belgium | Prospective cohort study<br>11/2008 to 09/2009 | ≥80 yrs, non-institutionalised with drugs records, able to visit their GP | Population-based | *n* = 503<br>38.8% ♂; 61.2% ♀ | 83.9 (3.6) [80-102] | Hypertension 70.4%<br>Osteoarthritis 57.1%<br>Hyperlipidaemia 44.1%<br>Heart failure 38.4%<br>Diabetes 18.9%<br><br>*Multimorbidity*<br>Median (SD) [range]: 3.9 (1.6) [1-9] |
| Wauters, Elseviers, Vaes, Degryse, Vander Stichele, et al. (2016) | Belgium | Prospective longitudinal cohort study<br>Follow-up: 1.5 yrs<br>10/2008 | ≥80 yrs without known dementia and not in acute or palliative care | Community | *n* = 503<br>38.8% ♂; 61.2% ♀ | 84.4 [80-102] | Hypertension 70.4%<br>Osteoarthritis 57.1%<br>Hyperlipidaemia 44.1%<br>Heart failure 38.4%<br>Diabetes 18.9%<br><br>*Multimorbidity*<br>Median [range]: 4 [1-9] |

| Study | Country | Design & Study period | Subjects | Setting | Sample size | Mean age (SD) [range] | Morbidities or comorbidities[1] |
|---|---|---|---|---|---|---|---|
| Wauters, Elseviers, Vaes, Degryse, Dalleur, Vander Stichele, Christiaens, et al. (2016) | Belgium | Longitudinal cohort study<br>Follow-up: 1.5 yrs<br>11/2008 to 09/2009 | ≥80 yrs, primary care patients | Community-dwelling | *n* = 503<br>38.8% ♂; 61.2% ♀ | 84.4 [80-102] | Hypertension 70.4%<br>Osteoarthritis 57.1%<br>Hyperlipidaemia 44.1%<br>Heart failure 38.4%<br>Obesity 27.9% |
| Wawruch, Fialova, et al. (2008) | Slovakia | Retrospective cohort study<br>12/2003 to 03/2005 | ≥65 yrs | Hospital | *n* = 600<br>41.5% ♂; 58.5% ♀:<br>65-74: 245<br>≥75: 355 | 76.6 | N.R. |
| Wawruch, Zikavska, et al. (2008) | Slovakia | Retrospective cohort study<br>12/2003 to 03/2005 | ≥65 yrs | Hospital | *n* = 600<br>41.5% ♂; 58.5% ♀ | 76.6 (6.5) | *Without PP*<br>≥4 diseases: 35.3%<br>IHD 76.1%<br>Hypertension 69.3%<br>Osteoporosis and arthritis 42.0%<br>Heart failure 24.4%<br>Diabetes mellitus 25.1<br><br>*With PP*<br>≥4 diseases: 53.6%<br>IHD 82.3%<br>Hypertension 81.8%<br>Diabetes mellitus 44.8%<br>Osteoporosis and arthritis 42.3%<br>Heart failure 40.6% |

| Study | Country | Design & Study period | Subjects | Setting | Sample size | Mean age (SD) [range] | Morbidities or comorbidities[1] |
|---|---|---|---|---|---|---|---|
| Wehling and Throm (2015) | Germany | Narrative review /expert opinion | Elderly | N.R. | N.R. | N.R. | N.R. |
| Wehling (2009) | Germany | Letter to the editor | Elderly | N.R. | N.R. | N.R. | N.R. |
| Wehrberger, Madersbacher, Jungwirth, Fischer, and Tragl (2012) | Austria | Population-based cohort study<br>Follow-up: 10 yrs<br>01/2010 to 12/2010 | ≥75 yrs, likely to survive for 10 yrs | Community | *n* = 262<br>63.4% ♂; 36.6% ♀<br>≥75: 262 | 85 | Urinary incontinence 32.4%<br>Neurovascular disease 16.8%<br>Coronary heart disease 13.7%<br>Alzheimer' dementia 10.3%<br>Diabetes 11.1% |
| Westerholm (2013) | Sweden | Narrative review | Elderly | N.A. | N.A. | N.A. | N.A. |
| Williams et al. (2004) | United States | Experimental study - RCT<br>06/1993 to 11/1995 | ≥65 yrs, cognitively intact, ≥5 drugs | Health center ambulatory clinic | *n* = 140<br>42.9% ♂; 57.1% ♀ | 74 (5.6 to 5.9) | N.R. |
| Wilson et al. (2011) | Australia | Cohort study<br>Follow-up: 1 yr | ≥70 yrs, likely to survive for 12 months | Community | *n* = 602<br>29.1% ♂; 70.9% ♀ | 85.7 | Nb. od comorbidities, mean (SD): 0.95 (0.91)<br>Arthritis 39.0%<br>Stroke 21.3%<br>Osteoporosis 16.6%<br>Diabetes 14.3%<br>Parkinson 3.5% |

| Study | Country | Design & Study period | Subjects | Setting | Sample size | Mean age (SD) [range] | Morbidities or comorbidities[1] |
|---|---|---|---|---|---|---|---|
| Wimmer, Dent, Bell, et al. (2014) | Australia | Cohort study<br>Follow-up: 1yr<br>10/2010 to 12/2011 | ≥70 yrs | Hospital: Geriatrics evaluation and Management unit | *n* = 163<br>27.6% ♂; 72.4% ♀ | 84.9 (6.2) | CCI score range 0-10 |
| Wimmer, Dent, Visvanathan, et al. (2014) | Australia | Prospective cohort study<br>10/2010 to 12/2011 | ≥70 yrs | Hospital: Geriatric evaluation and Management unit | *n* = 163<br>27.6% ♂; 72.4% ♀ | 85.2 (6.4) | Cardiovascular disease 82.2%<br>Gastrointestinal 47.2%<br>Arthritis 36.8%<br>Diabetes 33.1%<br>Urinary tract Infection/urological disorders 28.8% |
| Wise (2013) | England | Expert opinion | All | N.A. | N.A. | N.A. | N.A. |
| Womack, Brandt, and Justice (2015) | United States | Review | Women aging with HIV | N.A. | N.A. | N.A. | N.A. |
| Wong, Marr, Kwan, Meiyappan, and Adcock (2014) | Canada | Cross-sectional retrospective chart review<br>04/2010 to 03/2011 | ≥65 yrs, >4 visits to an emergency department associated with the hospital network | Primary care clinic | *n* = 46<br>50% ♂; 50% ♀<br>65-74: 18<br>75-84: 24<br>≥85: 4 | 76.3 (6.6) | CCI, mean (SD): 3.7 (2.6) |

| Study | Country | Design & Study period | Subjects | Setting | Sample size | Mean age (SD) [range] | Morbidities or comorbidities[1] |
|---|---|---|---|---|---|---|---|
| Wright et al. (2009) | United States | Cross-sectional analysis of cohort study 08/1995 to 01/1999 | ≥65 yrs, hospitalized in a medical or surgical ward for >48 hours, and meet criteria for frailty | Community | *n* = 384<br>97.4% ♂; 2.6% ♀<br>65-74: 206<br>75-85: 165<br>>85: 13 | N.R. | CCI mean (SD): 2.44 (1.93)<br>Depression 9.6% |
| Wu et al. (2006) | Hong Kong | Experimental study – RCT 10/1998 to 06/2001 | Patients on ≥5 drugs on at least 2 consecutives visits to the clinic | Hospital medical clinic | *n* = 442 | N.R. | N.R. |
| Wyles and Rehman (2005) | United Kingdom | Expert opinion | Elderly | N.A. | N.A. | N.A. | N.A. |
| Yang, Lee, Huang, Shih, and Chang (2015) | Taiwan | Longitudinal cohort study | ≥65 yrs | Community | *n* = 2 415<br>52.9% ♂; 47.1% ♀ | N.R. | Hypertension 41.74%<br>Cataract 38.22%<br>Impaired cognition 26.69%<br>Depression 24.76%<br>Heart disease 23.60% |
| Yong, Lau, Li, Hakendorf, and Thompson (2012) | Australia | Cohort study 03/2009 | Elderly | Medical centre | *n* = 200<br>39.5% ♂; 60.5% ♀<br>≤64: 42<br>65-84: 74<br>≥85: 84 | 74.9 (17.2) | Hypertension 41%<br>Hypercholesterolemia 25%<br>Type 2 diabetes 20%<br>COPD 19%<br>Gastroesophageal reflux disease 11% |

| Study | Country | Design & Study period | Subjects | Setting | Sample size | Mean age (SD) [range] | Morbidities or comorbidities[1] |
|---|---|---|---|---|---|---|---|

**Notes:** [1] Morbidities or comorbidities reported by the autrhors; [2] The comorbidity index was calculated based on the presence of the following conditions: atrial fibrillation, cancer, chronic obstructive pulmonary disease, depression, dementia, diabetes, epilepsy (treated), heart failure, myocardial infarction, other psychiatric disorders (psychosis, schizophrenia, or bipolar affective disorder), renal disease, and stroke;[3] *mild comorbidity* when there was no comorbidity higher than grade 2 and less than three grade 2 comorbidities, *moderate comorbidity* when there were a maximum of two grade 3 comorbidities and no grade 4 comorbidities, or *severe comorbidity* when there were three grade 3 comorbidities or any grade 4 comorbidity; [4] Patients should at least have one of the following potential safety problems: a) concomitant use of an antihypertensive drug with a non-steroidal anti-inflammatory drug, anticoagulant or antitrombotic drug; b) use of two or more benzodiazepines;
(BZD).

**Abbreviations:** ADL: activities of daily living; ATS: Australasian Triage System; BZD: benzodiazepine; CAD: coronary artery disease; CCI: charlson comorbidity index; CHD: coronary heart disease; CHF: congestive heart failure; CIRS: cumulative Illness rating scale; CKD: chronic kidney disease; COPD: chronic obstructive pulmonary disease; CRIME: CRiteria to Assess Appropriate Medication Use among Elderly Complex Patients; CVD: cerebrovascular disease; DSD: digestive system disease; FCI: functional comorbidity index; GP: general practitioners; GPA: geriatric-palliative approach; IHD: Ischemic heart disease; IQR: interquartile range; MCI: mild cognitive impairment; *n* : number of participants; nb: number; N.R.: not reported; PP: polypharmacy; PSM: potentially suboptimal medicines; PVD: peripheral vascular disease; RCT: randomized controlled trial; RSD: respiratory system disease; REPOSI: Registro Politherapie SIMI; SD: standard deviation; SWEOLD: Swedish Panel Study of Living Condition of the Oldest; tx, treatment; yr : year

Abraham, F., Varughese, G., Mathew, J. C., John, P. M., & Sam, G. K. (2015). Drug utilization pattern among geriatric patients in a tertiary care teaching Hospital. *Asian Journal of Pharmaceutical and Clinical Research, 8*(6), 191-194.

Advinha, A. M., de Oliveira-Martins, S., Mateus, V., Pajote, S. G., & Lopes, M. J. (2014). Medication regimen complexity in institutionalized elderly people in an aging society. *Int J Clin Pharm, 36*(4), 750-756.

Age Care Branch of the Department of Human Services (Victoria). Public sector residential age care quality of care performance indicator project report. ISBN 07311 6198 X. (2004)

Ahmed, B., Nanji, K., Mujeeb, R., & Patel, M. J. (2014). Effects of Polypharmacy on Adverse Drug Reactions among Geriatric Outpatients at a Tertiary Care Hospital in Karachi: A Prospective Cohort Study. *PLoS ONE, 9*(11), e112133. doi:10.1371/journal.pone.0112133

Akazawa, M., Imai, H., Igarashi, A., & Tsutani, K. (2010). Potentially inappropriate medication use in elderly japanese patients. *American Journal Geriatric Pharmacotherapy, 8*, 146-160.

All Wales Medicines Strategy Group. Polypharmacy: Guidance for Prescribing. July 2014.

Anpalahan, M., & Gibson, S. (2008). Geriatric syndromes as predictors of adverse outcomes of hospitalization. *Internal medicine journal, 38*(1), 16-23.

Anthierens, S., Tansens, A., Petrovic, M., & Christiaens, T. (2010). Qualitative insights into general practitioners views on polypharmacy. *BMC Fam Pract, 11*, 65. doi:10.1186/1471-2296-11-65

Antonio, M., Saldana, J., Fernandez, P., & Navarro, V. (2013). The impact of geriatric assessment on the treatment decision of elderly cancer patients. *European Journal of Cancer, 49*, S347.

Azana Fernandez, E. H., & Davila Barboza, Y. R. (2013). Inappropriate prescribing in hospitalised Spanish elderly according to 2012 updated beers' criteria and STOPP criteria. *4*(European Geriatric Medicine), S179.

Azana Fernandez, E. H., & Davila Barboza, Y. R. (2015). STOPP/START criteria version 2 and 2012 Beers' criteria: Are they complementary? *European Geriatric Medicine, 6*, S137.

Badgwell, B., Stanley, J., Chang, G. J., Katz, M. H. G., Lin, H. Y., Ning, J., . . . Cormier, J. N. (2013). Comprehensive geriatric assessment of risk factors associated with adverse outcomes and resource utilization in cancer patients undergoing abdominal surgery. *Journal of Surgical Oncology, 108*, 182-186.

Bahat, G., Tufan, F., Bahat, Z., Aydin, Y., Tufan, A., Akpinar, T. S., . . . Karan, M. A. (2013). Assessments of functional status, comorbidities, polypharmacy, nutritional status and sarcopenia in Turkish community-dwelling male elderly. *Aging Male, 16*(2), 67-72. doi:10.3109/13685538.2013.771329

Bahat, G., Tufan, F., Bahat, Z., Tufan, A., Aydin, Y., Akpinar, T. S., . . . Karan, M. A. (2014). Comorbidities, polypharmacy, functionality and nutritional status in Turkish community-dwelling female elderly. *Aging Clin Exp Res, 26*(3), 255-259. doi:10.1007/s40520-014-0229-8

Bain, K. T., Holmes, H. M., Beers, M. H., Maio, V., Handler, S. M., & Pauker, S. G. (2008). Discontinuing Medications: A Novel Approach for Revising the Prescribing Stage of the Medication-Use Process. *Journal of the American Geriatrics Society, 56*(10), 1946-1952.

Baker, P. N., Salar, O., Ollivere, B. J., Forward, D. P., Weerasuriya, N., Moppett, I. K., & Moran, C. G. (2014). Evolution of the hip fracture population: Time to consider the future? A retrospective observational analysis. *BMJ Open, 4*(4), e004405. doi:doi: 10.1136/bmjopen-2013-004405.

Balducci, L., Goetz-Parten, D., & Steinman, M. A. (2013). Polypharmacy and the management of the older cancer patient. *Annals of Oncology, 24 Suppl 7*, vii36-40. doi:10.1093/annonc/mdt266

Ballentine, N. H. (2008). Polypharmacy in the elderly: maximizing benefit, minimizing harm. *Critical care nursing quarterly, 31*(1), 40-45.

Banerjee, A., Mbamalu, D., Ebrahimi, S., Khan, A. A., & Chan, T. F. (2011). The prevalence of polypharmacy in elderly attenders to an emergency department - a problem with a need for an effective solution. *International Journal of Emergency Medicine, 4*(1), 22. doi:10.1186/1865-1380-4-22

Baranzini, F., Diurni, M., Ceccon, F., Poloni, N., Cazzamalli, S., Costantini, C., . . . Callegari, C. (2009). Fall-related injuries in a nursing home setting: is polypharmacy a risk factor? *BMC health services research, 9*(1), 228.

Barnett, N., & Oboh, L. (2008). Target old people with medication risks. *Pharmaceutical Journal, 280*, 276.

Beauplet, B., & Martin, A. (2015). Polypharmacy and potentially inappropriate medication in elderly patients treated for multiple myeloma. *Fundamental and Clinical Pharmacology, 29*, 60.

Beer, C., Hyde, Z., Almeida, O. P., Norman, P., Hankey, G. J., Yeap, B. B., & Flicker, L. (2011). Quality use of medicines and health outcomes among a cohort of community dwelling older men: An observational study. *British Journal of Clinical Pharmacology, 71*, 592-599.

Belfrage, B., Koldestam, A., Sjoberg, C., & Wallerstedt, S. M. (2015). Number of drugs in the medication list as an indicator of prescribing quality: a validation study of polypharmacy indicators in older hip fracture patients. *Eur J Clin Pharmacol, 71*(3), 363-368. doi:10.1007/s00228-014-1792-9

Bell, S. P., Orr, N. M., Dodson, J. A., Rich, M. W., Wenger, N. K., Blum, K., . . . Forman, D. E. (2015). What to expect from the evolving field of geriatric cardiology. *Journal of the American College of Cardiology, 66*(11), 1286-1299.

Bell, S. P., & Saraf, A. (2014). Risk stratification in very old adults: How to best gauge risk as the basis of management choices for patients aged over 80. *Progress in Cardiovascular Diseases, 57*(2), 197-203.

Beloosesky, Y., Nenaydenko, O., Gross Nevo, R. F., Adunsky, A., & Weiss, A. (2013). Rates, variability, and associated factors of polypharmacy in nursing home patients. *Clin Interv Aging, 8*, 1585-1590. doi:10.2147/CIA.S52698

Bennett, A., Gnjidic, D., Gillett, M., Carroll, P., Matthews, S., Johnell, K., . . . Hilmer, S. (2014). Prevalence and impact of fall-risk-increasing drugs, polypharmacy, and drug-drug interactions in robust versus frail hospitalised falls patients: a prospective cohort study. *Drugs Aging, 31*(3), 225-232. doi:10.1007/s40266-013-0151-3

Best, O., Gnjidic, D., Hilmer, S. N., Naganathan, V., & McLachlan, A. J. (2013). Investigating polypharmacy and drug burden index in hospitalised older people. *Internal medicine journal, 43*(8), 912-918. doi:10.1111/imj.12203

Best Practice Advocacy Centre New Zealand. Polypharmacy in primary care: Managing a clinical conumdrum. Best Practice Journal 2014; 64; 5-14.

Blanco-Reina, E., Ariza-Zafra, G., Gonzalez-Correa, J. A., & Leon-Ortiz, M. (2011). Polypharmacy and pattern of medication use among the elderly. *Basic and Clinical Pharmacology and Toxicology, 109*, 67.

Blanco-Reina, E., riza-Zafra, G., Ocana-Riola, R., Leon-Ortiz, M., & Bellido-Estevez, I. (2015). Optimizing elderly pharmacotherapy: Polypharmacy vs. undertreatment. Are these two concepts related? *European Journal of Clinical Pharmacology, 71*(2), 199-207.

Boffin, N., Moreels, S., Vanthomme, K., & Van Casteren, V. (2014). Falls among older general practice patients: a 2-year nationwide surveillance study. *Family Practice, 31*(3), 281-289.

Bokhof, B., & Junius-Walker, U. (2016). Reducing Polypharmacy from the Perspectives of General Practitioners and Older Patients: A Synthesis of Qualitative Studies. *Drugs Aging, 33*(4), 249-266. doi:10.1007/s40266-016-0354-5

Boparai, M. K., & Korc-Grodzicki, B. (2011). Prescribing for older adults. *The Mount Sinai journal of medicine, 78*(4), 613-626. doi:10.1002/msj.20278

Boparai, M. K., Korc-Grodzicki, B., Mahmoudzadeh Pournaki, S., & Shahrokni, A. (2015). Prevalence of polypharmacy in older adults with cancer who underwent intra-abdominal surgeries and its relationship to geriatric and clinical outcomes. *Journal of the American Geriatrics Society, 63*, S90.

Bowling, C. B., Booth, J. N., Gutierrez, O. M., Tamura, M. K., Huang, L., Kilgore, M., . . . Muntner, P. (2014). Nondisease-specific problems and all-cause mortality among older adults with CKD: The REGARDS study. *Clinical Journal of the American Society of Nephrology, 9*(10), 1737-1745.

Bradley, M. C., Fahey, T., Cahir, C., Bennett, K., O'Reilly, D., Parsons, C., & Hughes, C. M. (2012). Potentially inappropriate prescribing and cost outcomes for older people: a cross-sectional study using the Northern Ireland Enhanced Prescribing Database. *Eur J Clin Pharmacol, 68*(10), 1425-1433. doi:10.1007/s00228-012-1249-y

Brandt, N. (2006). Geriatric polypharmacy: Unraveling the mystery. *Advanced Studies in Medicine, 6*, 182-188.

Bregnhøj, L., Thirstrup, S., Kristensen, M. B., Bjerrum, L., & Sonne, J. (2009). Combined intervention programme reduces inappropriate prescribing in elderly patients exposed to polypharmacy in primary care. *Eur J Clin Pharmacol, 65*(2), 199-207.

Brito, G. D. C., Menezes, M. S., Mesquita, A. R., & Lyra Jr, D. P. (2009). Effect of a drug therapy management program in a group of elderly patients with hypertension in Aracaju (Sergipe, Brazil). *Revista de Ciencias Farmaceuticas Basica e Aplicada, 30*, 83-89.

Bronskill, S. E., Gill, S. S., Paterson, J. M., Bell, C. M., Anderson, G. M., & Rochon, P. A. (2012). Exploring Variation in Rates of Polypharmacy Across Long Term Care Homes. *Journal of the American Medical Directors Association, 13*, 309.

Burkhardt, H., & Wehling, M. (2010). Difficulties in pharmacotherapy of the elderly. *Internist, 51*, 737-748.

Bushardt, R. L., Massey, E. B., Simpson, T. W., Ariail, J. C., & Simpson, K. N. (2008). Polypharmacy: misleading, but manageable. *Clin Interv Aging, 3*(2), 383-389.

Cadogan, C. A., Ryan, C., Francis, J. J., Gormley, G. J., Passmore, P., Kerse, N., & Hughes, C. M. (2015). Improving appropriate polypharmacy for older people in primary care: selecting components of an evidence-based intervention to target prescribing and dispensing. *Implement Sci, 10*, 161. doi:10.1186/s13012-015-0349-3

Cahir, C., Fahey, T., Teeling, M., Teljeur, C., Feely, J., & Bennett, K. (2010). Potentially inappropriate prescribing and cost outcomes for older people: a national population study. *British Journal of Clinical Pharmacology, 69*(5), 543-552.

Cakmur, H. (2015). Frailty among elderly adults in a rural area of Turkey. *Medical Science Monitor, 21*, 1232-1242. doi:10.12659/MSM.893400

Canadian Institute for Health Information. Health Care in Canada, 2011: A focus on seniors and aging. Ottawa, ON. CIHI; 2011

Carneiro, S. C. D. S., Azevedo-e-Silva, M. C., & Ramos-e-Silva, M. (2011). Drug eruptions in the elderly. *Clinics in Dermatology, 29*, 43-48.

Carrasco-Garrido, P., Lopez De Andres, A., Barrera, V. H., Jimenez-Trujillo, I., & Jimenez-Garcia, R. (2013). National trends (2003-2009) and factors related to psychotropic medication use in community-dwelling elderly population. *International Psychogeriatrics, 25*, 328-338.

Castro-Alvirena, J. D., Verdejo-Bravo, C., Gil Gregorio, P., & Lebreault Polanco, A. M. (2014). Efficacy of geriatric intervention on polypharmacy consumption. Experience in an outpatient geriatric service. *European Geriatric Medicine, 5*, S222.

Caughey, G., Vitry, A., Gilbert, A., & Roughead, E. (2009). Polypharmacy in chronic diseases in the elderly: Evidence-based or potentially inappropriate prescribing? *Pharmacoepidemiology and Drug Safety (PDS), 18*, S197-S198.

Chan, D.-C., Hao, Y.-T., & Wu, S.-C. (2009). Polypharmacy among disabled Taiwanese elderly: a longitudinal observational study. *Drugs & Aging, 26*(4), 345-354.

Chang, C. B., Chen, J. H., Wen, C. J., Kuo, H. K., Lu, I. S., Chiu, L. S., . . . Chan, D. C. (2011). Potentially inappropriate medications in geriatric outpatients with polypharmacy: application of six sets of published explicit criteria. *British journal of clinical pharmacology, 72*(3), 482-489. doi:10.1111/j.1365-2125.2011.04010.x

Chen, C. C. H., Kenefick, A. L., Tang, S. T., & McCorkle, R. (2004). Utilization of comprehensive geriatric assessment in cancer patients. *Critical Reviews in Oncology/Hematology, 49*, 53-67.

Cherubini, A., Eusebi, P., Dell'Aquila, G., Landi, F., Gasperini, B., Bacuccoli, R., . . . Ruggiero, C. (2012). Predictors of hospitalization in Italian nursing home residents: the U.L.I.S.S.E. project. *Journal of the American Medical Directors Association, 13*(1), 84 e85-10. doi:10.1016/j.jamda.2011.04.001

Chung, J.-Y. (2014). Geriatric clinical pharmacology and clinical trials in the elderly. *Translational and Clinical Pharmacology, 22*(2), 64-69.

Classen, S., Meuleman, J., Garvan, C., Ried, L. D., Mann, W., & Asal, N. (2007). Review of prescription medications in home-based older adults with stroke: a pilot study. *Research in social & administrative pharmacy, 3*(1), 104-122. doi:10.1016/j.sapharm.2006.06.002

Clyne, B., Bradley, M. C., Hughes, C., Fahey, T., & Lapane, K. L. (2012). Electronic prescribing and other forms of technology to reduce inappropriate medication use and polypharmacy in older people: a review of current evidence. *Clinics in geriatric medicine, 28*(2), 301-322. doi:10.1016/j.cger.2012.01.009

Collerton, J., Jagger, C., Yadegarfar, M. E., Davies, K., Parker, S. G., Robinson, L., & Kirkwood, T. B. L. (2016). Deconstructing Complex Multimorbidity in the Very Old: Findings from the Newcastle 85+ Study. *BioMed Research International, 2016*, no.

Crentsil, V., Ricks, M. O., Xue, Q.-L., & Fried, L. P. (2010). A pharmacoepidemiologic study of community-dwelling, disabled older women: factors associated with medication use. *Am J Geriatr Pharmacother, 8*(3), 215-224.

Cronin, J., Livhits, M., Mercado, C., Chen, F., Foster, N., Chandler, C., . . . Chen, D. C. (2011). Quality improvement pilot program for vulnerable elderly surgical patients. *American Surgeon, 77*, 1305-1308.

Dalleur, O., Spinewine, A., Henrard, S., Losseau, C., Speybroeck, N., & Boland, B. (2012). Inappropriate prescribing and related hospital admissions in frail older persons according to the STOPP and START criteria. *Drugs Aging, 29*(10), 829-837. doi:10.1007/s40266-012-0016-1

Davila Barboza, Y. R., & Azana Fernandez, E. H. (2013a). Outcome of elderly hip fracture patients admitted to an acute orthogeriatric unit. *European Geriatric Medicine, 4*, S68.

Davila Barboza, Y. R., & Azana Fernandez, E. H. (2013b). Polypharmacy in elderly patients: Influence of hospitalization. *European Geriatric Medicine, 4*, S189-S190.

De Groot, M. H., Van Campen, J. P. C. M., Kosse, N. M., De Vries, O. J., Beijnen, J. H., & Lamoth, C. J. C. (2016). The association of medication-use and frailty-related factors with gait performance in older patients. *PLoS ONE, 11*(2), e0149888.

De Saint-Hubert, M., Schoevaerdts, D., Poulain, G., Cornette, P., & Swine, C. (2009). Risk factors predicting later functional decline in older hospitalized patients. *Acta Clinica Belgica, 64*, 187-194.

Denneboom, W., Dautzenberg, M. G. H., Grol, R., & De Smet, P. A. G. M. (2006). Analysis of polypharmacy in older patients in primary care using a multidisciplinary expert panel. *British journal of general practice, 56*(528), 504-510.

Denneboom, W., Dautzenberg, M. G. H., Grol, R., & De Smet, P. A. G. M. (2007). Treatment reviews of older people on polypharmacy in primary care: cluster controlled trial comparing two approaches. *British journal of general practice, 57*(542), 723-731.

Diez-Manglano, J., Barquero-Romero, J., Mena, P. A., Recio-Iglesias, J., Cabrera-Aguilar, J., Lopez-Garcia, F., . . . Soriano, J. B. (2014). Polypharmacy in patients hospitalised for acute exacerbation of COPD. *European Respiratory Journal, 44*, 791-794.

Diez-Manglano, J., Gimenez-Lopez, M., Garces-Horna, V., Sevil-Puras, M., Castellar-Otin, E., Gonzalez-Garcia, P., . . . Morlanes-Navarro, T. (2015). Excessive polypharmacy and survival in polypathological patients. *European Journal of Clinical Pharmacology, 71*(6), 733-739.

Divakaran, V. G., & Murugan, A. T. (2008). Polypharmacy: An undervalued component of complexity in the care of elderly patients. *European Journal of Internal Medicine, 19*, 225-226.

Doan, J., Zakrzewski Jakubiak, H., Roy, J., Turgeon, J., & Tannenbaum, C. (2013). Prevalence and risk of potential cytochrome P450-mediated drug-drug interactions in older hospitalized patients with polypharmacy. *The Annals of pharmacotherapy, 47*(3), 324-332.

Doupe M, Brownell M, Kozyrskyj A, Dik N, Burchill C, Dahl M, Chateau D, De Coster C, Hinds A, Bodnarchuck J. Using Data to develop indicators of quality care in personal care homes. Winnipeg, Manitoba Centre for Health Policy, October 2006.

Doupe M, Finlayson G, Han S, Yogendran M, Shcultz J, McDougall C, Kulbalba C. Supportive housing for seniors: Reform implications for Manitoba's older adult continuum of care. Winnipeg, MB. Manitoba Centre for health Policy, Spring 2016.

Doran DM, Hirdes J, White N, Baker GR, Blais R, Pickard J, Jantzi M. The nature and burden of safety problems among Canadian home care clients. Evidence from the RAI-HC reporting system for three provinces and one territory. May 2009. 37 pages

Drenth-van Maanen, A. C., van Marum, R. J., Knol, W., van der Linden, C. M., & Jansen, P. A. (2009). Prescribing optimization method for improving prescribing in elderly patients receiving polypharmacy: results of application to case histories by general practitioners. *Drugs Aging, 26*(8), 687-701. doi:10.2165/11316400-000000000-00000

Duerden M, Avery T, Payne R. Polypharmacy and medicines optimisation. Making it safe and sound. The King's Fund 2013. Available at: http://www.kingsfund.org.uk/sites/files/kf/field/field_publication_file/

Dwyer, L. L., Han, B., Woodwell, D. A., & Rechtsteiner, E. A. (2010). Polypharmacy in nursing home residents in the United States: Results of the 2004 National Nursing Home Survey. *American Journal Geriatric Pharmacotherapy, 8*, 63-72.

Eckardt, R., Steinhagen-Thiessen, E., Kampfe, S., & Buchmann, N. (2014). Polypharmacy and drug prescription in the elderly. Strategies for optimization. *Z Gerontol Geriatr, 47*(4), 293-301.

Egan, P. E., O'Connor, E. M., Conde, S., Fitzgerald, A. P., & O'Toole, P. W. (2012). Associations between polypharmacy, functional ability and nutritional status in elderly Irish subjects in long term care (the ELDERMET project). *European Geriatric Medicine, 3*, S23.

Eijsink, M., Zeeman, M., Van Wijngaarden, J., Badings, E., & Van'T Riet, E. (2015). Does polypharmacy in elderly patients with heart failure influence mortality and hospitalisation? *Clin Ther, 37*(8 SUPPL. 1), e81-e82.

Elliot, K., Tooze, J. A., Geller, R., Powell, B. L., Pardee, T. S., Ritchie, E., . . . Klepin, H. D. (2014). The prognostic importance of polypharmacy in older adults treated for acute myelogenous leukemia (AML). *Leukemia Research, 38*, 1184-1190.

Ervin, K. (2014). Utilising the screening tool to alert doctors to the right treatment with the screening tool of older people's potentially inappropriate prescriptions. *Australasian Journal on Ageing, 33*(1), 63. doi:10.1111/ajag.12112

Espino, D., Bazaldua, O., Palmer, R., Mouton, C., Parchman, M., Miles, T., & Markides, K. (2006). Suboptimal medication use and mortality in an older adult community-based cohort: results from the Hispanic EPESE Study. *J Gerontol A Biol Sci Med Sci, 61*(2), 170-175.

Farrell, B., Shamji, S., Monahan, A., & French Merkley, V. (2013). Reducing polypharmacy in the elderly: Cases to help you "rock the boat". *Canadian Pharmacists Journal, 146*(5), 243-244.

Fastbom J, Johnell K. National indicators for quality of drug therapy in older persons: the Swedish experience from the first 10 years. Drugs Aging (2015);32:189-199.

Fialova, D., & Onder, G. (2009). Medication errors in elderly people: contributing factors and future perspectives. *British Journal of Clinical Pharmacology, 67*(6), 641-645. doi:10.1111/j.1365-2125.2009.03419.x

Finkelstein, J., Friedman, C., Hripcsak, G., & Cabrera, M. (2016). Pharmacogenetic polymorphism as an independent risk factor for frequent hospitalizations in older adults with polypharmacy: a pilot study. *Pharmacogenomics and Personalized Medicine 9*, 107-116. doi:10.2147/PGPM.S117014

Flood, K. L., Carroll, M. B., Le, C. V., & Brown, C. J. (2009). Polypharmacy in hospitalized older adult cancer patients: experience from a prospective, observational study of an oncology-acute care for elders unit. *Am J Geriatr Pharmacother, 7*(3), 151-158. doi:10.1016/j.amjopharm.2009.05.002

Flood, K. L., Rohlfing, A., Le, C. V., Carr, D. B., & Rich, M. W. (2007). Geriatric syndromes in elderly patients admitted to an inpatient cardiology ward. *Journal of Hospital Medicine, 2*, 394-400.

Franchi, C., Cartabia, M., Risso, P., Mari, D., Tettamanti, M., Parabiaghi, A., . . . Nobili, A. (2013). Geographical differences in the prevalence of chronic polypharmacy in older people: eleven years of the EPIFARM-Elderly Project. *Eur J Clin Pharmacol, 69*(7), 1477-1483. doi:10.1007/s00228-013-1495-7

Franchi, C., Marcucci, M., Mannucci, P. M., Tettamanti, M., Pasina, L., Fortino, I., . . . Nobili, A. (2016). Changes in clinical outcomes for community-dwelling older people exposed to incident chronic polypharmacy: a comparison between 2001 and 2009. *Pharmacoepidemiology and drug safety, 25*(2), 204-211. doi:10.1002/pds.3938

Franchini, M., Pieroni, S., Fortunato, L., Molinaro, S., & Liebman, M. (2014). Poly-pharmacy among the Elderly: Analyzing the Co-morbidity of Hypertension and Diabetes. *Curr Pharm Des*.

Frank, C. (2014). Deprescribing: a new word to guide medication review. *Canadian Medical Association Journal, 186*(6), 407-408. doi:10.1503/cmaj.131568

Frazier, S. (2005). Health outcomes and polypharmacy in elderly individuals: an integrated literature review. *Journal of Gerontological Nursing, 31*(9), 4-11.

Fried, T. R., O'Leary, J., Towle, V., Goldstein, M. K., Trentalange, M., & Martin, D. K. (2014). Health outcomes associated with polypharmacy in community-dwelling older adults: a systematic review. *Journal of the American Geriatrics Society, 62*(12), 2261-2272.

Frohnhofen, H., Michalek, C., & Wehling, M. (2011). [Assessment of drug treatment in geriatrics with the new FORTA criteria]. *Deutsche medizinische Wochenschrift 136*(27), 1417-1421. doi:10.1055/s-0031-1281530

Frutos Bernal, E., Martín Corral, J., & Galindo Villardón, P. (2011). Factors related to polypharmacy in the non-institutionalised elderly. Analysis of the subsample of the national survey of health 2006 for the elderly in Castile-Leon. *Revista española de geriatría y gerontología, 46*(6), 303-306.

Fulton, M., & Allen, E. (2005). Polypharmacy in the elderly: a literature review. *Journal of the American Academy of Nurse Practitioners, 17*(4), 123-132.

Fustinoni, S., Renard, D., Santos-Eggimann, B., & Seematter-Bagnoud, L. (2015). Polypharmacy and associated factors among community-dwelling older persons in a Swiss canton. *Clin Ther, 37*(8 SUPPL. 1), e39-e40.

Galazzi, A., Lusignani, M., Chiarelli, M. T., Mannucci, P. M., Franchi, C., Tettamanti, M., . . . Nobili, A. (2016). Attitudes towards polypharmacy and medication withdrawal among older inpatients in Italy. *Int J Clin Pharm, 38*(2), 454-461. doi:10.1007/s11096-016-0279-4

Gallagher, P., Lang, P. O., Cherubini, A., Topinkova, E., Cruz-Jentoft, A., Montero Errasquin, B., . . . O'Mahony, D. (2011). Prevalence of potentially inappropriate prescribing in an acutely ill population of older patients admitted to six European hospitals. *European Journal of Clinical Pharmacology, 67*, 1175-1188.

Gamble, J. M., Hall, J. J., Marrie, T. J., Sadowski, C. A., Majumdar, S. R., & Eurich, D. T. (2014). Medication transitions and polypharmacy in older adults following acute care. *Therapeutics and Clinical Risk Management, 10*, 189-196.

Garcia-Baztan, A., Roqueta, C., Martinez-Fernandez, M. I., Colprim, D., Puertas, P., & Miralles, R. (2014). Benzodiazepine prescription in the elderly in different health care levels: Characteristics and related factors. *Revista Espanola de Geriatria y Gerontologia, 49*, 24-28.

Garfinkel, D., Ilhan, B., & Bahat, G. (2015). Routine deprescribing of chronic medications to combat polypharmacy. *Therapeutic Advances in Drug Safety, 6*(6), 212-233.

Garfinkel, D., Zur-Gil, S., & Ben-Israel, J. (2007). The war against polypharmacy: a new cost-effective geriatric-palliative approach for improving drug therapy in disabled elderly people. *The Israel Medical Association journal, 9*(6), 430-434.

Garrido-Garrido, E. M., Garcia-Garrido, I., Garcia-Lopez-Duran, J. C., Garcia-Jimenez, F., Ortega-Lopez, I., & Bueno-Cavanillas, A. (2011). [Study of polymedicated patients over 65 years-old in an urban primary care centre]. *Revista de calidad asistencial, 26*(2), 90-96. doi:10.1016/j.cali.2010.10.002

Gentes, T., Hertzog, M., Vogel, T., & Lang, P. O. (2015). Potentially inappropriate prescribing cardiovascular medications in the aged population: Prospective study in a district hospital centre (France). *Presse Medicale, 44*(2), e41-e50.

Girones, R., Torregrosa, D., & Diaz-Beveridge, R. (2010). Comorbidity, disability and geriatric syndromes in elderly breast cancer survivors. Results of a single-center experience. *Critical Reviews in Oncology/Hematology, 73*, 236-245.

Gnjidic, D., Hilmer, S. N., Blyth, F. M., Naganathan, V., Cumming, R. G., Handelsman, D. J., . . . Le Couteur, D. G. (2012). High-risk prescribing and incidence of frailty among older community-dwelling men. *Clinical Pharmacology & Therapeutics 91*(3), 521-528. doi:10.1038/clpt.2011.258

Gnjidic, D., Hilmer, S. N., Blyth, F. M., Naganathan, V., Waite, L., Seibel, M., . . . Le Couteur, D. G. (2012). Polypharmacy cutoff and outcomes: five or more medicines were used to identify community-dwelling older men at risk of different adverse outcomes. *Journal of Clinical Epidemiology, 65*(9), 989-995.

Gomez, C., Vega-Quiroga, S., Bermejo-Pareja, F., Medrano, M. J., Louis, E. D., & ito-Leon, J. (2015). Polypharmacy in the Elderly: A Marker of Increased Risk of Mortality in a Population-Based Prospective Study (NEDICES). *Gerontology, 61*(4), 301-309.

Gomm, W., Von Holt, K., Thome, F., Broich, K., Maier, W., Fink, A., . . . Haenisch, B. (2016). Association of proton pump inhibitors with risk of dementia: A pharmacoepidemiological claims data analysis. *JAMA Neurology, 73*(4), 410-416.

Greene, M., Steinman, M. A., McNicholl, I. R., & Valcour, V. (2014). Polypharmacy, drug-drug interactions, and potentially inappropriate medications in older adults with human immunodeficiency virus infection. *Journal of the American Geriatrics Society, 62*, 447-453.

Haefeli, W. E. (2014). Polypharmacy and drug therapy in elderly patients–A balance between application, adaptation and discontinuing medication. *Der Klinikarzt, 43*(09), 406-411.

Haider, S. I., Johnell, K., Thorslund, M., & Fastbom, J. (2008). Analysis of the association between polypharmacy and socioeconomic position among elderly aged > or =77 years in Sweden. *Clin Ther, 30*(2), 419-427. doi:10.1016/j.clinthera.2008.02.010

Haider, S. I., Johnell, K., Weitoft, G. R., Thorslund, M., & Fastbom, J. (2009). The influence of educational level on polypharmacy and inappropriate drug use: A register-based study of more than 600,000 older people. *Journal of the American Geriatrics Society, 57*, 62-69.

Hajjar, E., Cafiero, A., & Hanlon, J. (2007). Polypharmacy in elderly patients. *Am J Geriatr Pharmacother, 5*(4), 345-351.

Halling, A., Fridh, G., & Ovhed, I. (2006). Validating the Johns Hopkins ACG Case-Mix System of the elderly in Swedish primary health care. *BMC Public Health, 6*, 171. doi:10.1186/1471-2458-6-171

Hamaker, M. E., Seynaeve, C., Wymenga, A. N. M., van Tinteren, H., Nortier, J. W. R., Maartense, E., . . . Smorenburg, C. H. (2014). Baseline comprehensive geriatric assessment is associated with toxicity and survival in elderly metastatic breast cancer patients receiving single-agent chemotherapy: results from the OMEGA study of the Dutch breast cancer trialists' group. *Breast (Edinburgh, Scotland), 23*(1), 81-87.

Haque, R. (2009). ARMOR: a tool to evaluate polypharmacy in elderly persons. *Annals of Long-Term Care, 17*(6), 26-30.

Harugeri, A., Joseph, J., Parthasarathi, G., Ramesh, M., & Guido, S. (2010a). Potentially inappropriate medication use in elderly patients: a study of prevalence and predictors in two teaching hospitals. *Journal of Postgraduate Medicine, 56*(3), 186-191. doi:10.4103/0022-3859.68642

Harugeri, A., Joseph, J., Parthasarathi, G., Ramesh, M., & Guido, S. (2010b). Prescribing patterns and predictors of high-level polypharmacy in the elderly population: A prospective surveillance study from two teaching hospitals in India. *Am J Geriatr Pharmacother, 8*(3), 271-280. doi:10.1016/j.amjopharm.2010.06.004

Health Quality & Safety Commission New Zealand. Methodology / Polypharmacy Atlas 2017. https://www.hqsc.govt.nz/assets/Health-Quality-Evaluation/Atlas/PolypharmacySFOct17/Methodology_polypharmacy.pdf (Accessed April 8, 2019)

Heppner, H. J., Christ, M., Gosch, M., Muhlberg, W., Bahrmann, P., Bertsch, T., . . . Singler, K. (2012). Polypharmacy in the elderly from the clinical toxicologist perspective. *Z Gerontol Geriatr, 45*(6), 473-478. doi:10.1007/s00391-012-0383-6

Hernandez, M., Ceballos, N., Martin, G., & Salazar, R. (2011). Causes of hospitalizations of nursing home residents. *European Geriatric Medicine, 2*, S46-S47.

Hernandez Perella, J. A., Mas Garriga, X., Riera Cervera, D., Quintanilla Castillo, R., Gardini Campomanes, K., & Torrabadella Fabregas, J. (2013). Inappropriate prescribing of drugs in older people attending primary care health centres: Detection using STOPP-START criteria. *Revista Espanola de Geriatria y Gerontologia, 48*, 265-268.

Hilmer, S. N., & Gnjidic, D. (2009). The effects of polypharmacy in older adults. *Clinical Pharmacology & Therapeutics, 85*(1), 86-88.

Hilmer, S. N., Gnjidic, D., & Le Couteur, D. G. (2012). Thinking through the medication list - appropriate prescribing and deprescribing in robust and frail older patients. *Aust Fam Physician, 41*(12), 924-928.

Hofer-Duckelmann, C. (2012). Gender and polypharmacotherapy in the elderly: A clinical challenge. *Sex and Gender Differences in Pharmacology, 214*, 169-182.

Hoffmann, F., van den Bussche, H., Wiese, B., Schon, G., Koller, D., Eisele, M., . . . Kaduszkiewicz, H. (2011). Impact of geriatric comorbidity and polypharmacy on cholinesterase inhibitors prescribing in dementia. *BMC Psychiatry, 11*, 190. doi:10.1186/1471-244X-11-190

Hofmann, G., & Hach, I. (2011). Prevalence of polypharmacy in older adults. *Journal of the National Comprehensive Cancer Network, 72*, 6.

Hopcroft, P., Peel, N. M., Poudel, A., Scott, I. A., Gray, L. C., & Hubbard, R. E. (2014). Prescribing for older people discharged from the acute sector to residential aged-care facilities. *Internal Medicine Journal, 44*(10), 1034-1037.

Hovstadius, B., Astrand, B., & Petersson, G. (2010). Assessment of regional variation in polypharmacy. *Pharmacoepidemiology and drug safety, 19*(4), 375-383.

Hovstadius, B., Hovstadius, K., Astrand, B., & Petersson, G. (2010). Increasing polypharmacy - an individual-based study of the Swedish population 2005-2008. *BMC Clin Pharmacol, 10*, 16. doi:10.1186/1472-6904-10-16

Hovstadius, B., & Petersson, G. (2012). Factors leading to excessive polypharmacy. *Clinics in geriatric medicine, 28*(2), 159-172.

Hovstadius, B., Petersson, G., Hellström, L., & Ericson, L. (2014). Trends in inappropriate drug therapy prescription in the elderly in Sweden from 2006 to 2013: assessment using national indicators. *Drugs & Aging, 31*(5), 379-386.

Hubbard, R. E., Peel, N. M., Scott, I. A., Martin, J. H., Smith, A., Pillans, P. I., . . . Gray, L. C. (2015). Polypharmacy among inpatients aged 70 years or older in Australia. *Medical Journal of Australia, 202*(7), 373-378.

Hudhra, K., Garcia-Caballos, M., Casado-Fernandez, E., Jucja, B., Shabani, D., & Bueno-Cavanillas, A. (2016). Polypharmacy and potentially inappropriate prescriptions identified by Beers and STOPP criteria in co-morbid older patients at hospital discharge. *Journal of Evaluation in Clinical Practice, 22*(2), 189-193.

Huiart, L., Bouhnik, A. D., Rey, D., Rousseau, F., Retornaz, F., Meresse, M., . . . Giorgi, R. (2013). Complementary or alternative medicine as possible determinant of decreased persistence to aromatase inhibitor therapy among older women with non-metastatic breast cancer. *PLoS ONE, 8*.

Hurria, A., Browner, I. S., Cohen, H. J., Denlinger, C. S., DeShazo, M., Extermann, M., . . . Wildes, T. (2012). Senior adult oncology: Clinical practice guidelines in oncology. *Journal of the National Comprehensive Cancer Network, 10*, 162-209.

Inventor, B. R. E., Henricks, J., Rodman, L., Imel, J., Holemon, L., & Hernandez, F. (2005). The impact of medical issues in inpatient geriatric psychiatry. *Issues in Mental Health Nursing 26*, 23-46.

Jensen, L. D., Andersen, O., Hallin, M., & Petersen, J. (2014). Potentially inappropriate medication related to weakness in older acute medical patients. *International Journal of Clinical Pharmacy, 36*, 570-580.

Jimenez Paez, J. M., Ariza Fuentes, L., Cardona Pelaez, A., Monterde Perez, I., & Salas Becerra, J. (2011). Multimorbility and geriatric syndromes in the residential environment. *European Geriatric Medicine, 2*, S47.

Jorgensen, T. L., Hallas, J., & Herrstedt, J. (2010). Polypharmacy in elderly cancer patients. *Journal of Clinical Oncology, 28*.

Jorgensen, T. L., Hallas, J., Land, L. H., & Herrstedt, J. (2010). Comorbidity and polypharmacy in elderly cancer patients: The significance on treatment outcome and tolerance. *Journal of Geriatric Oncology, 1*, 87-102.

Jorgensen, T. L., Herrstedt, J., Friis, S., & Hallas, J. (2012). Polypharmacy and drug use in elderly Danish cancer patients during 1996 to 2006. *Journal of Geriatric Oncology, 3*(1), 33-40.

Junius-Walker, U., Theile, G., & Hummers-Pradier, E. (2007). Prevalence and predictors of polypharmacy among older primary care patients in Germany. *Fam Pract, 24*(1), 14-19. doi:10.1093/fampra/cml067

Jyrkkä, J., Enlund, H., Korhonen, M., Sulkava, R., & Hartikainen, S. (2009). Patterns of drug use and factors associated with polypharmacy and excessive polypharmacy in elderly persons: results of the Kuopio 75+ study: a cross-sectional analysis. *Drugs & Aging, 26*(6), 493-503.

Jyrkka, J., Enlund, H., Korhonen, M. J., Sulkava, R., & Hartikainen, S. (2009). Polypharmacy status as an indicator of mortality in an elderly population. *Drugs Aging, 26*(12), 1039-1048. doi:10.2165/11319530-000000000-00000

Jyrkkä, J., Enlund, H., Lavikainen, P., Sulkava, R., & Hartikainen, S. (2011). Association of polypharmacy with nutritional status, functional ability and cognitive capacity over a three-year period in an elderly population. *Pharmacoepidemiology and drug safety, 20*(5), 514-522.

Jyrkka, J., Vartiainen, L., Hartikainen, S., Sulkava, R., & Enlund, H. (2006). Increasing use of medicines in elderly persons: a five-year follow-up of the Kuopio 75+Study. *Eur J Clin Pharmacol, 62*(2), 151-158. doi:10.1007/s00228-005-0079-6

Kenig, J., Olszewska, U., Zychiewicz, B., Barczynski, M., & Mitus-Kenig, M. (2015). Cumulative deficit model of geriatric assessment to predict the postoperative outcomes of older patients with solid abdominal cancer. *Journal of Geriatric Oncology, 6*(5), 370-379.

Klarin, I., Wimo, A., & Fastbom, J. (2005). The association of inappropriate drug use with hospitalisation and mortality: a population-based study of the very old. *Drugs & Aging, 22*(1), 69-82.

Kojima, G., Bell, C., Tamura, B., Inaba, M., Lubimir, K., Blanchette, P., . . . Masaki, K. (2012). Reducing cost by reducing polypharmacy: the polypharmacy outcomes project. *Journal of the American Medical Directors Association, 13*(9), 818.e811-818.e815.

Kojima, T., Akishita, M., Nakamura, T., Nomura, K., Ogawa, S., Iijima, K., . . . Ouchi, Y. (2012). Polypharmacy as a risk for fall occurrence in geriatric outpatients. *Geriatrics & gerontology international, 12*(3), 425-430.

Kristjansson, S. R., Jordhoy, M. S., Nesbakken, A., Skovlund, E., Bakka, A., Johannessen, H. O., & Wyller, T. B. (2010). Which elements of a comprehensive geriatric assessment (CGA) predict post-operative complications and early mortality after colorectal cancer surgery? *Journal of Geriatric Oncology, 1*, 57-65.

Kronfly Rubiano, E., Rivilla Frias, D., Ortega Abarca, I., Villanueva Villanueva, M., Beltran Martinez, E., Comellas Villalba, M., . . . Barranco Oliver, L. (2015). Risk of depression in 75 years or older persons, comprehensive geriatric assessment and associated factors in primary care: Cross sectional study. *Atencion Primaria, 47*(10), 616-625.

Kuhn-Thiel, A. M., Weis, C., Wehling, M., & members, F. a. e. p. (2014). Consensus validation of the FORTA (Fit fOR The Aged) List: a clinical tool for increasing the appropriateness of pharmacotherapy in the elderly. *Drugs & Aging, 31*(2), 131-140.

Kuijpers, M. A. J., van Marum, R. J., Egberts, A. C. G., & Jansen, P. A. F. (2008). Relationship between polypharmacy and underprescribing. *British journal of clinical pharmacology, 65*(1), 130-133.

Lai, S. W., Liao, K. F., Liao, C. C., Muo, C. H., Liu, C. S., & Sung, F. C. (2010). Polypharmacy correlates with increased risk for hip fracture in the elderly: a population-based study. *Medicine (Baltimore), 89*(5), 295-299. doi:10.1097/MD.0b013e3181f15efc

Lai, S. W., Lin, C. H., Liao, K. F., Su, L. T., Sung, F. C., & Lin, C. C. (2012). Association between polypharmacy and dementia in older people: a population-based case-control study in Taiwan. *Geriatrics & gerontology international, 12*(3), 491-498. doi:10.1111/j.1447-0594.2011.00800.x

Lai, S. W., Su, L. T., Lin, C. H., Tsai, C. H., Sung, F. C., & Hsieh, D. P. (2011). Polypharmacy increases the risk of Parkinson's disease in older people in Taiwan: a population-based study. *Psychogeriatrics, 11*(3), 150-156. doi:10.1111/j.1479-8301.2011.00369.x

Lakhanpal, R., Yoong, J., Joshi, S., Yip, D., Mileshkin, L., Marx, G. M., . . . Steer, C. B. (2015). Geriatric assessment of older patients with cancer in Australia-A multicentre audit. *Journal of Geriatric Oncology, 6*(3), 185-193.

Lalic, S., Sluggett, J. K., Ilomaki, J., Wimmer, B. C., Tan, E. C., Robson, L., . . . Bell, J. S. (2016). Polypharmacy and Medication Regimen Complexity as Risk Factors for Hospitalization Among Residents of Long-Term Care Facilities: A Prospective Cohort Study. *Journal of the American Medical Directors Association, 17*(11), 1067 e1061-1067 e1066. doi:10.1016/j.jamda.2016.08.019

Lam, D. P. Y., Mak, C. F., Chan, S. M. C., Yao, R. W. Y., Leung, S. S. Y., & You, J. H. S. (2010). Polypharmacy and inappropriate prescribing in elderly Hong Kong Chinese patients. *Journal of the American Geriatrics Society, 58*, 203-205.

Lam, M. P. S., Cheung, B. M. Y., & Wong, I. C. K. (2015). Prevalence of potentially inappropriate prescribing among Hong Kong older adults: A comparison of the Beers 2003, Beers 2012, and screening tool of older person's prescriptions and screening tool to alert doctors to right treatment criteria. *Journal of the American Geriatrics Society, 63*(7), 1471-1472.

Lapi, F., Pozzi, C., Mazzaglia, G., Ungar, A., Fumagalli, S., Marchionni, N., . . . Di Bari, M. (2009). Epidemiology of suboptimal prescribing in older, community dwellers: a two-wave, population-based survey in Dicomano, Italy. *Drugs Aging, 26*(12), 1029-1038. doi:10.2165/11319390-000000000-00000

Le Cossec C. La polymédication au regard de différents indicateurs de sa mesure: impact sur la prévalence, les classes thérapeutiques concernées et les facteurs associés. Institu de recherche et documentation en économie de la santé. Numéro 562, décembre 2015. Available: http://www.irdes.fr/recherche/rapports/562-la-polymedication-au-regard-de-differents-indicateurs-de-sa-mesure.pdf

Le Couteur, D. G., Hilmer, S. N., Glasgow, N., Naganathan, V., & Cumming, R. G. (2004). Prescribing in older people. *Aust Fam Physician, 33*(10), 777-781.

Leavy, B., Aberg, A. C., Melhus, H., Mallmin, H., Michaelsson, K., & Byberg, L. (2013). When and where do hip fractures occur? A population-based study. *Osteoporosis International, 24*, 2387-2396.

Leendertse, A. J., Egberts, A. C., Stoker, L. J., & van den Bemt, P. M. (2008). Frequency of and risk factors for preventable medication-related hospital admissions in the Netherlands. *Arch Intern Med, 168*(17), 1890-1896. doi:10.1001/archinternmed.2008.3

Lees, J. (2009). Polypharmacy in the older cancer patient - The role of the pharmacist in the team. *Asia-Pacific Journal of Clinical Oncology, 5*, A156.

Lees, J. (2013). *Management of polypharmacy in older cancer patients.* Paper presented at the Cancer Forum.

Lees, J., & Chan, A. (2011). Polypharmacy in elderly patients with cancer: clinical implications and management. *The Lancet Oncology 12*(13), 1249-1257. doi:10.1016/S1470-2045(11)70040-7

Leiss, W., Méan, M., Limacher, A., Righini, M., Jaeger, K., Beer, H.-J., . . . Aujesky, D. (2015). Polypharmacy is associated with an increased risk of bleeding in elderly patients with venous thromboembolism. *Journal of General Internal Medicine, 30*(1), 17-24.

Lichtman, S., & Boparai, M. (2008). Anticancer drug therapy in the older cancer patient: pharmacology and polypharmacy. *Current Treatment Options in Oncology, 9*(2-3), 191-203.

Linnebur, S. A., Vande Griend, J. P., Hirsch, J. D., Metz, K. R., Hosokawa, P. W., & Libby, A. M. (2014). Patient-level medication regimen complexity in older adults with depression. *Journal of the American Geriatrics Society, 62*, S60.

Liu, C.-p. B., Leung, D. S.-Y., & Chi, I. (2011). Social functioning, polypharmacy and depression in older Chinese primary care patients. *Aging & mental health, 15*(6), 732-741.

Loffler, C. (2014). Optimizing polypharmacy among elderly hospital patients with chronic diseases--study protocol of the cluster randomized controlled POLITE-RCT trial. *Implementation science, 9*, 151.

López Molina, T., Caraballo Camacho, M., Palma Morgado, D., López Rubio, S., Domínguez Camacho, J., & Morales Serna, J. (2012). [Prevalence of multiple medication and cardiovascular risk in patients over 65 years]. *Atencion primaria, 44*(4), 216-222.

Lowthian, J., Straney, L. D., Brand, C. A., Barker, A. L., De Villiers Smit, P., Newnham, H., . . . Cameron, P. A. (2016). Unplanned early return to the emergency department by older patients: The Safe Elderly Emergency Department Discharge (SEED) project. *Age and Ageing, 45*(2), 255-261.

Lu, W. H., Wen, Y. W., Chen, L. K., & Hsiao, F. Y. (2015). Effect of polypharmacy, potentially inappropriate medications and anticholinergic burden on clinical outcomes: A retrospective cohort study. *Canadian Medical Association Journal, 187*(4), E130-E137.

Luzny, J., Bellova, J., & Donek, E. (2010). Does gerontopsychiatry belong to medicine? Cross-sectional study monitoring polymorbidity in hospitalized gerontopsychiatric patients. *Biomed Pap Med Fac Univ Palacky Olomouc Czech Repub, 154*(1), 89-92.

Maggiore, R. J., Dale, W., Gross, C. P., Feng, T., Tew, W. P., Mohile, S. G., . . . Hurria, A. (2014). Polypharmacy and potentially inappropriate medication use in older adults with cancer undergoing chemotherapy: Effect on chemotherapy-related toxicity and hospitalization during treatment. *Journal of the American Geriatrics Society, 62*, 1505-1512.

Maggiore, R. J., Gross, C. P., & Hurria, A. (2010). Polypharmacy in older adults with cancer. *Oncologist, 15*(5), 507-522. doi:10.1634/theoncologist.2009-0290

Maher, R. L., Hanlon, J., & Hajjar, E. R. (2014). Clinical consequences of polypharmacy in elderly. *Expert Opin Drug Saf, 13*(1), 57-65. doi:10.1517/14740338.2013.827660

Mamun, K., Lien, C. T., Goh-Tan, C. Y., & Ang, W. S. (2004). Polypharmacy and inappropriate medication use in Singapore nursing homes. *Ann Acad Med Singapore, 33*(1), 49-52.

Mannucci, P. M., & Nobili, A. (2014). Multimorbidity and polypharmacy in the elderly: lessons from REPOSI. *Intern Emerg Med, 9*(7), 723-734. doi:10.1007/s11739-014-1124-1

Mansur, N., Weiss, A., & Beloosesky, Y. (2008). Relationship of in-hospital medication modifications of elderly patients to postdischarge medications, adherence, and mortality. *Annals of Pharmacotherapy, 42*(6), 783-789. doi:10.1345/aph.1L070

Maraldi, C., Lattanzio, F., Onder, G., Gallerani, M., Bustacchini, S., De Tommaso, G., & Volpato, S. (2009). Variability in the prescription of cardiovascular medications in older patients: correlates and potential explanations. *Drugs Aging, 26 Suppl 1*, 41-51. doi:10.2165/11534650-000000000-00000

Marcum, Z. A., Amuan, M. E., Hanlon, J. T., Aspinall, S. L., Handler, S. M., Ruby, C. M., & Pugh, M. J. (2012). Prevalence of unplanned hospitalizations caused by adverse drug reactions in older veterans. *Journal of the American Geriatrics Society, 60*(1), 34-41. doi:10.1111/j.1532-5415.2011.03772.x

Marcum, Z. A., & Gellad, W. F. (2012). Medication Adherence to Multidrug Regimens. *Clinics in Geriatric Medicine, 28*, 287-300.

Marshall, F. J. (2012). Approach to the elderly patient with gait disturbance. *Neurology: Clinical Practice, 2*, 103-111.

Martens P, Fransoo R, *The Need to Know Team,* Burland E, Prior H, Burchill C, Romphg L, et al. What works? A first look at evaluating Manitoba's regional health programs and policies at the population level. Winnipeg, Manitoba Centre for Health Policy, March 2008.

Martinez-Selles, M., Gomez Huelgas, R., bu-Assi, E., Calderon, A., & Vidan, M. T. (2016). Chronic ischaemic heart disease in the elderly. *Medicina Clinica, 146*(8), 372.

Mastromarino, V., Casenghi, M., Testa, M., Gabriele, E., Coluccia, R., Rubattu, S., & Volpe, M. (2014). Polypharmacy in heart failure patients. *Current Heart Failure Reports, 11*, 212-219.

Mazzone, A., Bo, M., Lucenti, A., Galimberti, S., Bellelli, G., & Annoni, G. (2016). The role of comprehensive geriatric assessment and functional status in evaluating the patterns of antithrombotic use among older people with atrial fibrillation. *Archives of Gerontology and Geriatrics, 65*, 248-254.

McGory, M. L., Shekelle, P. G., Rubenstein, L. Z., Fink, A., & Ko, C. Y. (2005). Developing quality indicators for elderly patients undergoing abdominal operations. *J Am Coll Surg, 201*(6), 870-883. doi:10.1016/j.jamcollsurg.2005.07.009

McMahon, C. G., Cahir, C. A., Kenny, R. A., & Bennett, K. (2014). Inappropriate prescribing in older fallers presenting to an Irish emergency department. *Age and Ageing, 43*, 44-50.

Meloni, E., Vetrano, D. L., Bernabei, R., & Onder, G. (2015). Appropriateness of pharmacological treatment in older people with dementia. *Italian Journal of Medicine, 9*(3), 212-216.

Min, L., Wenger, N., Walling, A. M., Blaum, C., Cigolle, C., Ganz, D. A., . . . Kerr, E. A. (2013). When comorbidity, aging, and complexity of primary care meet: Development and validation of the geriatric complexity of care index. *Journal of the American Geriatrics Society, 61*, 542-550.

Mira, J. J., Orozco-Beltran, D., Perez-Jover, V., Martinez-Jimeno, L., Gil-Guillen, V. F., Carratala-Munuera, C., . . . Asencio-Aznar, A. (2013). Physician patient communication failure facilitates medication errors in older polymedicated patients with multiple comorbidities. *Family Practice, 30*(1), 56-63. doi:10.1093/fampra/cms046

Mirk, A., Kemp, L., Echt, K. V., & Perkins, M. M. (2013). Integrated management and polypharmacy review of vulnerable elders: Can we improve outcomes? *Journal of the American Geriatrics Society, 61*, S92.

Mizokami, F., Koide, Y., Noro, T., & Furuta, K. (2012). Polypharmacy with common diseases in hospitalized elderly patients. *Am J Geriatr Pharmacother, 10*(2), 123-128. doi:10.1016/j.amjopharm.2012.02.003

Mizokami, F., Mase, H., Kinoshita, T., Kumagai, T., Furuta, K., & Ito, K. (2016). Adherence to Medication Regimens is an Effective Indicator of Cognitive Dysfunction in Elderly Individuals. *American Journal of Alzheimer's Disease and other Dementias, 31*(2), 132-136.

Modig, S., Kristensson, J., Ekwall, A. K., Hallberg, I. R., & Midlov, P. (2009). Frail elderly patients in primary care--their medication knowledge and beliefs about prescribed medicines. *European Journal of Clinical Pharmacology, 65*(2), 151-155. doi:10.1007/s00228-008-0581-8

Monégat M, Sermet C, Perronnin M, Rococo E. La polymédication: définitions, mesures et enjeux. Questions d'économie de la santé, numéro 204, décembre 2014. Available: http://www.irdes.fr/recherche/questions-d-economie-de-lasante/ 204-la-polymedication-defi nitions-mesures-et-enjeux.pdf

Montero-Balosa, M. C., Palma-Morgado, D., Ma, J. L.-A., Real, F. S. D., Sagrista-Gonzalez, M., & Fernandez-Urrusuno, R. (2015). Effectiveness of medication review in patients with polypharmacy. *International Journal of Clinical Pharmacy, 37*(1), 277-278.

Morandi, A., Bellelli, G., Vasilevskis, E. E., Turco, R., Guerini, F., Torpilliesi, T., . . . Trabucchi, M. (2013). Predictors of rehospitalization among elderly patients admitted to a rehabilitation hospital: The role of polypharmacy, functional status, and length of stay. *Journal of the American Medical Directors Association, 14*, 761-767.

Moriarty F, Hardy C, Bennett K, Smith S, Fahey T. Trends and interaction of polypharmacy and potentially inappropriate prescribing in primary care over 15 years in Ireland: a repeated cross-sectional study. BMJ Open 2015;5:e008656.

Moulis, F., Moulis, G., Balardy, L., Gerard, S., Sourdet, S., Rouge-Bugat, M. E., . . . Vellas, B. (2015). Searching for a polypharmacy threshold associated with frailty. *Journal of the American Medical Directors Association, 16*(3), 258-261.

Munger, M. A. (2010). Polypharmacy and combination therapy in the management of hypertension in elderly patients with co-morbid diabetes mellitus. *Drugs Aging, 27*(11), 871-883. doi:10.2165/11538650-000000000-00000

Naveiro-Rilo, J. C., ez-Juarez, D., Flores-Zurutuza, M. L., Javierre Perez, P., berte Perez, C., & Molina Mazo, R. (2014). [Quality of life in the elderly on polymedication and with multiple morbidities]. *Revista Espanola de Geriatria y Gerontologia, 49*(4), 158-164.

Neuner-Jehle, S. (2013). [Less is more - how to prevent polypharmacy?]. *Praxis (Bern 1994), 102*(1), 21-27. doi:10.1024/1661-8157/a001161

Nguyen, J. K., Fouts, M. M., Kotabe, S. E., & Lo, E. (2006). Polypharmacy as a risk factor for adverse drug reactions in geriatric nursing home residents. *Am J Geriatr Pharmacother, 4*(1), 36-41. doi:10.1016/j.amjopharm.2006.03.002

NHS Highland (2015). Polypharmacy: Guidance for Prescribing in frail adults. Available : https://www.nhshighland.scot.nhs.uk/Publications/Documents/Guidelines/Polypharmacy%20Guidance%20for%20Prescribing%20in%20Frail%20Adults.pdf (accessed September 13, 2016).

Nightingale, G., Hajjar, E., Guo, K., Komura, S., Urnoski, E., Sendecki, J., . . . Chapman, A. (2015). A pharmacist-led medication assessment used to determine a more precise estimation of the prevalence of complementary and alternative medication (CAM) use among ambulatory senior adults with cancer. *Journal of Geriatric Oncology, 6*(5), 411-417.

Nightingale, G., Hajjar, E., Swartz, K., ndrel-Sendecki, J., & Chapman, A. (2015). Evaluation of a pharmacist-led medication assessment used to identify prevalence of and associations with polypharmacy and potentially inappropriate medication use among ambulatory senior adults with cancer. *Journal of Clinical Oncology, 33*(13), 1453-1459.

Noale, M., Veronese, N., Cavallo Perin, P., Pilotto, A., Tiengo, A., Crepaldi, G., & Maggi, S. (2016). Polypharmacy in elderly patients with type 2 diabetes receiving oral antidiabetic treatment. *Acta Diabetologica, 53*(2), 323-330.

Nobili, A., Franchi, C., Pasina, L., Tettamanti, M., Baviera, M., Monesi, L., . . . Merlino, L. (2011). Drug utilization and polypharmacy in an Italian elderly population: the EPIFARM-elderly project. *Pharmacoepidemiology and drug safety, 20*(5), 488-496. doi:10.1002/pds.2108

Nobili, A., Licata, G., Salerno, F., Pasina, L., Tettamanti, M., Franchi, C., . . . Mannucci, P. M. (2011). Polypharmacy, length of hospital stay, and in-hospital mortality among elderly patients in internal medicine wards. The REPOSI study. *European Journal of Clinical Pharmacology, 67*, 507-519.

Nobili, A., Marengoni, A., Tettamanti, M., Salerno, F., Pasina, L., Franchi, C., . . . Mannucci, P. M. (2011). Association between clusters of diseases and polypharmacy in hospitalized elderly patients: results from the REPOSI study. *Eur J Intern Med, 22*(6), 597-602. doi:10.1016/j.ejim.2011.08.029

Nomura, K. (2011). Drug use patterns and predictors of polypharmacy among elderly, community-residing persons in Hiroshima, Japan from October to December 2009. *Liverpool, UK: Laureate Online Education*.

Nomura, K., & Akazawa, M. (2012). Overview of drug use among the elderly in Japan using IMS prescription database. *Pharmacoepidemiology and Drug Safety, 21*, 80.

O'Sullivan, D. P., O'Mahony, D., Parsons, C., Hughes, C., Murphy, K., Patterson, S., & Byrne, S. (2013). A prevalence study of potentially inappropriate prescribing in Irish long-term care residents. *Drugs Aging, 30*(1), 39-49. doi:10.1007/s40266-012-0039-7

Oh, E. S., Li, M., Fafowora, T. M., Inouye, S. K., Chen, C. H., Rosman, L. M., . . . Puhan, M. A. (2015). Preoperative risk factors for postoperative delirium following hip fracture repair: A systematic review. *International Journal of Geriatric Psychiatry, 30*(9), 900-910.

Olsson, I. N., Runnamo, R., & Engfeldt, P. (2011). Medication quality and quality of life in the elderly, a cohort study. *Health Qual Life Outcomes, 9*(1), 95. doi:10.1186/1477-7525-9-95

Onder, G., Liperoti, R., Fialova, D., Topinkova, E., Tosato, M., Danese, P., . . . Landi, F. (2012). Polypharmacy in nursing home in Europe: results from the SHELTER study. *J Gerontol A Biol Sci Med Sci, 67*(6), 698-704. doi:10.1093/gerona/glr233

Onder, G., Liperoti, R., Foebel, A., Fialova, D., Topinkova, E., van der Roest, H. G., . . . project, S. (2013). Polypharmacy and mortality among nursing home residents with advanced cognitive impairment: results from the SHELTER study. *Journal of the American Medical Directors Association, 14*(6), 450 e457-412. doi:10.1016/j.jamda.2013.03.014

Orfila, F., Garrofe, C., Tajada, C., Pavon, I. L., & Cegri, F. (2014). Frailty and the risk of confinement, institutionalization or death in an elderly cohort. *European Journal of General Practice, 20*(3), 227.

Pan, H.-H., Li, C.-Y., Chen, T.-J., Su, T.-P., & Wang, K.-Y. (2014). Association of polypharmacy with fall-related fractures in older Taiwanese people: age- and gender-specific analyses. *BMJ Open, 4*(3), e004428-e004428.

Parian, A., & Ha, C. (2015). Older Age and Steroid Use Are Associated with Increasing Polypharmacy and Potential Medication Interactions Among Patients with Inflammatory Bowel Disease. *Inflamm Bowel Dis, 21*(6), 1392-1400.

Park, J. W., Roh, J. L., Lee, S. W., Kim, S. B., Choi, S. H., Nam, S. Y., & Kim, S. Y. (2016). Effect of polypharmacy and potentially inappropriate medications on treatment and posttreatment courses in elderly patients with head and neck cancer. *Journal of Cancer Research and Clinical Oncology, 142*(5), 1031-1040.

Patterson, S. M., Cadogan, C. A., Kerse, N., Cardwell, C. R., Bradley, M. C., Ryan, C., & Hughes, C. (2014). Interventions to improve the appropriate use of polypharmacy for older people. *Cochrane Database Syst Rev*(10), CD008165. doi:10.1002/14651858.CD008165.pub3

Payne, R. A., & Avery, A. J. (2011). Polypharmacy: one of the greatest prescribing challenges in general practice. *Br J Gen Pract, 61*(583), 83-84. doi:10.3399/bjgp11X556146

Pedone, C., Ercolani, S., Catani, M., Maggio, D., Ruggiero, C., Quartesan, R., . . . Cherubini, A. (2005). Elderly patients with cognitive impairment have a high risk for functional decline during hospitalization: The GIFA study. *Journals of Gerontology - Series A Biological Sciences and Medical Sciences, 60*, 1576-1580.

Pervin, L. (2008). Polypharmacy and the aging: is there cause for concern. *ARN Network, 25*(1), 6-7.

Pimenta, E., & Oparil, S. (2012). Management of hypertension in the elderly. *Nature Reviews Cardiology, 9*, 286-296.

Pire, V., Fournier, A., Schoevaerdts, D., Spinewine, A., & Swine, C. (2009). Polypharmacy among the elderly. *Louvain Medical, 128*, 235-240.

Poudel, A., Peel, N. M., Mitchell, C. A., Gray, L. C., Nissen, L. M., & Hubbard, R. E. (2015). Geriatrician interventions on medication prescribing for frail older people in residential aged care facilities. *Clin Interv Aging, 10*, 1043-1051.

Pozzi, C., Lapi, F., Mazzaglia, G., Inzitari, M., Boncinelli, M., Geppetti, P., . . . Di Bari, M. (2010). Is suboptimal prescribing a risk factor for poor health outcomes in community-dwelling elders? The ICARe Dicomano study. *Pharmacoepidemiology and drug safety, 19*(9), 954-960.

Prithviraj, G. K., Bagai, R., Koroukian, S. M., Berger, N. A., & Owusu, C. (2010). Polypharmacy and functional status in older patients with breast, colon, and lung cancers. *Journal of Clinical Oncology, 28*.

Prithviraj, G. K., Koroukian, S., Margevicius, S., Berger, N. A., Bagai, R., & Owusu, C. (2012). Patient characteristics associated with polypharmacy and inappropriate prescribing of medications among older adults with cancer. *Journal of Geriatric Oncology, 3*, 228-237.

Pugh, J. A., Wang, C. P., Espinoza, S. E., Noel, P. H., Bollinger, M., Amuan, M., . . . Pugh, M. J. (2014). Influence of frailty-related diagnoses, high-risk prescribing in elderly adults, and primary care use on readmissions in fewer than 30 days for veterans aged 65 and older. *Journal of the American Geriatrics Society, 62*(2), 291-298. doi:10.1111/jgs.12656

Pugh, M. J. V., Palmer, R. F., Parchman, M. L., Mortensen, E., Markides, K., & Espino, D. V. (2007). Association of suboptimal prescribing and change in lower extremity physical function over time. *Gerontology, 53*, 445-453.

Puts, M. T. E., Costa-Lima, B., Monette, J., Girre, V., Wolfson, C., Batist, G., & Bergman, H. (2009). Medication Problems in Older, Newly Diagnosed Cancer Patients in Canada: How Common are They? *Drugs & Aging, 26*(6), 519-536.

Ramage-Morin PL. (2009)  Medication use among senior Canadians. Statistics Canada, Catalogue no. 82-003-XPE Health Reports, 20(1):1-8

Ramirez-Duque, N., Rivas-Cobas, C., Bernabeu-Wittel, M., Ruiz-Cantero, A., Murcia-Zaragoza, J., Oliver, M., & ez-Manglano, J. (2014). Drug prescription profile in patients with advanced chronic diseases. *Revista Espanola de Geriatria y Gerontologia, 49*(6), 255-259.

Reason, B., Terner, M., Moses McKeag, A., Tipper, B., & Webster, G. (2012). The impact of polypharmacy on the health of Canadian seniors. *Family Practice, 29*, 427-432.

Reboredo-Garcia, S., Mateo, C. G. C., & Casal-Llorente, C. (2014). Implantation of a program for polymedicated patients within the framework of the Galician Strategy for Integrated Chronic Care. *Atencion Primaria, 46*(S3), 33-40.

Reddy, P., Gosavi, D., & Varma, S. K. (2012). An overview of geriatric pharmacology. *Asian Journal of Pharmaceutical and Clinical Research, 5*, 25-29.

Regroupement des Unités de courte durée gériatriques et des services hospitaliers de gériatrie du Québec (RUSHGQ). (2015). Guide de gestion médicamenteuse en UCDG. Sous-comité sur la médication. 39 pages

Riccio, D., Solinas, A., Astara, G., & Mantovani, G. (2007). Comprehensive geriatric assessment in female elderly patients with alzheimer disease and other types of dementia. *Archives of Gerontology and Geriatrics, 44*, 343-353.

Rich, M. W. (2014). Secondary prevention of cardiovascular disease in older adults. *Progress in Cardiovascular Diseases, 57*(2), 168-175.

Richardson, K., Ananou, A., Lafortune, L., Brayne, C., & Matthews, F. E. (2011). Variation over time in the association between polypharmacy and mortality in the older population. *Drugs Aging, 28*(7), 547-560. doi:10.2165/11592000-000000000-00000

Riker, G. I., & Setter, S. M. (2012). Polypharmacy in older adults at home: what it is and what to do about it--implications for home healthcare and hospice. *Home Healthcare Nurse, 30*(8), 474-485; quiz 486-477. doi:10.1097/NHH.0b013e31826502dd

Rohrer, J. E., Garrison, G., Oberhelman, S. A., & Meunier, M. R. (2013). Epidemiology of polypharmacy among family medicine patients at hospital discharge. *Journal of Primary Care & Community Health, 4*(2), 101-105. doi:10.1177/2150131912472905

Romana, A., Kamath, L., Sarda, A., Muraraiah, S., & Jayanthi, C. R. (2012). Polypharmacy leading to adverse drug reactions in elderly in a tertiary care hospital. *International Journal of Pharma and Bio Sciences, 3*, P218-P224.

Rozenfeld, S., Fonseca, M. J. M., & Acurcio, F. A. (2008). Drug utilization and polypharmacy among the elderly: a survey in Rio de Janeiro City, Brazil. *Uso de medica memos y polifarmacia en adultos mayores:encuesta en la ciudad de Rio de Janeiro, Brasil., 23*(1), 34-43.

Ruberu, R. P., & Fitzgerald, S. P. (2012). Clinical Practice Guidelines for Chronic Diseases-Understanding and Managing Their Contribution to Polypharmacy. *Clinics in Geriatric Medicine, 28*, 187-198.

Ruggiero, C., Dell'Aquila, G., Gasperini, B., Onder, G., Lattanzio, F., Volpato, S., . . . Group, U. S. (2010). Potentially inappropriate drug prescriptions and risk of hospitalization among older, Italian, nursing home residents: the ULISSE project. *Drugs Aging, 27*(9), 747-758. doi:10.2165/11538240-000000000-00000

Runganga, M., Peel, N. M., & Hubbard, R. E. (2014). Multiple medication use in older patients in post-acute transitional care: a prospective cohort study. *Clin Interv Aging, 9*, 1453-1462. doi:10.2147/CIA.S64105

Saarelainen, L. K., Turner, J. P., Shakib, S., Singhal, N., Hogan-Doran, J., Prowse, R., . . . Bell, J. S. (2014). Potentially inappropriate medication use in older people with cancer: Prevalence and correlates. *Journal of Geriatric Oncology, 5*, 439-446.

Salahudeen, M. S. H., S. N.; Nishtala, P. S. (2015). Comparison of anticholinergic risk scales and associations with adverse health outcomes in older people. *Journal of the American Geriatrics Society, 63*(1), 85-90.

Salazar, J. A., Poon, I., & Nair, M. (2007). Clinical consequences of polypharmacy in elderly: expect the unexpected, think the unthinkable. *Expert Opin Drug Saf, 6*(6), 695-704. doi:10.1517/14740338.6.6.695

Salwe, K. J., Kalyansundaram, D., & Bahurupi, Y. (2016). A Study on Polypharmacy and Potential Drug-Drug Interactions among Elderly Patients Admitted in Department of Medicine of a Tertiary Care Hospital in Puducherry. *Journal of Clinical and Diagnostic Research, 10*(2), FC06-10. doi:10.7860/JCDR/2016/16284.7273

Sanglier, T., Saragoussi, D., Milea, D., & Tournier, M. (2015). Depressed older adults may be less cared for than depressed younger ones. *Psychiatry Research, 229*(3), 905-912.

Santibáñez Beltrán, S., Villarreal Ríos, E., Galicia Rodríguez, L., Martínez González, L., Vargas Daza, E., & Ramos López, J. (2013). [Economic cost of polypharmacy in the elderly in primary health care]. *Revista Medica del Instituto Mexicano del Seguro Social, 51*(2), 192-199.

Sato, I., & Akazawa, M. (2013). Polypharmacy and adverse drug reactions in Japanese elderly taking antihypertensives: a retrospective database study. *Drug Healthc Patient Saf, 5*, 143-150. doi:10.2147/DHPS.S45347

Scheidt-Nave, C., Fuchs, J., Du, Y., Ellert, U., Schienkiewitz, A., Busch, M., . . . Bode, L. (2013). Addressing health and ageing in the German national health monitoring system. *BMC Proceedings, 7*.

Schonenberger, T., Rauber, S., Hauser, N., Roth, M., Wettstein, A., & Schonenberger, P. M. (2011). [Polypharmacy is an indicator for a poor prognosis, which is not altered by deprescribing in nursing home]. *Praxis (Bern 1994), 100*(4), 213-220. doi:10.1024/1661-8157/a000431

Schrijver, E. J., Toppinga, Q., de Vries, O. J., Kramer, M. H., & Nanayakkara, P. W. (2013). An observational cohort study on geriatric patient profile in an emergency department in the Netherlands. *Neth J Med, 71*(6), 324-330.

Schuler, J., Duckelmann, C., Beindl, W., Prinz, E., Michalski, T., & Pichler, M. (2008). Polypharmacy and inappropriate prescribing in elderly internal-medicine patients in Austria. *Wiener Klinische Wochenschrift, 120*, 733-741.

Scott, IA, Gray LC, Martin JH, Mitchell CA. (2012) Minimizing inappropriate medications in older populations: A 10-step conceptual framework. Am J Med 125(6): 529-37.

Scott, I., Hilmer, S., Reeve, E., Potter, K., Le Couteur, D., Rigby, D., . . . Martin, J. (2015). Reducing inappropriate polypharmacy: the process of deprescribing. *JAMA Intern Med, 175*(5), 827-834.

Scotttish Governement Model of Care Polypharmacy Working Group. Polypharmacy Guidance ($2^{nd}$ edition). March 2015. Scottish Government

Segal, A. R., Staum, E. L., & Munshi, M. (2009). Polypharmacy is associated with frequent hypoglycemia, cognitive dysfunction, and poor nutritional status in older adults with diabetes. *Diabetes Research & Clinical Practice, 58*.

Sehgal, V., Bajwa, S. J. S., Sehgal, R., Bajaj, A., Khaira, U., & Kresse, V. (2013). Polypharmacy and potentially inappropriate medication use as the precipitating factor in readmissions to the hospital. *Journal of Family Medicine and Primary Care, 2*(2), 194-199.

Sengstock, D., & Zimmerman, J. (2014). Addressing Polypharmacy & Improving Medication Adherence in Older Adults. In B. A. Williams, A. Chang, C. Ahalt, H. Chen, R. Conant, C. S. Landefeld, C. Ritchie, & M. Yukawa (Eds.), *Current Diagnosis & Treatment: Geriatrics, 2e*. New York, NY: McGraw-Hill Education.

Sergi, G., De Rui, M., Sarti, S., & Manzato, E. (2011). Polypharmacy in the elderly: can comprehensive geriatric assessment reduce inappropriate medication use? *Drugs Aging, 28*(7), 509-518. doi:10.2165/11592010-000000000-00000

Sganga, F., Landi, F., Ruggiero, C., Corsonello, A., Vetrano, D. L., Lattanzio, F., . . . Onder, G. (2015). Polypharmacy and health outcomes among older adults discharged from hospital: results from the CRIME study. *Geriatrics & gerontology international, 15*(2), 141-146.

Sganga, F., Vetrano, D. L., Volpato, S., Cherubini, A., Ruggiero, C., Corsonello, A., . . . Onder, G. (2014). Physical performance measures and polypharmacy among hospitalized older adults: results from the CRIME study. *The Journal of Nutrition, Health & Aging, 18*(6), 616-621.

Shah, B. M., & Hajjar, E. R. (2012). Polypharmacy, Adverse Drug Reactions, and Geriatric Syndromes. *Clinics in Geriatric Medicine, 28*, 173-186.

Sharifi, H., Hasanloei, M. A., & Mahmoudi, J. (2014). Polypharmacy-induced Drug-Drug Interactions; Threats to Patient Safety. *Drug Research (Stuttg), 64*(12), 633-637. doi:10.1055/s-0033-1363965

Shastry, R. I., Ullal Sheetal, D., Sowjanya, Bethi, Y., & Kumar, J. U. (2015). Cost analysis of drugs used in elderly patients with cardiovascular disorder. *Research Journal of Pharmaceutical, Biological and Chemical Sciences, 6*(1), 83-86.

Shimizu, K., Ishii, S., Tanaka, T., Shibasaki, K., Akishita, M., & Iijima, K. (2014). Use of potentially inappropriate medication and polypharmacy in community-dwelling japanese elderly population from the kashiwa study. *Journal of the American Geriatrics Society, 62*, S249-S250.

Silvestre, L. C.-S., P.; Oliveira, P.; Carneiro, A. I.; Fernandes, A. I.; Costa, F. A. (2015). Identification of drug related problems in a sample of Portuguese nursing homes. *International Journal of Clinical Pharmacy, 37*(2), 410-411.

Singler, K., Thiem, U., Christ, M., Zenk, P., Biber, R., Sieber, C. C., & Heppner, H. J. (2014). Aspects and assessment of delirium in old age. First data from a German interdisciplinary emergency department. *Z Gerontol Geriatr, 47*(8), 680-685.

Skoldunger, A., Fastbom, J., Wimo, A., Fratiglioni, L., & Johnell, K. (2015). Impact of Inappropriate Drug Use on Hospitalizations, Mortality, and Costs in Older Persons and Persons with Dementia: Findings from the SNAC Study. *Drugs and Aging, 32*(8), 671-678.

Slabaugh, S. L., Maio, V., Templin, M., & Abouzaid, S. (2010). Prevalence and risk of polypharmacy among the elderly in an outpatient setting: a retrospective cohort study in the Emilia-Romagna region, Italy. *Drugs & Aging, 27*(12), 1019-1028.

Sokol, K. C., Knudsen, J. F., & Li, M. M. (2007). Polypharmacy in older oncology patients and the need for an interdisciplinary approach to side-effect management. *Journal of Clinical Pharmacy and Therapeutics, 32*(2), 169-175. doi:10.1111/j.1365-2710.2007.00815.x

Soleymani, F. (2010). Drug use pattern in over 60-year-old population of Tehran in 2007 Iran. *Value in Health, 13*, A534.

Sonnichsen, A., Trampisch, U. S., Rieckert, A., Piccoliori, G., Vogele, A., Flamm, M., . . . Kunnamo, I. (2016). Polypharmacy in chronic diseases-Reduction of Inappropriate Medication and Adverse drug events in older populations by electronic Decision Support (PRIMA-eDS): Study protocol for a randomized controlled trial. *Trials, 17*(1), 57.

Srinivasan, A., Ambler, G. K., Hayes, P. D., Chowdhury, M. M., Ashcroft, S., Boyle, J. R., & Coughlin, P. A. (2016). Premorbid function, comorbidity, and frailty predict outcomes after ruptured abdominal aortic aneurysm repair. *Journal of Vascular Surgery, 63*(3), 603-609. doi:10.1016/j.jvs.2015.09.002

Stauder, R., Spross, J. M., & Augscholl, J. (2015). Geriatric assessment defines impairments and clinical outcome in myelodysplastic syndromes. *Leukemia Research, 39*, S65.

Stawicki, S. P., & Gerlach, A. T. (2009). Polypharmacy and medication errors: Stop, listen, look, and analyze. *Opus, 12*, 6-10.
Steinman, M. A. (2007). Polypharmacy and the balance of medication benefits and risks. *Am J Geriatr Pharmacother, 5*(4), 314-316. doi:10.1016/j.amjopharm.2007.12.009
Steinman, M. A., Landefeld, C. S., Rosenthal, G. E., Berthenthal, D., Sen, S., & Kaboli, P. J. (2006). Polypharmacy and prescribing quality in older people. *Journal of the American Geriatrics Society, 54*(10), 1516-1523. doi:10.1111/j.1532-5415.2006.00889.x
Strehblow, C., Smeikal, M., & Fasching, P. (2014). Polypharmacy and excessive polypharmacy in octogenarians and older acutely hospitalized patients. *Wiener Klinische Wochenschrift, 126*(7-8), 195-200. doi:10.1007/s00508-013-0485-1
Sumukadas, D., McMurdo, M. E., Mangoni, A. A., & Guthrie, B. (2014). Temporal trends in anticholinergic medication prescription in older people: repeated cross-sectional analysis of population prescribing data. *Age Ageing, 43*(4), 515-521. doi:10.1093/ageing/aft199
Tam-McDevitt, J. (2008). Polypharmacy, aging, and cancer. *Oncology (Williston Park), 22*, 1052-1055.
Tan, C. B., Ng, J., Jeganathan, R., Kawai, F., Pan, C. X., Pollock, S., . . . Chorost, M. (2015). Cognitive changes after surgery in the elderly: Does minimally invasive surgery influence the incidence of postoperative cognitive changes compared to open colon surgery? *Dementia and Geriatric Cognitive Disorders, 39*(3-4), 125-131.
Thai, M., Hilmer, S. N., Pearson, S. A., Reeve, E., & Gnjidic, D. (2015). Prevalence of Potential and Clinically Relevant Statin-Drug Interactions in Frail and Robust Older Inpatients. *Drugs & Aging, 32*(10), 849-856.
Thurmann, P. A. (2013). [Less can be more - drug therapy in the elderly]. *Z Evid Fortbild Qual Gesundhwes, 107*(2), 148-152. doi:10.1016/j.zefq.2013.02.007
Torrance, A. D. W., Powell, S. L., & Griffiths, E. A. (2015). Emergency surgery in the elderly: Challenges and solutions. *Open Access Emergency Medicine, 7*, 55-68.
Tulner, L. R., Frankfort, S. V., Wesselius, F., van Campen, J. P. C. M., Koks, C. H. W., & Beijnen, J. H. (2009). Do geriatric outpatients adhere to medication changes advised after assessment? An exploratory pilot study. *Current Clinical Pharmacology, 4*, 154-158.
Tulner, L. R., Van Campen, J. P. C. M., Frankfort, S. V., Koks, C. H. W., Beijnen, J. H., Brandjes, D. P. M., & Jansen, P. A. F. (2010). Changes in under-treatment after comprehensive geriatric assessment: An observational study. *Drugs and Aging, 27*, 831-843.
Turner, J. P., Jamsen, K. M., Shakib, S., Singhal, N., Prowse, R., & Bell, J. S. (2016). Polypharmacy cut-points in older people with cancer: how many medications are too many? *Supportive Care in Cancer, 24*(4), 1831-1840.
Turner, J. P., Shakib, S., Singhal, N., Hogan-Doran, J., Prowse, R., Johns, S., & Bell, J. S. (2014). Prevalence and factors associated with polypharmacy in older people with cancer. *Supportive care in cancer : official journal of the Multinational Association of Supportive Care in Cancer, 22*, 1727-1734.
Ussai, S., Casetta, A., Bresigar, M., Barbone, F., & Pisa, F. (2013). General physician therapy review and polypharmacy in elderly outpatients. *Drug Safety, 36*, 863.

van der Marck, M. A., Klok, M. P., Okun, M. S., Giladi, N., Munneke, M., Bloem, B. R., . . . Wood, B. H. (2014). Consensus-based clinical practice recommendations for the examination and management of falls in patients with Parkinson's disease. *Parkinsonism and Related Disorders, 20*, 360-369.

Vande Walle, N., Kenis, C., Heeren, P., Van Puyvelde, K., Decoster, L., Beyer, I., . . . Milisen, K. (2014). Fall predictors in older cancer patients: a multicenter prospective study. *BMC geriatrics, 14*, 135.

Vass, M., & Hendriksen, C. (2005). Polypharmacy and older people-the GP perspective. *Zeitschrift für Gerontologie und Geriatrie, 38 Suppl 1*, I14-17. doi:10.1007/s00391-005-1104-1

Venturini, C. D., Engroff, P., Ely, L. S., Zago, L. F., Schroeter, G., Gomes, I., . . . Morrone, F. B. (2011). Gender differences, polypharmacy, and potential pharmacological interactions in the elderly. *Clinics (Sao Paulo), 66*(11), 1867-1872.

Vetrano, D. L., Tosato, M., Colloca, G., Topinkova, E., Fialova, D., Gindin, J., . . . Onder, G. (2013). Polypharmacy in nursing home residents with severe cognitive impairment: results from the SHELTER Study. *Alzheimers Dement, 9*(5), 587-593. doi:10.1016/j.jalz.2012.09.009

Vigouroux, C., Bastard, J. P., & Capeau, J. (2014). Emerging clinical issues related to management of multiorgan comorbidities and polypharmacy. *Current Opinion in HIV and AIDS, 9*, 371-378.

Viktil, K. K., Blix, H. S., Moger, T. A., & Reikvam, A. (2007). Polypharmacy as commonly defined is an indicator of limited value in the assessment of drug-related problems. *British journal of clinical pharmacology, 63*(2), 187-195. doi:10.1111/j.1365-2125.2006.02744.x

Viktil KK, Blix HS, Reikvam A. The Janus face of polypharmacy - overuse versus underuse of medication. Norsk Epidemiologi 2008;18(2):147-52.

Vivas-Consuelo, D., Uso-Talamantes, R., Trillo-Mata, J. L., & Mendez-Valera, P. (2015). Methods to control the pharmaceutical cost impact of chronic conditions in the elderly. *Expert Review of Pharmacoeconomics and Outcomes Research, 15*(3), 425-437.

Walsh, E. K., & Cussen, K. (2010). "Take ten minutes": a dedicated ten minute medication review reduces polypharmacy in the elderly. *Irish medical journal, 103*(8), 236-238.

Wang, R., Chen, L., Fan, L., Gao, D., Liang, Z., He, J., . . . Gao, L. (2015). Incidence and Effects of Polypharmacy on Clinical Outcome among Patients Aged 80+: A Five-Year Follow-Up Study. *PLoS ONE, 10*(11), e0142123. doi:10.1371/journal.pone.0142123

Wauters, M., Elseviers, M., Vaes, B., Degryse, J., Dalleur, O., Vander Stichele, R., . . . Azermai, M. (2016). Too many, too few, or too unsafe? Impact of inappropriate prescribing on mortality, and hospitalization in a cohort of community-dwelling oldest old. *British journal of clinical pharmacology, 82*(5), 1382-1392. doi:10.1111/bcp.13055

Wauters, M., Elseviers, M., Vaes, B., Degryse, J., Dalleur, O., Vander Stichele, R., . . . Azermai, M. (2016). Polypharmacy in a Belgian cohort of community-dwelling oldest old (80+). *Acta Clinica Belgica, 71*(3), 158-166. doi:10.1080/17843286.2016.1148298

Wauters, M., Elseviers, M., Vaes, B., Degryse, J., Vander Stichele, R., Christiaens, T., & Azermai, M. (2016). Mortality, hospitalisation, institutionalisation in community-dwelling oldest old: The impact of medication. *Archives of Gerontology and Geriatrics, 65*, 9-16.

Wawruch, M., Fialova, D., Zikavska, M., Wsolova, L., Jezova, D., Kuzelova, M., . . . Krajcik, S. (2008). Factors influencing the use of potentially inappropriate medication in older patients in Slovakia. *Journal of Clinical Pharmacy and Therapeutics, 33*, 381-392.

Wawruch, M., Zikavska, M., Wsolova, L., Kuzelova, M., Tisonova, J., Gajdosik, J., . . . Kristova, V. (2008). Polypharmacy in elderly hospitalised patients in Slovakia. *Pharmacy World and Science, 30*, 235-242.

Wehling, M. (2009). Multimorbidity and polypharmacy: how to reduce the harmful drug load and yet add needed drugs in the elderly? Proposal of a new drug classification: fit for the aged. *Journal of the American Geriatrics Society, 57*(3), 560-561.

Wehling, M., & Throm, C. (2015). [Polypharmacy in the elderly - choosing wisely by using the FORTA list]. *Dtsch Med Wochenschr, 140*(18), 1378-1382. doi:10.1055/s-0041-103205

Wehrberger, C., Madersbacher, S., Jungwirth, S., Fischer, P., & Tragl, K. H. (2012). Lower urinary tract symptoms and urinary incontinence in a geriatric cohort - A population-based analysis. *BJU International, 110*, 1516-1521.

Westerholm, B. (2013). Providing value for medicines in older people. *Medical Writing, 22*(4), 292-295. doi:10.1179/2047480613z.000000000150

Williams, M. E., Pulliam, C. C., Hunter, R., Johnson, T. M., Owens, J. E., Kincaid, J., . . . Koch, G. (2004). The short-term effect of interdisciplinary medication review on function and cost in ambulatory elderly people. *Journal of the American Geriatrics Society, 52*(1), 93-98.

Wilson, N. M., Hilmer, S. N., March, L. M., Cameron, I. D., Lord, S. R., Seibel, M. J., . . . Sambrook, P. N. (2011). Associations between drug burden index and falls in older people in residential aged care. *Journal of the American Geriatrics Society, 59*, 875-880.

Wimmer, B. C., Dent, E., Bell, J. S. S., Wiese, M. D., Chapman, I., Johnell, K., & Visvanathan, R. (2014). Medication Regimen Complexity and Unplanned Hospital Readmissions in Older People. *Annals of Pharmacotherapy, 48*, 1120-1128.

Wimmer, B. C., Dent, E., Visvanathan, R., Wiese, M. D., Johnell, K., Chapman, I., & Bell, J. S. (2014). Polypharmacy and medication regimen complexity as factors associated with hospital discharge destination among older people: A prospective cohort study. *Drugs and Aging, 31*, 623-630.

Wise, J. (2013). Polypharmacy: a necessary evil. *BMJ, 347*, f7033. doi:10.1136/bmj.f7033

Womack, J. A., Brandt, C. A., & Justice, A. C. (2015). Primary Care of Women Aging with HIV. *Journal of Midwifery and Women's Health, 60*(2), 146-157.

Wong, J., Marr, P., Kwan, D., Meiyappan, S., & Adcock, L. (2014). Identification of inappropriate medication use in elderly patients with frequent emergency department visits. *Canadian Pharmacists Journal, 147*, 248-256.

Wright, R. M., Sloane, R., Pieper, C. F., Ruby-Scelsi, C., Twersky, J., Schmader, K. E., & Hanlon, J. T. (2009). Underuse of indicated medications among physically frail older US veterans at the time of hospital discharge: results of a cross-sectional analysis of data from the Geriatric Evaluation and Management Drug Study. *Am J Geriatr Pharmacother, 7*(5), 271-280. doi:10.1016/j.amjopharm.2009.11.002

Wu, J. Y., Leung, W. Y., Chang, S., Lee, B., Zee, B., Tong, P. C., & Chan, J. C. (2006). Effectiveness of telephone counselling by a pharmacist in reducing mortality in patients receiving polypharmacy: randomised controlled trial. *BMJ, 333*(7567), 522. doi:10.1136/bmj.38905.447118.2F

Wyles, H., & Rehman, H. U. (2005). Inappropriate polypharmacy in the elderly. *European Journal of Internal Medicine, 16*, 311-313.

Yang, D. C., Lee, J. D., Huang, C. C., Shih, H. I., & Chang, C. M. (2015). Association between multiple geriatric syndromes and life satisfaction in community-dwelling older adults: A nationwide study in Taiwan. *Archives of Gerontology and Geriatrics, 60*(3), 437-442.

Yong, T. Y., Lau, S. Y., Li, J. Y., Hakendorf, P., & Thompson, C. H. (2012). Medication prescription among elderly patients admitted through an acute assessment unit. *Geriatr Gerontol Int, 12*(1), 93-101. doi:10.1111/j.1447-0594.2011.00737.x
